# N-glycosylated LTβR increases the Th17/Treg cell ratio in liver cancer by blocking RORC ubiquitination and FOXP3 transcription

**DOI:** 10.1038/s41419-025-07738-2

**Published:** 2025-05-28

**Authors:** Banglun Pan, Yuxin Yao, Hao Wu, Dongjie Ye, Zhu Zhang, Xinyu Zhang, Xiaoqian Wang, Nanhong Tang

**Affiliations:** 1 Department of Hepatobiliary Surgery and Fujian Institute of Hepatobiliary Surgery, Fujian Medical University Union Hospital, Fuzhou, China; 2 Cancer Center of Fujian Medical University, Fujian Medical University Union Hospital, Fuzhou, China; 3 Key Laboratory of Clinical Laboratory Technology for Precision Medicine (Fujian Medical University), Fujian Province University, Fuzhou, China; 4 Key Laboratory of Ministry of Education for Gastrointestinal Cancer, Fujian Medical University, Fuzhou, China

**Keywords:** Cell signalling, Immunosurveillance

## Abstract

LTβR-overexpressing CAR-T cells have demonstrated surprising effectiveness against solid tumors, exhibiting strong anti-exhaustion and proliferation capabilities. However, the role of LTβR in CD4^+^ T cell differentiation and anti-tumor activity remains unclear. In this study, we employed primary or subcutaneous mouse hepatocellular carcinoma (HCC) models and flow cytometry to study the impact of conditional knock-in of *Ltbr* on CD4^+^ T cell differentiation and response, particularly the Th17/Treg cell ratio, and its influence on HCC progression. Immunoprecipitation, immunoblotting, RT-qPCR, molecular docking, and Chromatin Immunoprecipitation-qPCR were performed to investigate the molecular mechanism of CD4^+^ T cell differentiation. Adeno-associated virus-modified T cells were introduced into patient-derived orthotopic xenograft (PDOX) model to assess the combined impact of LTβR and glycolysis inhibitors on the Th17/Treg cell differentiation. We found that LTβR reduced PELI1 expression, preventing TRAF3 protein degradation in Th17 cells. TRAF3 then competed with RORC for SMURF1 binding, enhancing RORC stability and Th17 cell differentiation. LTβR also blocked PRDM1 expression, delaying *Foxp3* transcription and Treg cell infiltration. Additionally, N-glycosylation supported the stability of LTβR by protecting it from ubiquitination. From a therapeutic perspective, glycolysis inhibitors helped LTβR balance the proportion of Th17/Treg cells in PDOX model to inhibit tumor growth. In conclusion, our findings indicated that LTβR N-glycosylation prevented RORC ubiquitination and *Foxp3* transcription, raising the Th17/Treg cell ratio and hindering HCC progression.

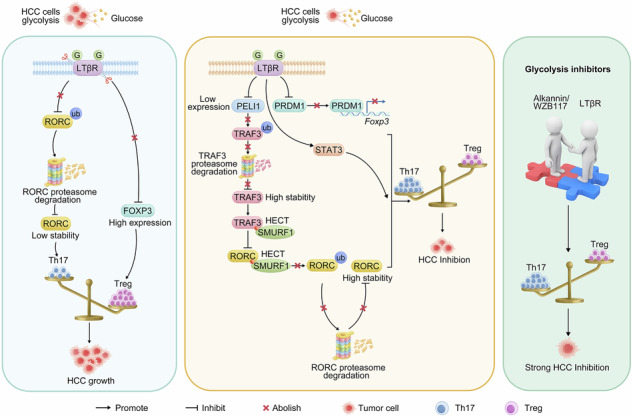

## Introduction

LTβR, part of the tumor necrosis factor receptor superfamily, is a cell surface receptor for lymphotoxins that triggers apoptosis and cytokine release [1]. It is found on various non-immune and myeloid-derived immune cells, but little on lymphocytes [1]. Ectopically expressed LTβR promotes T cell proliferation, maintains youthful, stem-like T cell features, and prevents their exhaustion [1]. While LTβR-introduced CAR-T cells show strong cytotoxicity and proliferation, their impact on CD4^+^ T cell differentiation requires further investigation.

Th17 cells are common in the tumor microenvironment (TME), but their role in tumor immunity is debated [2, 3]. Excessive Th17 cell-mediated inflammation causes cancer [2], yet adoptive transfer studies indicated mouse Th17 cells have anticancer effects [3]. Their role in promoting or inhibiting cancer is highly tumor-dependent [4]. Understanding the role of Th17 cells in tumor immunity is vital for cancer immunotherapies. What’s more, high Treg cell presence in tumors indicates poor prognosis, as they secrete cytokines aiding tumor immune escape and growth [5]. Therefore, investigating the molecular mechanisms behind Th17 and Treg cell differentiation is key to grasping the TME and enhancing T cell-based anti-tumor therapies.

RORC serves as the pivotal transcription factor essential for both the differentiation and functional maintenance of Th17 cells, exerting anti-tumor immunity through the regulation of inflammatory factor expression, including IL17A [6]. Th17 cells can augment the tumor-suppressing immune response by activating CD8^+^ T cells and NK cells [7]. Furthermore, RORC is critical for preserving the lineage stability of Th17 cells, preventing their conversion into Th2 cells by modulating the epigenetic modification of super-enhancers [8]. Research indicated that RORC deficiency results in the aberrant expression of Th2-associated molecules, such as IL4, within Th17 cells, thereby diminishing their anti-tumor efficacy [8].

In this study, we established primary and subcutaneous mouse models of hepatocellular carcinoma (HCC) featuring a CD4^+^ T cell-conditional knock-in (cKI) of *Ltbr*. Our findings indicated that this modification suppressed tumor growth by augmenting the Th17/Treg cell ratio. Mechanically, LTβR stabilized TRAF3 protein by reducing its E3 ubiquitin ligase *Peli1* transcription. TRAF3 competed with RORC for SMURF1, preventing RORC degradation and promoting Th17 cell differentiation. Additionally, LTβR inhibited transcription factor *Prdm1* transcription, reducing transcription initiation of *Foxp3*. Finally, we discovered that HCC cells used glycolysis to consume glucose, hindering LTβR’s N-glycosylation and stability. Notably, glycolysis inhibitors approved by the Food and Drug Administration (FDA) improved immunotherapy susceptibility via LTβR.

## Methods and materials

Detailed methods are listed in Supplemental Information.

## Results

### LTβR raised the Th17/Treg cell ratio and suppressed HCC growth

Research has demonstrated that LTβR, typically low expressed in lymphocytes, exhibits a potent anti-tumor effect [9]. Inducing the expression of LTβR in CD8^+^ T cells has been shown to enhance their proliferation and cytotoxic capabilities, thereby improving the effectiveness of CAR-T therapy in the treatment of solid tumors [9]. However, the role of LTβR in CD4^+^ T cell differentiation and tumor immunity is unclear. Initially, we conducted an analysis of the impact of LTβR on chromatin accessibility and gene expression in CD4^+^ T cells utilizing the publicly accessible dataset GSE193736 [9]. Our findings indicated that LTβR overexpression in TCR-stimulated CD4^+^ T cells significantly modified chromatin accessibility and transcriptional expression profiles (Fig. S1A). We observed 38 genes with enhanced chromatin accessibility and mRNA expression (Fig. S1B). The up-regulated genes were mapped to the pathways associated with Th17 cell signaling and protein deubiquitination (Fig. S1C). Significantly, LTβR enhanced chromatin accessibility and transcriptomic signals at the STAT3 locus associated with Th17 cells, while it reduced these signals at the FOXP3 locus associated with Treg cells in TCR-stimulated CD4^+^ T cells (Fig. S1D). However, it did not influence chromatin accessibility or the transcriptional activity of the Th17 cell signature markers, RORC and IL17A (Fig. S1D). Based on these observations, we hypothesized that LTβR upregulates Th17 cell differentiation at the level of protein post-translational modification. Moreover, considering LTβR’s significant role in the regulation of protein deubiquitination (Fig. S1C), we proposed that it could influence the Th17/Treg cell ratio by modulating the ubiquitination and subsequent degradation of Th17-related proteins.

**Fig. 1:**
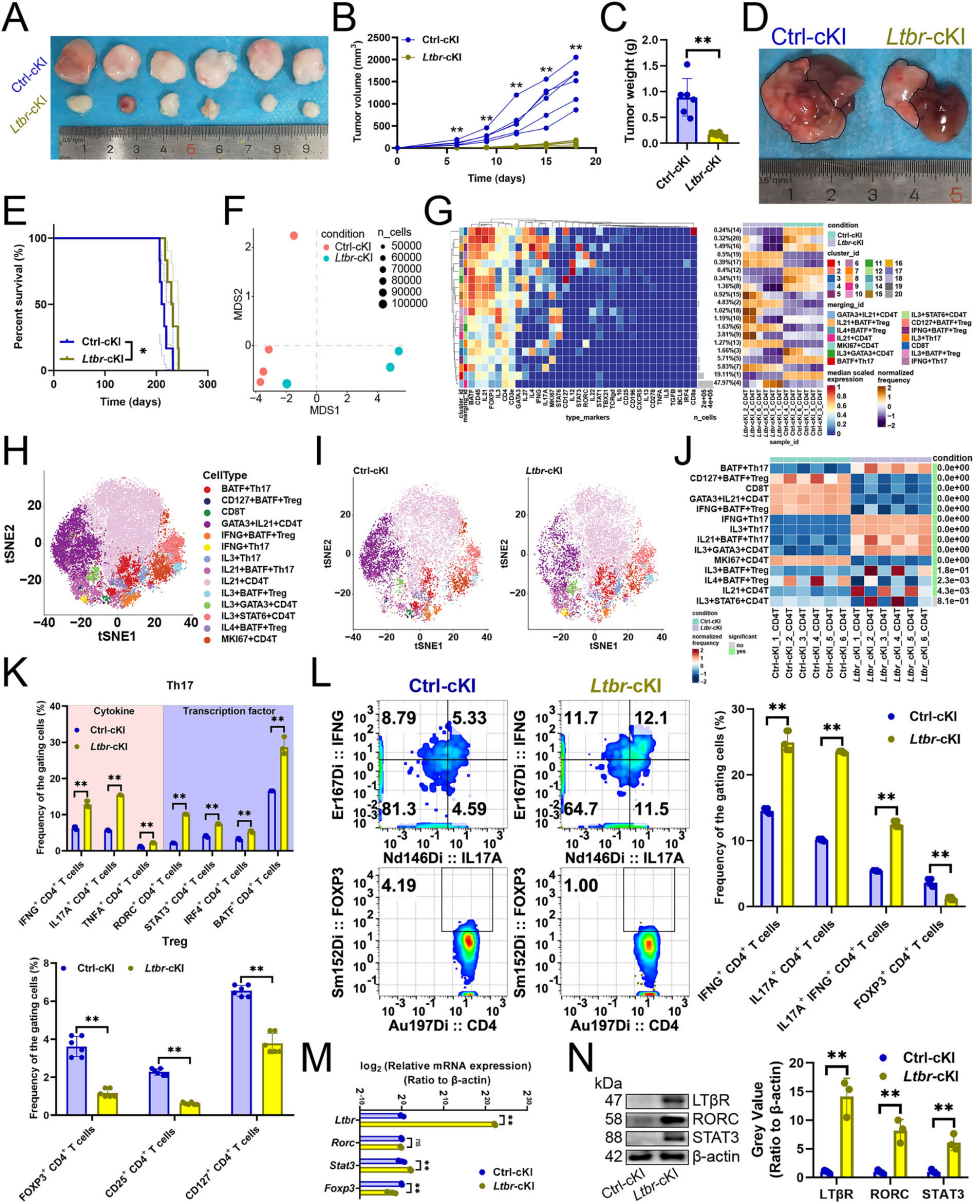
LTβR raised the Th17/Treg cell ratio and suppressed hepatocellular carcinoma growth. **A**–**C** *Ltbr*-cKI in CD4^+^ T cells affecting subcutaneous tumor growth (*n* = 6). **A** Representative. **B** Growth curves. **C** Tumor weights. **D, E** *Ltbr*-cKI in CD4^+^ T cells affecting primary carcinoma growth (*n* = 6). **D** Representative. **E** Survival curves. **F** Non-metric multidimensional scaling analysis showed CD4^+^ T cell antigen expression similarity pre- and post-*Ltbr*-cKI (*n* = 6). **G** A heatmap displayed median antigen expression levels for SOMs (*n* = 6). **H** A SOM superimposed on mass cytometry data of primary carcinoma-infiltrating CD4^+^ T cells (*n* = 6). **I** A mosaic plot showed single CD4^+^ T cells pre- and post-*Ltbr*-cKI (*n* = 6). **J** A heatmap displayed CD4^+^ T cell subset proportions pre- and post-*Ltbr*-cKI (*n* = 6). **K** The upper section showed the expression of Th17 cell-specific cytokines and transcription factors in CD4^+^ T cells via flow cytometry, while the lower section presented the levels of Treg cell-specific indicators (*n* = 6). **L** Flow cytometry revealed Th17 and Treg cell infiltration (*n* = 6). **M**, **N** RT-qPCR (**M**) and immunoblotting (**N**) assessed LTβR’s impact on T cell-associated gene expressions in CD4^+^ T cells (*n* = 3). **B**, **C**, **J–N** represented mean ± SD analyzed by unpaired *t* test. **E** was analyzed by Log-rank test, (**G**) was analyzed by Euclidean Distance Clustering Algorithm. *P < 0.05, **P < 0.01. cKI, conditional knock-in; SOM, self-organizing map.

Next, we conditionally knocked *Ltbr* into CD4^+^ T cells (*Ltbr*-cKI) and assessed its anti-tumor effects, finding that these CD4^+^ T cells inhibited tumor growth (Fig. 1A–D) and improved prognosis (Fig. 1E). Mass cytometry further detailed *Ltbr*-cKI’s effects on CD4^+^ T cell subset composition. Non-metric multidimensional scaling analysis indicated that *Ltbr*-cKI significantly modified CD4^+^ T cell function (Fig. 1F). A hierarchical clustering heatmap was then employed to visualize CD4^+^ T cell antigen expressions, identifying 13 subsets (Fig. 1G), including BATF^+^ Th17 cells, CD127^+^ BATF^+^ Treg cells, GATA3^+^ IL21^+^ CD4^+^ T cells, IFNG^+^ BATF^+^ Treg cells, IFNG^+^ Th17 cells, IL3^+^ Th17 cells, IL21^+^ BATF^+^ Th17 cells, IL21^+^ CD4^+^ T cells, IL3^+^ BATF^+^ Treg cells, IL3^+^ GATA3^+^ CD4^+^ T cells, IL3^+^ STAT6^+^ CD4^+^ T cells, IL4^+^ BATF^+^ Treg cells, and MKI67^+^ CD4^+^ T cells (Fig. 1H). *Ltbr*-cKI increased BATF^+^ Th17 cell, IFNG^+^ Th17 cell, IL3^+^ Th17 cell, IL21^+^ BATF^+^ Th17 cell, IL3^+^ GATA3^+^ CD4^+^ T cell, and IL21^+^ CD4^+^ T cell infiltration, and decreased CD127^+^ BATF^+^ Treg cell, GATA3^+^ IL21^+^ CD4^+^ T cell, IFNG^+^ BATF^+^ Treg cell, MKI67^+^ CD4^+^ T cell, and IL4^+^ BATF^+^ Treg cell levels (Fig. 1I, J). Moreover, *Ltbr*-cKI elevated Th17 cell cytokines IFNG, IL17A, and TNFA expression in CD4^+^ T cells, and transcription factors RORC, STAT3, IRF4, and BATF levels, while reducing Treg cell markers FOXP3, CD25, and CD127 expression (Fig. 1K, S2A, S2B). Additionally, *Ltbr*-cKI increased the Th17 cell infiltration and reduced Treg cell abundance (Fig. 1L). In addition, we observed increased STAT3 transcript and protein levels in CD4^+^ T cells with *Ltbr*-cKI, decreased FOXP3 transcript and protein levels, and an increase in RORC protein levels, but not its transcript (Fig. 1M, N). Therefore, we believed that LTβR was an important factor in increasing the Th17/Treg cell ratio.

### LTβR inhibited PELI1 expression to improve TRAF3 protein stability

**Fig. 2:**
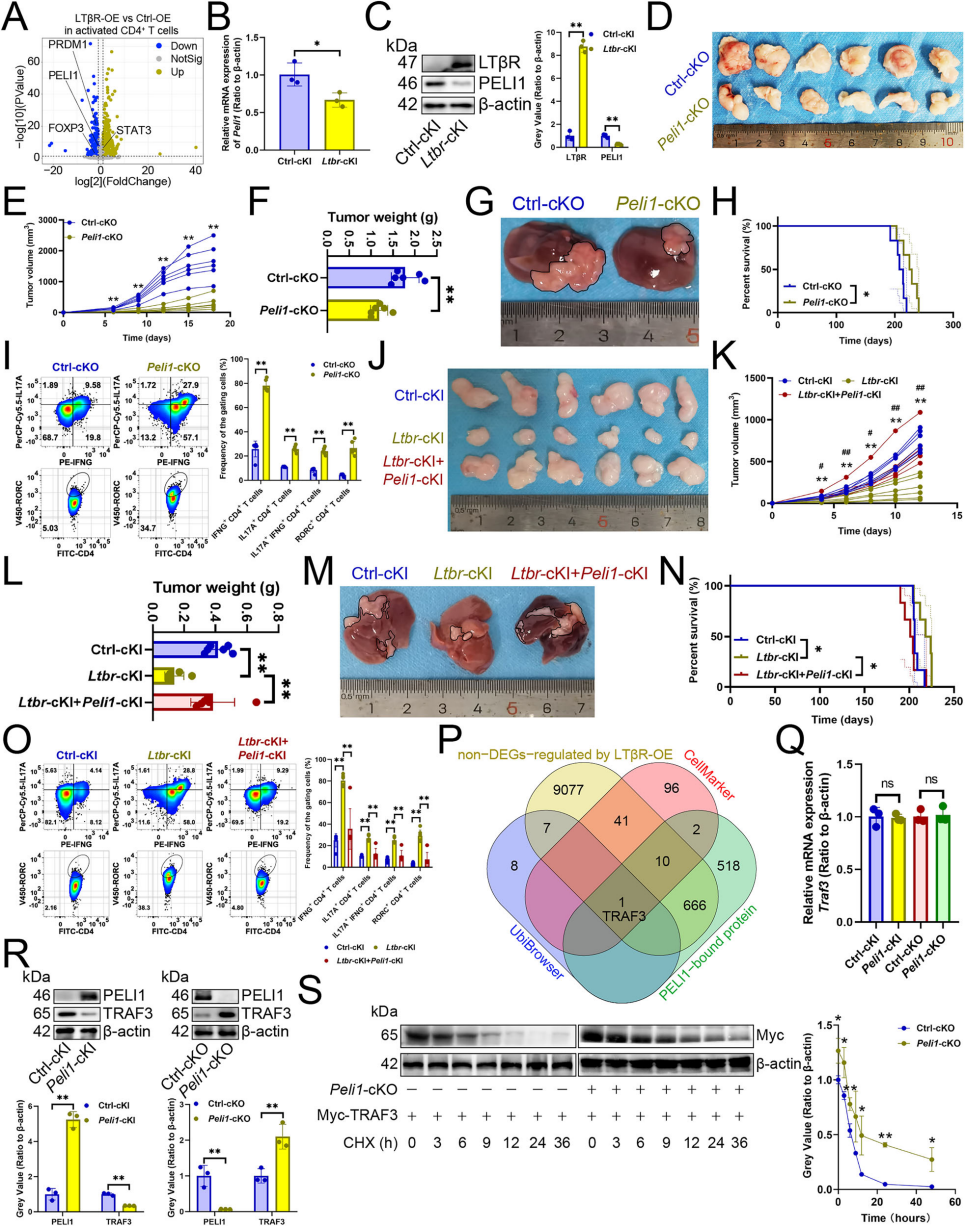
LTβR inhibited PELI1 expression to improve TRAF3 protein stability. **A** A volcano plot illustrated transcript changes in LTβR-overexpressing CD4^+^ T cells using publicly accessible dataset GSE193736 (*n* = 3). RT-qPCR (**B**) and immunoblotting (**C**) demonstrated the impact of *Ltbr*-cKI in Th17 cells on *Peli1* mRNA expression (*n* = 3). **D–F** *Peli1*-cKO in Th17 cells affecting subcutaneous tumor growth (*n* = 6). **D** Representative. **E** Growth curves. **F** Tumor weights. **G**, **H** *Peli1*-cKO in Th17 cells affecting primary carcinoma growth (*n* = 6). **G** Representative. **H** Survival curves. **I** Flow cytometry revealed the impact of Th17 cell-specific *Peli1*-cKO on Th17 cell infiltration and RORC expression (*n* = 6). **J**–**L** *Ltbr*- or *Peli1*-cKI in Th17 cells affecting subcutaneous tumor growth (*n* = 6). **J** Representative. **K** Growth curves. * was between “*Ltbr*-cKI” and “Ctrl-cKI”. # was between “*Ltbr*-cKI+*Peli1*-cKI” and “*Ltbr*-cKI”. **L** Tumor weights. **M**, **N** *Ltbr*- or *Peli1*-cKI in Th17 cells affecting primary carcinoma growth (*n* = 6). **M** Representative. **N** Survival curves. **O** Flow cytometry revealed the impact of Th17 cell-specific *Ltbr*- or *Peli1*-cKI on Th17 cell infiltration and RORC expression (*n* = 6). **P** A Venn diagram highlighted the overlap among transcripts unaffected by LTβR overexpression in CD4^+^ T cells, CD4^+^ T cell markers from CellMarker, PELI1 substrates predicted by UbiBrowser, and PELI1-bound proteins identified by mass spectrometry. **Q**, **R** RT-qPCR (**Q**) and immunoblotting (**R**) demonstrated how *Peli1*-cKI or -cKO affected TRAF3 expression in Th17 cells (*n* = 3). **S** Th17 cells with Myc-tagged TRAF3 were treated with Cycloheximide to assess TRAF3 protein expression (*n* = 3). **A**–**C**, **E**, **F**, **I**, **K**, **L**, **O**, **Q**–**S** represented mean ± SD analyzed by unpaired *t* test. **H**, **N** were analyzed by Log-rank test. *P < 0.05, **P < 0.01, #*P* < 0.05, ##*P* < 0.01. cKI conditional knock-in, cKO conditional knockout.

Given that LTβR was linked to pathways involved in Th17 cell signaling and protein deubiquitination, yet did not lead to an upregulation of the RORC transcript (Fig. 1M, S1C, S1D), we hypothesized that LTβR stabilizes the RORC protein by inhibiting its ubiquitination. We examined the transcriptome of CD4^+^ T cells with overexpressed LTβR [9] and observed that among E3 ubiquitin ligases or deubiquitinases, *Otud1* and *Peli1* mRNA expression was decreased, but *Traf6*, *Usp18*, and *Usp53* mRNA levels were increased (Fig. S3A). Mass spectrometry (MS) indicated that only TRAF6 and USP53 interacted with RORC (Fig. S3B). However, TRAF6 has been reported to inhibit Th17 cell differentiation (which was not consistent with the expectation that LTβR stabilized RORC proteins by inhibiting TRAF6 expression) [10], and USP53 is inactive [11]. Thus, we proposed LTβR promotes Th17 cell differentiation by ubiquitinating an intermediate (OTUD1, USP18, and PELI1) influencing RORC protein expression. We then knocked out *Otud1*, *Peli1*, and *Usp18* in Th17 cells and found that *Otud1*- or *Usp18*- knockout (KO) reduced RORC protein expression, while *Peli1*-KO increased it (Fig. S3C). Due to the inhibition of OTUD1, a RORC stabilizer, by LTβR overexpression—contrary to its anticipated promoting effect—and the promotion of USP18, another RORC stabilizer, by LTβR overexpression, along with the well-established relationship between USP18 and Th17 cell differentiation [12], both factors were excluded from our analysis, leading us to formulate a hypothesis that LTβR enhances RORC protein expression by blocking the PELI1-mediated ubiquitination pathway. Next, we investigated PELI1’s impact on Th17 cell differentiation and found that *Ltbr*-cKI reduced PELI1 transcript and protein expression (Fig. 2A–C). Conditional KO of *Peli1* (*Peli1*-cKO) in Th17 cells curbed tumor growth (Fig. 2D-G), improved prognosis (Fig. 2H), and increased Th17 cell infiltration (Fig. 2I, S3D). However, the antitumor effect and Th17 infiltration promoted by *Ltbr*-cKI in Th17 cells were diminished by *Peli1*-cKI in Th17 cells (Fig. 2J–O).

Since PELI1 is an E3 ubiquitin ligase [13], we looked for its substrates that induce Th17 cell differentiation. By intersecting transcripts unaffected by LTβR overexpression in CD4^+^ T cells [9], CD4^+^ T cell markers from CellMarker [14], PELI1 substrates predicted by UbiBrowser [15], and PELI1-binding proteins identified by MS, we identified TRAF3 as the only potential substrate of PELI1 that might promote Th17 cell differentiation (Fig. 2P). Moreover, conditional KO of *Traf3* (*Traf3*-cKO) in Th17 cells decreased RORC protein stability (Fig. S3E). Therefore, we hypothesized that PELI1 ubiquitinates and degrades TRAF3 protein. Conditional KI of *Peli1* (*Peli1*-cKI) in Th17 cells was witnessed to lower TRAF3 protein levels without affecting its transcription, while *Peli1*-cKO had the opposite effect (Fig. 2Q, R). Additionally, *Peli1*-cKO enhanced TRAF3 protein stability when stimulated with Cycloheximide (Fig. 2S). Consequently, it was posited that LTβR attenuated the ubiquitination-mediated degradation of TRAF3 by suppressing *Peli1* transcription, thereby indirectly stabilizing RORC, a pivotal factor in Th17 cell differentiation.

### PELI1 regulated the inhibition of Th17 cell infiltration by deleting TRAF3

**Fig. 3:**
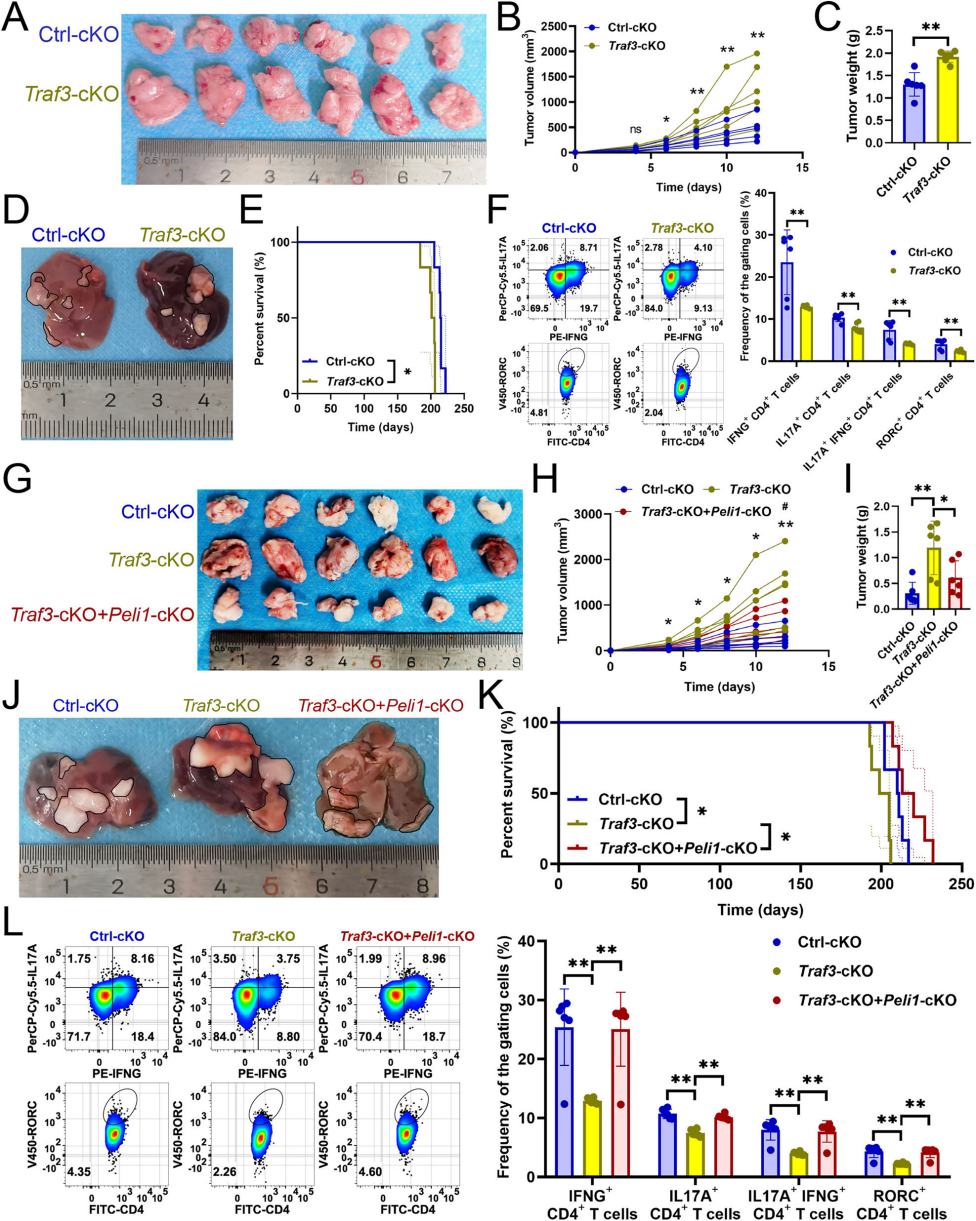
PELI1 regulated the inhibition of Th17 cell infiltration by deleting TRAF3. **A**–**C** *Traf3*-cKO in Th17 cells affecting subcutaneous tumor growth (*n* = 6). **A** Representative. **B** Growth curves. **C** Tumor weights. **D**, **E** *Traf3*-cKO in Th17 cells affecting primary carcinoma growth (*n* = 6). **D** Representative. **E** Survival curves. **F** Flow cytometry revealed the impact of Th17 cell-specific *Traf3*-cKO on Th17 cell infiltration and RORC expression (*n* = 6). **G**–**I** *Traf3*- or *Peli1*-cKO in Th17 cells affecting subcutaneous tumor growth (*n* = 6). **G** Representative. **H** Growth curves. * was between “*Traf3*-cKO” and “Ctrl-cKO”. # was between “*Traf3*-cKO+*Peli1*-cKO” and “*Traf3*-cKO”. **I** Tumor weight. **J**, **K** *Traf3*- or *Peli1*-cKO in Th17 cells affecting primary carcinoma growth (*n* = 6). **J** Representative. **K** Survival curves. **L** Flow cytometry revealed the impact of Th17 cell-specific *Traf3*- or *Peli1*-cKO on Th17 cell infiltration and RORC expression (*n* = 6). **B**, **C**, **F**, **H**, **I**, **L** represented mean ± SD analyzed by unpaired *t* test. **E**, **K** were analyzed by Log-rank test. *P < 0.05, **P < 0.01, #*P* < 0.05, ##*P* < 0.01. cKO conditional knockout.

Our analysis of the PELI1-TRAF3 axis in Th17 cell-driven anti-tumor immunity revealed that *Traf3*-cKO in Th17 cells promoted tumor growth (Fig. 3A–D), worsened prognosis (Fig. 3E), and reduced Th17 cell infiltration (Fig. 3F). Conversely, *Peli1*-cKO in Th17 cells mitigated the tumor-promoting effects of *Traf3*-cKO (Fig. 3G–K) and improved Th17 cell differentiation (Fig. 3L).

### PELI1 facilitated K48-linked TRAF3 ubiquitination

**Fig. 4:**
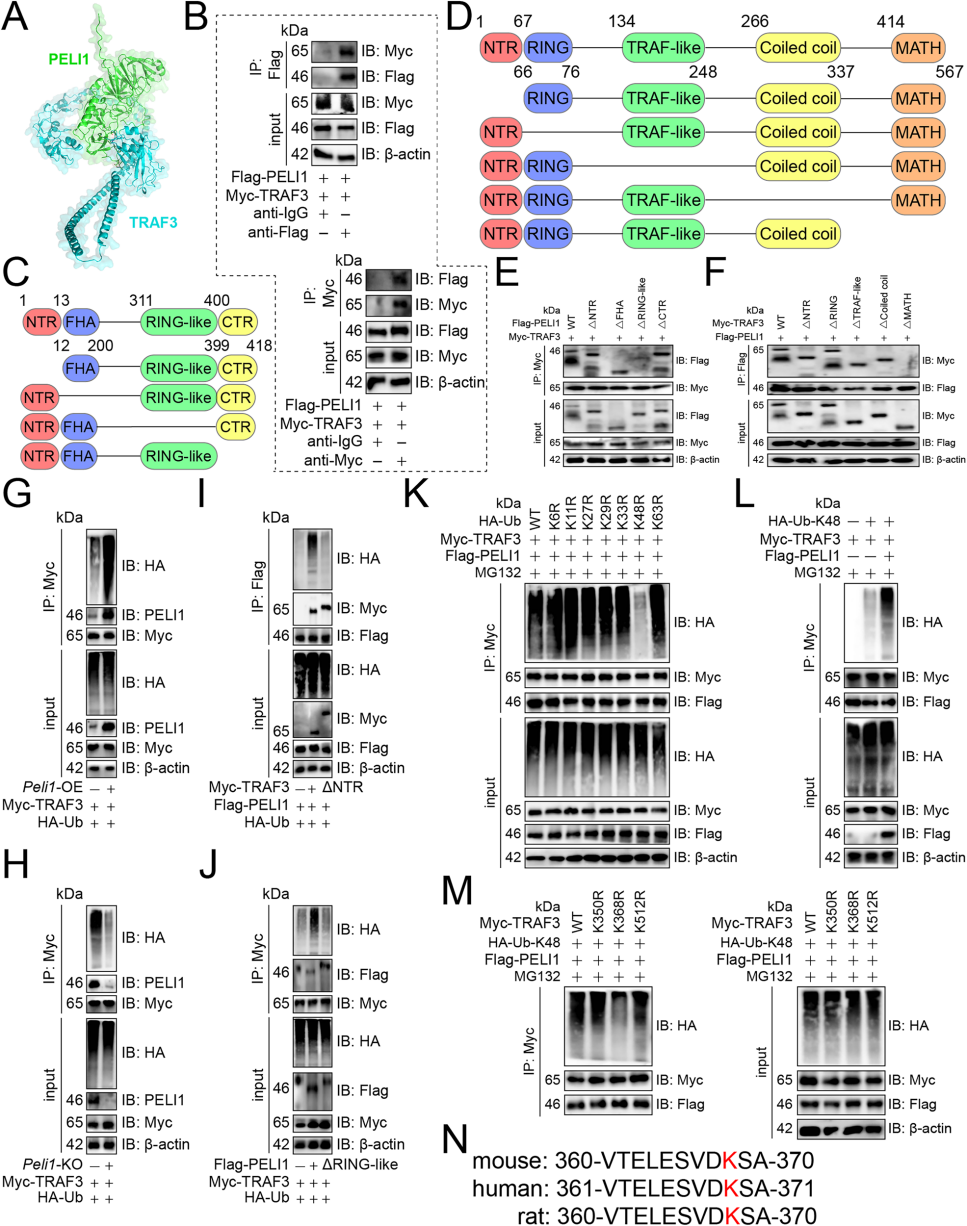
PELI1 facilitated K48-linked TRAF3 ubiquitination. **A** A surface plot showed the docking model and interface residues between PELI1 and TRAF3 proteins. **B** Th17 cell lysates were treated with anti-IgG control and -Flag (upper panel) or -Myc (lower panel) antibodies, using 5% lysate as input control. **C**, **D** Schematic diagrams showed domain deletion mutants of PELI1 (**C**) and TRAF3 (**D**). **E**, **F** Immunoprecipitation revealed key binding domains for PELI1 and TRAF3. Th17 cells were co-transfected with Myc-tagged TRAF3 and Flag-tagged PELI1 wild-type or deletion mutants (**E**) and vice versa (**F**). **G**, **H** The impact of *Peli1* overexpression (**G**) or knockout (**H**) on TRAF3 ubiquitination in Th17 cells. **I**, **J** The influence of TRAF3’s NTR domain (**I**) and PELI1’s RING-like domain (**J**) on TRAF3 ubiquitination in Th17 cells. **K** Analysis of TRAF3’s ubiquitination chains in Th17 cells. **L** PELI1’s effect on ubiquitin K48-linked TRAF3 ubiquitination in Th17 cells. **M** Ubiquitination of four Myc-tagged TRAF3 lysine mutants in Th17 cells. **N** Conservation of the K368 across species. Red indicated lysine sites that undergo ubiquitination.

We then analyzed how PELI1 is specifically bound to TRAF3. We explored PELI1’s binding to TRAF3 using molecular docking and immunoprecipitation, confirming their interaction (Fig. 4A, B). Domain deletion mutants revealed that PELI1’s RING-like domain bound to TRAF3’s MATH domain (Fig. 4C–F). Furthermore, the overexpression of *Peli1* increased the ubiquitination and subsequent degradation of TRAF3 in Th17 cells, whereas the absence of *Peli1* produced the opposite effect (Fig. 4G, H). TRAF3’s MATH domain was essential for complex formation, and its removal reduced its ubiquitination in Th17 cells (Fig. 4I). Similarly, PELI1’s RING-like domain was crucial for binding, and its absence also hampered TRAF3 ubiquitination in Th17 cells (Fig. 4J). Mutating ubiquitin’s lysine to arginine showed that only K48R blocked TRAF3 ubiquitination (Fig. 4K), and *Peli1* overexpression increased K48-linked TRAF3 ubiquitination in Th17 cells (Fig. 4L). We then examined the specific sites of TRAF3 ubiquitination. Lysine-to-arginine mutants of TRAF3’s potential ubiquitination sites were created according to PhosphoSitePlus [16], and their ubiquitination was assessed. Only the K368R prevented TRAF3 ubiquitination in Th17 cells (Fig. 4M), and this site was evolutionarily conserved (Fig. 4N), indicating similar modifications in other organisms. Consequently, we concluded that PELI1 facilitated protein degradation at the K368 residue of ubiquitin-linked TRAF3, specifically at the K48 position.

### TRAF3 competed with RORC for SMURF1 binding

**Fig. 5:**
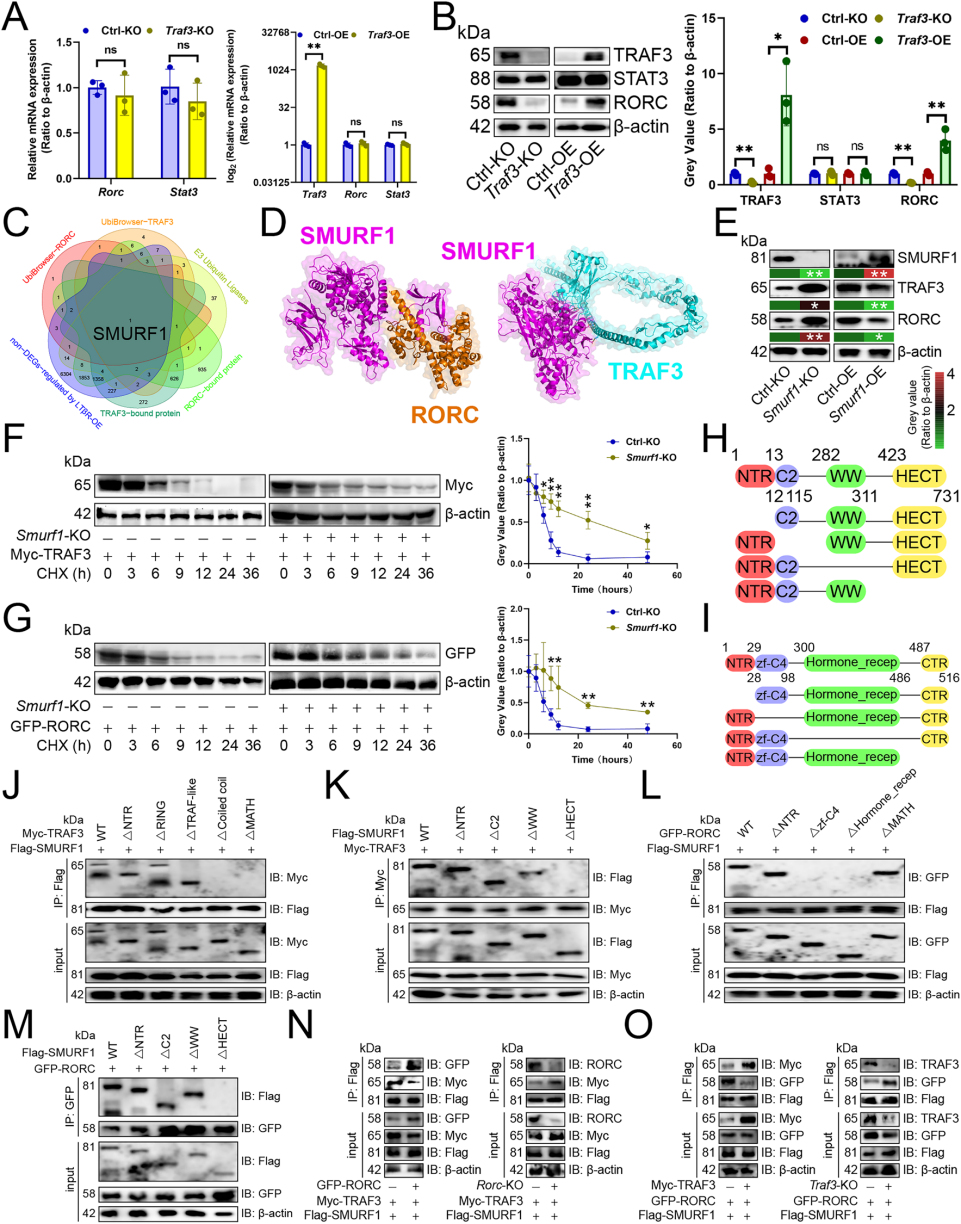
TRAF3 competed with RORC for SMURF1 binding. **A**, **B** RT-qPCR (A) and immunoblotting (**B**) were used to study the impact of *Traf3* knockout or overexpression on STAT3 and RORC levels in Th17 cells (*n* = 3). **C** A Venn diagram illustrated the overlap of E3 ubiquitin ligases predicted by UbiBrowser for TRAF3 or RORC, E3 ubiquitin ligase list, TRAF3- and RORC-bound proteins identified by mass spectrometry, and transcripts unaffected by LTβR overexpression in CD4^+^ T cells. **D** Surface plots depicted the docking model and interface residues between SMURF1 and RORC or TRAF3 proteins. **E** Immunoblotting was employed to analyze how *Smurf1* knockout or overexpression influenced STAT3 or RORC protein levels in Th17 cells (*n* = 3). **F**, **G** Myc-tagged TRAF3 (**F**) and GFP-tagged RORC (**G**) in Th17 cells were treated with Cycloheximide to assess their protein expression (*n* = 3). **H**, **I** Diagrams of SMURF1 (**H**) and RORC (**I**) domain deletion mutants. **J**–**M** Immunoprecipitation revealed crucial binding domains for TRAF3 (**J**) and SMURF1 (**K**) proteins, as well as RORC (**L**) and SMURF1 (**M**) proteins. Th17 cells were co-transfected with Flag-tagged SMURF1 and either Myc-tagged TRAF3 or GFP-tagged RORC, including their wild-type and deletion mutants. **N** Immunoprecipitation showed how altered RORC levels affected SMURF1 binding to TRAF3 in Th17 cells. **O** Immunoprecipitation showed how changing TRAF3 expression impacted SMURF1 binding to RORC in Th17 cells. **A**, **B**, **E**–**G** represented mean ± SD analyzed by unpaired *t* test. *P < 0.05, **P < 0.01.

Furthermore, the mechanism by which TRAF3 up-regulated RORC protein levels is unclear. *Traf3*-KO in Th17 cells decreased RORC protein levels without affecting its transcript levels, while overexpression of *Traf3* resulted in an increase in RORC protein expression without a corresponding elevation in transcript levels (Fig. 5A, B). TRAF3, an E3 ubiquitin ligase, didn’t regulate RORC protein levels via ubiquitination, as *Traf3*-KO reduced RORC protein stability (Fig. 5A, B). We proposed that TRAF3 and RORC compete for the common E3 ubiquitin ligase to stabilize the opponent. By intersecting E3 ubiquitin ligases predicted by UbiBrowser [15] for TRAF3 or RORC, E3 ubiquitin ligase list [15], TRAF3- and RORC-bound proteins identified by MS, and transcripts unaffected by LTβR overexpression in CD4^+^ T cells [9], we identified HECT E3 ubiquitin ligase SMURF1 likely responsible for degrading TRAF3 and RORC protein (Fig. 5C). Molecular docking confirmed SMURF1’s binding to both RORC and TRAF3 (Fig. 5D). We hypothesized that the two substrates competitively bind to the catalytic domain HECT of SMURF1, resulting in both inhibiting each other’s ubiquitination. Our findings indicated that SMURF1 suppressed their protein expression (Fig. 5E) but not their transcription levels (Fig. S4) in Th17 cells. Additionally, *Smurf1*-KO, under Cycloheximide treatment, enhanced the stability of both proteins in Th17 cells (Fig. 5F, G). To determine where RORC or TRAF3 bind to SMURF1, we created their domain deletion mutants (Fig. 5H, I) and discovered that TRAF3’s Coiled coil and MATH domains bound to SMURF1’s HECT domain in Th17 cells (Fig. 5J, K), while RORC’s zf-C4 and Hormone_recep domains bound to SMURF1’s HECT domain (Fig. 5L, M). Moreover, overexpressing *Rorc* reduced SMURF1’s binding to TRAF3 in Th17 cells, whereas *Rorc*-KO increased it (Fig. 5N). Similarly, overexpressing *Traf3* reduced SMURF1’s binding to RORC in Th17 cells, and vice versa (Fig. 5O). Therefore, TRAF3 and RORC competitively bound to SMURF1 to stabilize each other.

### TRAF3 inhibited SMURF1-mediated RORC ubiquitination

**Fig. 6:**
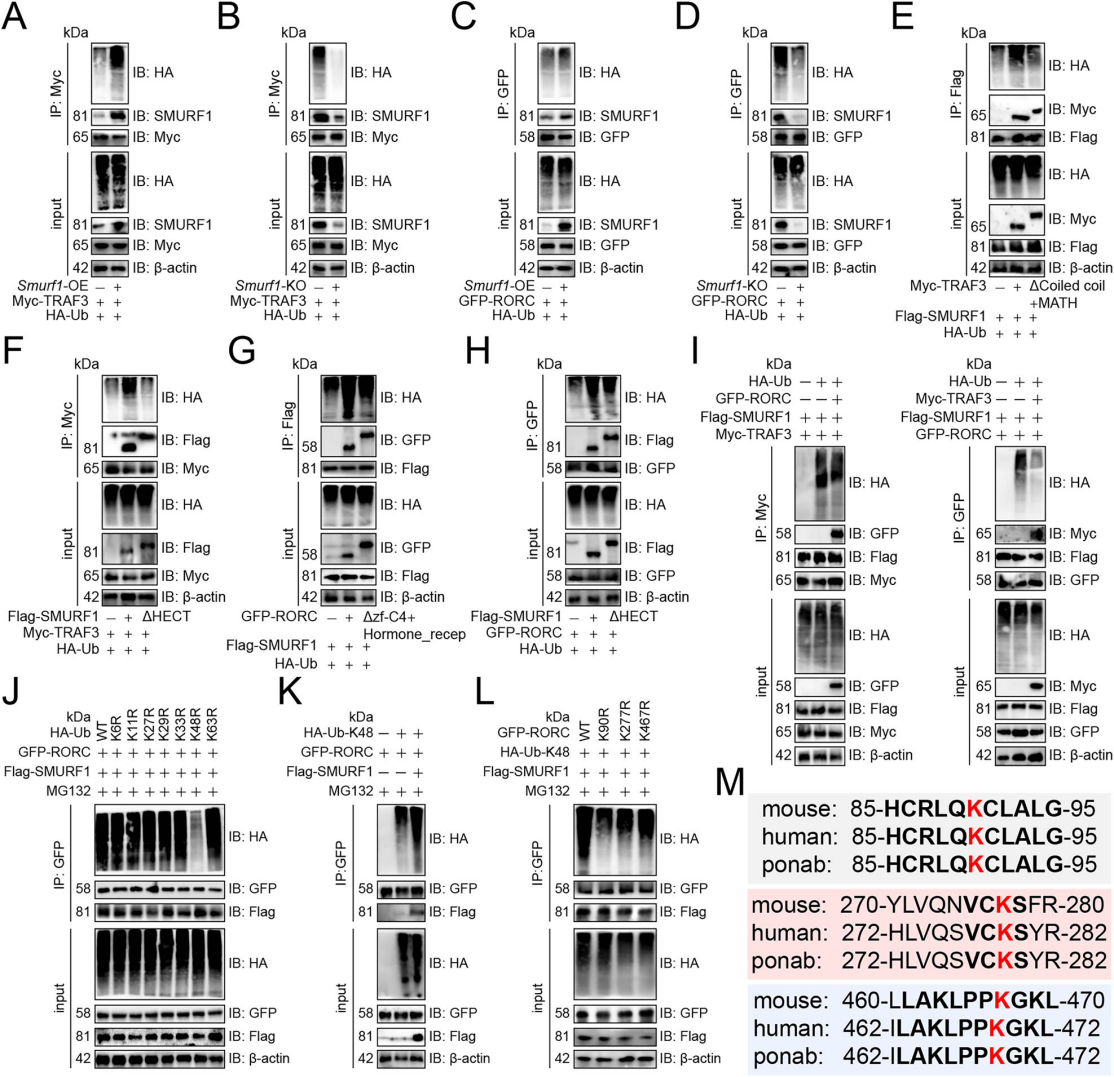
TRAF3 inhibited SMURF1-mediated RORC ubiquitination. **A**–**D** Impact of *Smurf1* overexpression (**A**, **C**) or knockout (**B**, **D**) on TRAF3 (**A**, **B**) or RORC (**C**, **D**) ubiquitination in Th17 cells. **E** Role of TRAF3’s Coiled coil and MATH domains in its ubiquitination in Th17 cells. **F** Effect of SMURF1’s HECT domain on TRAF3 ubiquitination in Th17 cells. **G** Role of RORC’s zf-C4 and Hormone_recep domains in its ubiquitination in Th17 cells. **H** Effect of the SMURF1’s HECT domain on TRAF3 ubiquitination in Th17 cells. **I** Left: RORC’s effect on SMURF1-mediated TRAF3 ubiquitination in Th17 cells. Right: TRAF3’s effect on SMURF1-mediated RORC ubiquitination in Th17 cells. **J** Analysis of TRAF3 ubiquitination chains in Th17 cells. **K** SMURF1’s effect on K48-linked RORC ubiquitination in Th17 cells. **L** Ubiquitination of four GFP-tagged RORC lysine mutants in Th17 cells. **M** Conservation of K90, K277, and K467 across species. Red indicated lysine sites that undergo ubiquitination.

We then examined SMURF1’s role in degrading TRAF3 and RORC proteins. *Smurf1* overexpression increased TRAF3 ubiquitination in Th17 cells (Fig. 6A), and the reverse was also true (Fig. 6B). Additionally, the overexpression of *Smurf1* facilitated the ubiquitination-mediated degradation of RORC (Fig. 6C), whereas its absence led to the degradation of RORC (Fig. 6D). TRAF3’s Coiled coil and MATH domains were essential for forming the TRAF3-SMURF1 complex, and their removal reduced TRAF3 ubiquitination in Th17 cells (Fig. 6E). Similarly, SMURF1’s HECT domain was crucial for binding, and its deletion hindered TRAF3 ubiquitination in Th17 cells (Fig. 6F). RORC’s zf-C4 and Hormone_recep domains were essential for forming the RORC-SMURF1 complex, and their removal reduced RORC ubiquitination in Th17 cells (Fig. 6G). Similarly, SMURF1’s HECT domain was crucial for binding, and its deletion hindered RORC ubiquitination in Th17 cells (Fig. 6H). Notably, overexpressing *Rorc* or *Traf3* reduced the other’s ubiquitination in Th17 cells (Fig. 6I).

We also examined the specific mechanism of RORC ubiquitination. We replaced lysine with arginine in ubiquitin and discovered that only K48R blocked RORC ubiquitination in Th17 cells (Fig. 6J). *Smurf1* overexpression increased K48-linked RORC ubiquitination in Th17 cells (Fig. 6K). We next looked for the specific site of RORC ubiquitination by constructing lysine-arginine mutants of potential ubiquitination sites, as indicated by PhosphoSitePlus [16], and detecting their ubiquitination. We identified that RORC ubiquitination was disrupted by its K90R, K277R, and K467R in Th17 cells (Fig. 6L), which were evolutionarily conserved (Fig. 6M), indicating similar modifications in other species.

### LTβR inhibited PRDM1-initiated *Foxp3* transcription

**Fig. 7:**
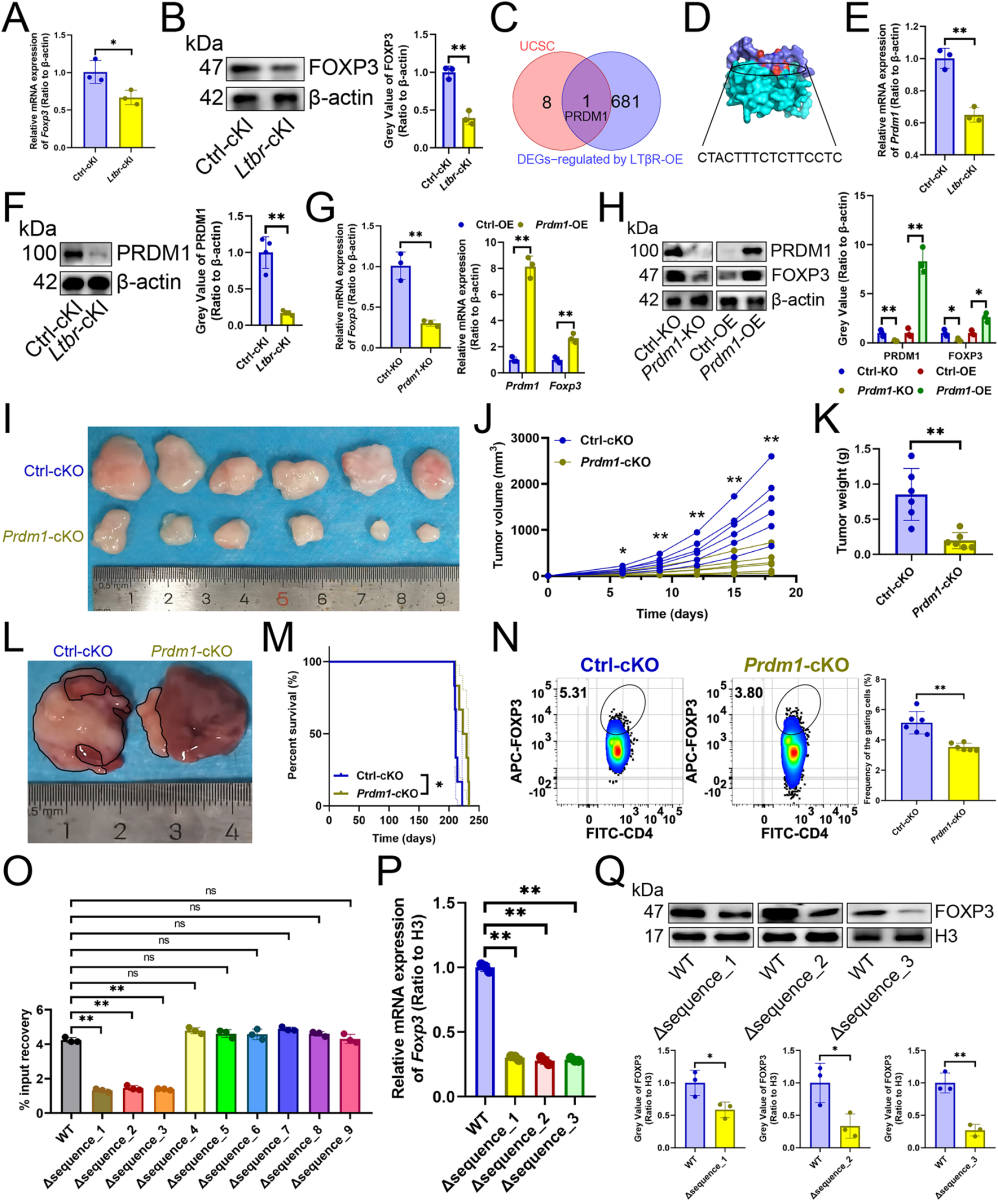
LTβR inhibited PRDM1-initiated *Foxp3* transcription. **A**, **B** Effects of *Ltbr*-cKI in Treg cells on FOXP3 transcript (**A**) and protein (**B**) expression (*n* = 3). **C** A Venn diagram illustrated the overlap between transcription factors predicted by UCSC to bind the *Foxp3* promoter and transcripts regulated by LTβR overexpression in CD4^+^ T cells. **D** A surface plot depicted the docking model and interface residues between PRDM1 protein (sky blue) and the *Foxp3* promoter (cyan). **E, F** The impact of *Ltbr-*cKI in Treg cells on PRDM1 transcript (**E**) and protein (**F**) expression (*n* = 3). **G**, **H** Effects of *Prdm1* knockout or overexpression on FOXP3 transcript (**G**) and protein (**H**) levels in Treg cells (*n* = 3). **I**–**K** *Prdm1*-cKO in Treg cells affecting subcutaneous tumor growth (*n* = 6). **I** Representative. **J** Growth curves. **K** Tumor weight. **L**, **M** *Prdm1*-cKO in Treg cells affecting primary carcinoma growth (*n* = 6). **L** Representative. **M** Survival curves. **N** Flow cytometry revealed how Treg cell-specific *Prdm1*-cKO affected Treg cell infiltration (*n* = 6). **O** The impact of deleting the putative PRDM1 binding sequence within the *Foxp3* promoter, as predicted by JASPAR, on the affinity of PRDM1 to the promoter region was assessed by chromatin immunoprecipitation-quantitative PCR analysis (*n* = 3). **P**, **Q** RT-qPCR (**P**) and immunoblotting (**Q**) analyses demonstrated the impact of deleting sequence_1 (AGATGAGGAAAGTCAGTCTCTTTTTTG), sequence_2 (AAAAAGTGCAAATGAGGGAAAGAGC), and sequence_3 (CTACTTTCTCTTCCTC) on the expression of FOXP3 in Treg cells (*n* = 3). **A**, **B**, **E**–**H**, **J**, **K**, **N**–**Q** represented mean ± SD analyzed by unpaired *t* test, M was analyzed by Log-rank test. *P < 0.05, **P < 0.01. cKI, conditional knock-in; cKO, conditional knockout.

We have found that *Ltbr*-cKI in CD4^+^ T cells reduced Treg cell infiltration (Fig. 1K), so we investigated how LTβR down-regulated *Foxp3* transcription. *Ltbr*-cKI in Treg cells appeared to inhibit FOXP3 transcript or protein levels (Fig. 7A, B). We identified PRDM1 as the only transcription factor up-regulated by LTβR overexpression [9] that potentially initiates *Foxp3* transcription according to UCSC [17] (Figs. 2A, 7C). JASPAR [18] predicted that PRDM1 bound to a series of *Foxp3* promoter sequences, including CTACTTTCTCTTCCTC (Fig. 7D, Table S1). *Ltbr*-cKI in Treg cells reduced PRDM1 expression at both transcript and protein levels (Fig. 7E, F), which in turn PRDM1 positively influenced FOXP3 transcript and protein levels (Fig. 7G, H). In vivo, *Prdm1*-cKO in Treg cells suppressed tumor growth (Fig. 7I–L), improved prognosis (Fig. 7M), and decreased Treg cell infiltration (Fig. 7N). What’s more, deleting the sequence_1 (AGATGAGGAAAGTCAGTCTCTTTTTTG), sequence_2 (AAAAAGTGCAAATGAGGGAAAGAGC), and sequence_3 (CTACTTTCTCTTCCTC) reduced PRDM1’s binding to the *Foxp3* promoter (Fig. 7O) and inhibited FOXP3 transcript and protein expression (Fig. 7P, Q). Hence, we suggested that LTβR improved the immune microenvironment by suppressing the transcription of *Prdm1*, which in turn delayed the transcription of *Foxp3*, a critical factor in Treg cells.

### N-glycosylation of LTβR maintained its stability

**Fig. 8:**
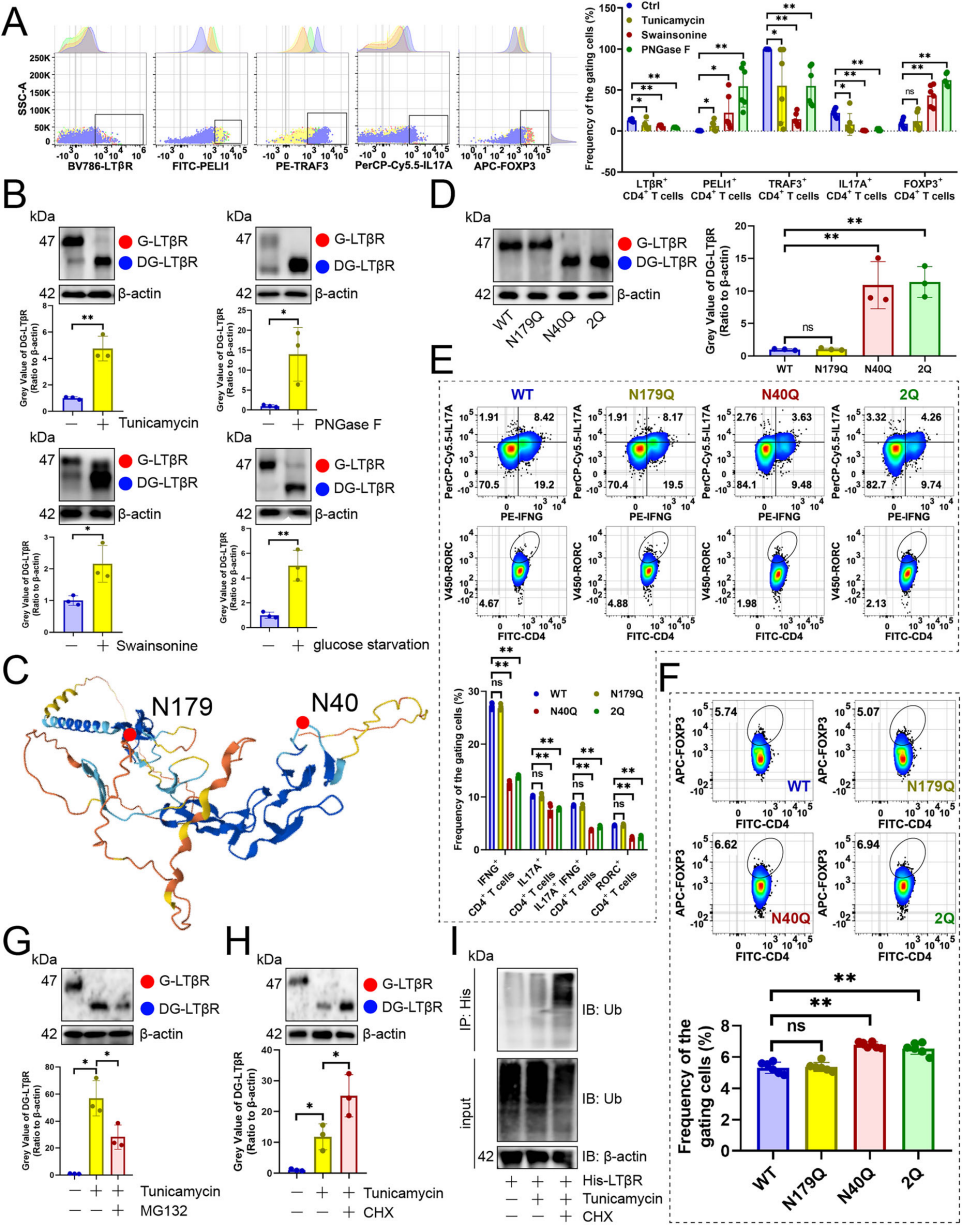
N-glycosylation of LTβR maintained its stability. **A** Flow cytometry analysis of how Tunicamycin, PNGase F, Swainsonine, and glucose starvation affected the protein expression of genes related to the LTβR signaling axis in CD4^+^ T cells (*n* = 6). **B** Effects of Tunicamycin, PNGase F, Swainsonine, and glucose starvation on N-glycosylation of LTβR in CD4^+^ T cells (*n* = 3). **C** A diagram of LTβR protein structure and N-glycosylation sites. **D** Impact of mutating two possible N-glycosylated asparagine residues to glutamine on N-glycosylation of LTβR in CD4^+^ T cells (*n* = 3). **E** Effects of transfecting Th17 cells with constructs where asparagine was mutated to glutamine on Th17 cell abundance and RORC expression in vitro (*n* = 6). **F** The impact of in vitro transfection of Treg cells with two constructs, mutating asparagine to glutamine, on Treg cell levels (*n* = 6). **G**, **H** CD4^+^ T cells pre-stimulated with MG132 (**G**) and Cycloheximide (**H**) were treated with Tunicamycin to evaluate N-glycosylation of LTβR (*n* = 3). **I** CD4^+^ T cells pre-stimulated Cycloheximide were treated with Tunicamycin to assess LTβR ubiquitination (*n* = 3). **A**, **B**, **D**–**H** represented mean ± SD analyzed by unpaired *t* test. *P < 0.05, **P < 0.01.

Since glucose was reported to enhance Th17 cell differentiation [19] while suppressing Treg cell infiltration [20]. Given PhosphoSitePlus [16] showed LTβR undergoes N-glycosylation, we hypothesized that this modification is crucial for up-regulating the Th17/Treg cell ratio. We treated CD4^+^ T cells with three N-glycosylation inhibitors—Tunicamycin, PNGase F, and Swainsonine—along with glucose deprivation. As expected, low glucose reduced LTβR, TRAF3, and IL17A expression while increasing PELI1 and FOXP3 levels (Figs. 8A, S5, S5). As expected, low glucose reduced LTβR’s molecular weight, confirming the higher weight form was N-glycosylated (Fig. 8B). We investigated which asparagine sites in LTβR are N-glycosylated. According to PhosphoSitePlus [16], LTβR has two potential N-glycosylation sites: N179 and N40 (Fig. 8C). Mutating two sites revealed that only N40Q inhibited modification (Fig. 8D). In vitro transfection of CD4^+^ T cells with the N40Q mutant showed inhibition of Th17 cell differentiation (Fig. 8E) and increased Treg cell levels (Fig. 8F). Finally, we explored whether deglycosylated LTβR is prone to ubiquitination-dependent degradation due to the link between N-glycosylation and ubiquitination [21]. We found that deglycosylated LTβR levels decreased with MG132 treatment (Fig. 8G) but increased with Cycloheximide (Fig. 8H). What’s more, immunoprecipitation of LTβR in Tunicamycin-treated CD4^+^ T cells revealed higher ubiquitination than controls, with Cycloheximide enhancing this effect (Fig. 8I). Therefore, the N-glycosylation of LTβR protected it from ubiquitination degradation.

### LTβR improved the immunotherapy efficacy of glycolysis inhibitors

Our earlier single-cell RNA sequencing data [22] showed that HCC cells had the highest glycolysis/gluconeogenesis pathway scores (Fig. S6A). We hypothesized that glycolysis in HCC cells decreases microenvironmental glucose, disrupting the Th17/Treg cell ratio balance maintained by LTβR N-glycosylation. Implanting Hep-53.4 cells which overexpressed *Pkm* or *Glut1* in mice showed that these enzymes hindered Th17 cell infiltration (Fig. S6B, S6C) and boosted Treg cell levels (Fig. S6B, S6D). However, *Ltbr*-cKI in CD4^+^ T cells didn’t counteract this, suggesting LTβR’s role in the Th17/Treg cell ratio balance depended on adequate glucose (Fig. S6C, S6D). To investigate whether the effects of LTβR in preclinical models are applicable to patient HCC and whether it can increase sensitivity to immunotherapy with glycolytic inhibitors, we created patient-derived orthotopic xenograft (PDOX) models and treated them with two FDA-approved glycolysis inhibitors: Alkannin (PKM2 inhibitor) and WZB117 (GLUT1 inhibitor). Additionally, Ark313 [23] was used to specifically overexpress LTβR in human CD4^+^ T cells to test if LTβR improves the drugs’ effectiveness. Both two drugs and LTβR overexpression inhibited HCC growth (Fig. S6E) and increased the Th17/Treg cell ratio (Fig. S6F). Meanwhile, LTβR enhanced Alkannin’s effect on IFNG/RORC promotion and Treg cell suppression, and improved WZB117’s effect on IFNG/RORC promotion (Fig. S6F). Both inhibitors also boosted LTβR’s Th17 cell infiltration.

## Discussion

This study highlighted LTβR’s crucial role in HCC immunity by regulating Th17 and Treg cell differentiation. *Ltbr*-cKI in CD4^+^ T cells promoted Th17 cell levels while reducing Treg cell infiltration. Mechanistically, LTβR indirectly stabilized RORC protein via E3 ubiquitin ligases PELI1 and SMURF1, and inhibited *Foxp3* transcription initiated by transcription factor PRDM1. Moreover, we highlighted the importance of N-glycosylated LTβR in regulating the Th17/Treg cell balance and suggested that LTβR could increase the sensitivity to the glycolysis inhibitors for cancer prevention, which in turn enhanced the therapy of LTβR (Fig. S7).

The Th17/Treg cell ratio imbalance impacts tumor clearance and patient survival, with debates surrounding its role in cancer immunity [24]. In various cancers, Th17 and Treg cells, influenced by different cytokines, either hinder or support tumor growth, largely based on the microenvironment’s inflammatory response [25]. HCC has been reported as an inflammation-suppressive cancer where Th17 cells curb tumor growth by secreting IFNG, counteracting the immunosuppressive role of Tregs [24, 25]. Th17 cells are thought to suppress tumor growth, induce tumor cell apoptosis, and inhibit angiogenesis by releasing cytokines like IFNG and TNFA in HCC [24, 26]. Therefore, balancing the Th17/Treg cell ratio is crucial for effective tumor treatment.

It has been reported that ubiquitination is closely related to Th17 cell differentiation, and regulating the E3 ubiquitin ligases or deubiquitinases involved in RORC ubiquitination is the key to affecting Th17 cell activity and cytokine secretion [27, 28]. For instance, deubiquitinase USP1 activates RORC protein by removing its ubiquitin [27]. RNF157 enhances Th1 cell differentiation and reduces Th17 cell differentiation by modulating HDAC1 ubiquitination, thus curbing experimental autoimmune encephalomyelitis [28]. Our study found that *Ltbr*-cKI in CD4^+^ T cells suppressed E3 ubiquitin ligase *Peli1* transcription and stabilized TRAF3 protein. Surprisingly, despite being an E3 ubiquitin ligase, TRAF3 slowed RORC degradation by competing with RORC for SMURF1’s HECT domain, protecting RORC from SMURF1. Here, we suggested that LTβR triggered two ubiquitination processes: one degraded its substrate, while the other inhibited degradation of each other by binding to the common E3 ubiquitin ligase, thus preserving RORC activity and Th17 cell infiltration.

Single cancer therapies have severe side effects, making combination therapy an ideal treatment approach [29]. T cell proliferation and differentiation are crucial for anti-tumor activity. Research shows that T cell growth, differentiation, and lifespan are influenced by metabolic processes, impacting tumor progression [30]. Therefore, regulating metabolic pathways offers a promising approach for cancer treatment. For instance, 3-bromopyruvate and sodium citrate hinder tumor growth by targeting HK2 and phospho-PFK [31]. Our findings showed that targeting T cells with high LTβR expression could work synergistically with glycolysis inhibitors. LTβR boosted the immune response to Alkannin and WZB117, increased the Th17 cell levels, and reduced Treg cell abundance, while both inhibitors also enhanced LTβR-mediated IL17A expression. Our data suggested that metabolic process nodes could regulate immune cell differentiation. Inhibiting aerobic glycolysis in tumor cells may boost sugar availability in the microenvironment, offering a novel tumor treatment strategy.

We recognized the potential limitations of this study. First, the genetic background of the PDOX depends on the patient’s sensitivity to glycolytic inhibitors, so the assessment of the TME of patients with insensitivity is biased. Second, cKO or cKI of *Ltbr*, *Peli1*, *Traf3*, and *Prdm1* theoretically poses genotoxicity issues. Finally, for clinical applications, it is important to recognize that if gene-edited T cells exhibit strong proliferation potency, the accumulation of T cells may greatly increase the risk of T cell lymphoma and cytokine storm. This can be addressed in dose-escalation studies. Considering these uncertainties, preliminary clinical trials of this strategy should evaluate LTβR-high-expressing T cell products at relatively low concentrations prior to the introduction of glycolytic inhibitors. We anticipated that this method will not only enhance T cell proliferation and anti-tumor efficacy but also reduce treatment-related toxicity through the adjustment of T cell infusion doses and the concomitant use of glycolysis inhibitors.

In summary, our study demonstrated that N-glycosylated LTβR inhibited the progression of HCC and increased sensitivity to the anticancer effects of glycolysis inhibitors by elevating the Th17/Treg cell ratio.

## Supplementary information

Supplementary Information

Original western blots

## Data Availability

The data that support the findings of this study are deposited in PRJNA1131099 (single-cell RNA sequencing), PRJCA034208 (Mass cytometry), PRJCA029034 (Mass spectrometry for RORC binding proteins), PXD062760 (Mass spectrometry for PELI1 or TRAF3 binding proteins). The dataset of the public database used in this study is GSE193736. No new codes were generated in this study.
