# Supplementary material for: N-glycosylated LTβR increases the Th17/Treg cell ratio in liver cancer by blocking RORC ubiquitination and FOXP3 transcription
Source: Cell Death Dis. 2025 May 28;16(1):421. doi: 10.1038/s41419-025-07738-2 (PMC12120105; doi:10.1038/s41419-025-07738-2)
Supplement: Supplementary file 1 — Supplementary Information [file 41419_2025_7738_MOESM1_ESM.docx]

***Supplementary information for***

**N-glycosylated LTβR increases the Th17/Treg cell ratio in liver cancer by blocking RORC ubiquitination and FOXP3 transcription**

**Table of contents**

**Supplementary Materials and Methods**2

**Supplementary Figures**20

**Supplementary Table**28

**Supplementary References**28

**Supplementary Materials and Methods**

**Study design**

The objective of this study was to elucidate the role of LTβR in modulating the Th17/Treg cell ratio balance and to assess the functional impact of LTβR-overexpressing CD4^+^ T cells in conjunction with glycolytic inhibitors on antitumor immunity. Details regarding the number of biological replicates, statistical methods employed, and *P* values were provided in the legends. Sample sizes were not predetermined using statistical methods; instead, they were estimated based on preliminary experimental data. All in vitro functional experiments were conducted a minimum of three times. Animal experiments were conducted at least five times, with mice randomly assigned to treatment groups by cage. This study did not set any special inclusion or exclusion criteria for cytological experiments or animal experiments. During allocation, experimental conduct, result evaluation and data analysis, we ensured that the experimenters in each link were independent and randomization and did not interfere with each other. Investigators were blinded to group assignments during both the experimental procedures and outcome assessments. All ethical guidelines and regulations were strictly adhered to. The residual tumor tissues, identified through pathological diagnosis, were utilized in this study. Patients/participants have provided written informed consent to participate in this study. Given the post-hoc nature of this study, the analyses presented should be considered exploratory.

**T cell treatment strategies**

Various mouse HCC models were constructed (**Mouse model**). Following the sacrifice of mice, primary HCC tissues were dissociated into single cell suspensions (**Isolation of tumor tissue**). Subsequently, live T cells were isolated through flow cytometry (**Flow cytometry analysis or sorting**). Cytokines were employed to induce the differentiation of T cells into either Th17 or Treg cells (**Cell culture and treatment**). Cell transfection, immunoblotting, immunoprecipitation, and RT-qPCR were then employed.

**ATAC-seq analysis**

Bowtie2(1) was employed to align the single-ended reads with the human genome version hg38(2). Subsequently, SAMtools(3) was utilized to filter out low-quality mappings, followed by sorting and indexing of the filtered BAM files. Duplicate reads were removed using Picard. Peak calling was performed using MACS3(4). To construct a union peak, BEDTools(5) was initially used to intersect the biological replicate narrow peak file pairs, retaining only those peaks present in both replicates. BEDTools(5) merge was then applied to consolidate the biological duplications for each shared peak. Subsequently, each peak file was merged, combining peaks that overlapped by at least 1 bp. The overlapping reads were directly extracted from the BAM file, which provided alignment for each sample, resulting in a matrix where each column represents a biological replicate. In addition to the per-gene ATAC matrix, a per-gene ATAC matrix was constructed as follows: the total ATAC read value for each gene was assigned as the sum of the normalized read values from the per-gene ATAC matrix for all peaks located within 3 kb of the gene’s start or end coordinates. We imported the ATAC-seq matrices into R software and employed DESeq2(6) for the analysis of gene and peak enrichment. To generate read heap tracks at specific genomic loci, we utilized samtools merge to aggregate de-duplicated reads from biological replicates. Subsequently, we used the bamCoverage function in deepTools(7) to convert these pooled BAM files into bigWig files, setting the scaleFactor according to the relative abundance of transcription start sites found in pooled biological replicates compared to other sample aggregations. Using the bigWig file, we visualized the read heap with pyGenomeTracks(8). Finally, we conducted *k*-means clustering on ATAC-seq peaks proximal to genes exhibiting increased chromatin accessibility. Initially, we applied DESeq2(6) to the per-gene ATAC-seq matrix to identify genes with a log_2_ (fold change) > 1.5 and an adjusted *P*-value < 0.05 in two comparative analyses. Following the identification of these genes, we isolated all accessibility peaks located within 3 kb of the genome in each peak ATAC-seq matrix. This subset of peaks from each ATAC-seq matrix served as the input for clustering. Subsequently, using deepTools(7), we performed *k*-means clustering on the read windows of this subset of ATAC-seq peaks.

**Construction**

The target genes were PCR-amplified from cDNA, cloned into the pDNA3.1 vector (V79020, Addgene, MA, USA), and tagged with 6×His, HA, or Myc at the C-terminus.

**Cell culture and treatment**

HEK293T (300189, Cytion, Germany) and Hep-53.4 (400200, Cytion) cells were grown in DMEM (10569010, Gibco, NY, USA) with 10% FBS (10099158, Gibco) and 1% Pen-Strep (HY-K1006, MedChemExpress, NJ, USA), while mouse primary T cells were cultured in T Cell Expansion SFM (A1048501, Gibco) with the same supplements. MycAway™ Plus-Color One-Step Mycoplasma Detection Kit (40612ES25, Yeasen, China) was used to test for mycoplasma contamination every two weeks. All cells were identified by Short Tandem Repeat to ensure that the cell lines were accurate and uncontaminated (provided by Beijing Qingke Biotechnology Co., LTD, China). All cultures were maintained at 37°C with 5% CO_2_. Specific cell stimulation programs were as follows:

**(1)** To ensure the proliferation of T cells, Dynabeads Mouse T-Activator CD3/CD28 (11456D, Gibco) was used to activate them.

**(2)** T cells were cultured in RPMI 1640 Medium, no glucose (11879020, Gibco) with the same supplements for glucose starvation. The drug or small molecule compound treatment scheme was as follows: T cells were treated with Cycloheximide (500 nM, HY-12320, MedChemExpress), MG132 (40 μM, HY-13259, MedChemExpress), PNGase F (500U, HY-P2929, MedChemExpress), Swainsonine (40 μM, HY-N6722, MedChemExpress), and Tunicamycin (2 μg/ml, HY-A0098, MedChemExpress) for 48 h, respectively.

**(3)** In vitro differentiation of Th17 and Treg cells. For Treg cell differentiation, the culture medium was supplemented with 15 ng/ml mouse TGFB1 (763102, BioLegend, CA, USA), 30 U/ml mouse IL2 (714604, BioLegend), 5000 ng/ml mouse IFNG (714006, BioLegend), and 5000 ng/ml mouse IL4 (574302, BioLegend). For Th17 cell differentiation, the medium contained 20 ng/ml mouse IL6 (575702, BioLegend), 3 ng/ml mouse TGFB1, 5000 ng/ml mouse IFNG, and 5000 ng/mL mouse IL4. The cells were incubated at 37°C with 5% CO_2_ for a duration of 4 to 5 days. The differentiation outcomes were assessed using flow cytometry analysis.

**Mouse model**

All animal experiments followed protocols approved by Fujian Medical University’s Ethics Committee (IACUC FJMU 2024-0375). 12-week-old female C57BL/6J mice (~23 g) (GemPharmatech, China) were kept in ventilated cages in a pathogen-free environment at room temperature (RT) and 50%-60% humidity. Six mice were randomly assigned per group. Specific operation strategy:

**(1)** Construction of subcutaneous tumor model: Hep-53.4 cells (5 × 10^6^) were suspended in 100 μl DPBS (14190144, Gibco) and injected subcutaneously into mice. Tumor formation was observed when the control group’s average tumor diameter reached at least 2 mm. When a mouse’s tumor reached 2 cm in diameter, it was euthanized with CO_2_, and the tumor was removed, photographed, and weighed. Tumor volume was calculated using the formula: (long axis × wide axis²) × 0.5.

**(2)** Construction of primary HCC model: Briefly, a single dose of DEN (25 mg/kg, HY-N7434, MedChemExpress) was injected into 15-day-old female C57BL/6J mice intraperitoneally (*i.p.*) to initiate tumor formation. At 4 weeks of age, CCl_4_ (0.5 mL/kg, HY-Y0298, MedChemExpress) was injected *i. p*. Twice a week with CCl_4_ for an additional 16 weeks. After approximately 25 weeks, HCC was formed in the liver, and confirmation was obtained by bimanual palpation and dissection. Animal ethics stipulated that if an animal becomes listless and loses its appetite, it should be euthanized with CO_2_.

**(3)** Construction of patient-derived orthotopic xenograft (PDOX) model: HCC samples were obtained at the Fujian Medical University Union Hospital. This study was approved by the Ethics Committee of Fujian Medical University Union Hospital (approval number 2021KJCX008). Briefly, mechanically minced pieces of fresh HCC tissue were plated on bottles at 37°C in Human Liver Organoid Culture Medium (abs9529, Absin, China) for up to 2 weeks. Organoids with a diameter of 300 µm were made with DPBS at a concentration of 2×10^7^/ml, 1 × 10^6^ cells per mouse, and an injection volume of 50 μl. 12-week-old female huHSC-NCG mice (~23 g) (GemPharmatech) were anesthetized with 0.8% Pentobarbital Sodium (60 mg/kg, P3761, Merck, Germany) by *i. p*. After anesthesia, the mice were fixed on the operating board, and the surgical site was disinfected. A longitudinal incision was made about 1 cm below the xiphoid process of mice to open the abdominal cavity, and the left lobe of the liver was gently exposed with a sterile cotton swab. Fix the liver lobe with a cotton swab in the left hand, insert a 1 ml micro-syringe along the liver surface at 15-30° with the right hand, penetrate the liver of about 0.5 cm, inject the cell suspension slowly, withdraw the needle slowly, and use a sterile cotton swab to lightly press the injection site to stop bleeding. The liver was then carefully placed back into the abdominal cavity, the abdomen was sutured layer by layer, and the incision was sterilized. Place the mice on an electric blanket until the mice wake up, then return them to their cages and observe the changes in the mice’s vital signs and body weight. Mice (passage 1) were maintained and sacrificed when behavioral abnormalities (collapse, hyperactivity) and weight loss occurred. Organoids (passage 1) were further prepared from minced xenograft livers in the same manner as patient tissues and implanted for several generations. The PDOX model was established at passage 3, when tumor phenotype tended to stabilize.

**(4)** Construction of *Peli1*-, *Traf3*-, or *Prdm1*-cKO mice: To create a *Peli1*-cKO mouse model (T063523, GemPharmatech), Exon 3 of *Peli1* transcript was targeted as the flox region. For a *Traf3*-cKO mouse model (T016527, GemPharmatech), Exon 4-Exon 6 of *Traf3* were selected. According to the structure of *Prdm1* gene, Exon 3-Exon 4 of *Prdm1* transcript were recommended as the cKO region, and thus a *Prdm1*-cKO mouse model (T016016, GemPharmatech) was constructed. Knocking out these regions would induce the frameshift mutation, leading to premature translation termination and protein mutations. Fertilized eggs were implanted into female C57BL/6J mice to produce positive F0 mice. To achieve Th17 cell-specific KO, floxed *Peli1* or *Traf3* mice were bred with *Il17a-Cre* transgenic mice (NM-KI-200137, Shanghai Model Organisms Center, Inc., China). To obtain Treg cell-specific KO, floxed *Prdm1* mice were bred with *Foxp3-Cre* transgenic mice (T010853, GemPharmatech). For animal studies with *Peli1*^flox/flox^*Il17a-Cre* (*Peli1*-cKO), *Traf3*^flox/flox^*Il17a-Cre* (*Traf3*-cKO), or *Prdm1*^flox/flox^*Foxp3-Cre* (*Prdm1*-cKO) mice, littermates with *Peli1*^flox/flox^, *Traf3*^flox/flox^, or *Prdm1*^flox/flox^ as control were used. Deletion of the floxed *Peli1* allele and wild-type (WT) *Peli1* Exon 3 was detected by PCR using the following primers: Floxed*-Peli1* (F-TCTGAGGCGGAAAGAACCAG, R-ATCTCCTTGCCAGTGGTC), WT*-Peli1* (F-TGGGGTCCAGATAAGAACAA, R-TACTTAAATGCGTTTGACTG). Deletion of the floxed *Traf3* allele and WT *Traf3* Exon 4-Exon 6 using the following primers: 5’ arm*-Traf3* (F-AGGCACTGTGCTTGACACTCAG, R-CAGCAATTTCAACCTGGGTC), 3’ arm*-Traf3* (F-GCATCGCATTGTCTGAGTAGGTG, R-CCTGAACAGCACTCATTTCCTG). Deletion of the floxed *Prdm1* allele and WT *Prdm1* Exon 3-Exon 4 using the following primers: 5’ arm*-Prdm1* (F-CCCTTTCCTGTCTTAAGAATGCC, R-TTTCACAGTGGCAGGTGAGC), 3’ arm*-Prdm1* (F-CATCGCATTGTCTGAGTAGGTG, R-TGACAGTGGCTCAAGGCTTTC). Primers that produce Th17 cell-specific KO: common-Th17 (CAAGTGCACCCAGCACCAGCTGATC), WT reverse-Th17 (CTTAGTGGGTTAGTTTCATCACAGC), mutant reverse-Th17 (AGTCCCTCACATCCTCAGGTT). Primers that produce Treg cell-specific KO: 5’ connector-Treg (F-AGCAAGTGAGGTGCTGGACATG, R-CTGCACACAGACAGGAGCATCTTC), 3’ connector-Treg (F-TGGTGGCTGGACCAATGTGAAC, R-ATAGCCAACCTTTGTTCATGGC).

**(5)** Construction of *Ltbr*- or *Peli1-*cKI mice: Using CRISPR/Cas9 technology, a CAG-tdTomato-polyA expression frame was inserted into the *Rosa26* gene locus by homologous recombination. A homologous recombination vector containing a 5’ homology arm, a tdTomato expression frame, and a 3’ homology arm was constructed by In-Fusion cloning. Cas9 mRNA, gRNA, and donor vector were microinjected into fertilized eggs of female C57BL/6J mice to obtain mice with loxp sites and mate with *Cd4-Cre* (T055135, GemPharmatech) or *Il17a-Cre* mice. Primers that produce CD4^+^ T cell-specific KI: 5’ arm-CD4^+^ T (F-GCCCAAGGAATACCTGAAGACTG, RTGCCAATGTGGATCAGCATTC), 3’ arm-CD4^+^ T (F-GCCCAAGGAATACCTGAAGACTG, R-GATCTTCTTCTGGGAACTCTCGC). Primers for *Ltbr*- or *Peli1*-cKI identification: WT primers-used to identify the presence of WT alleles and Cas9 activity (F-ggagtgttgcaatacctttctgggagttc, R-tgtccctccaattttacacctgttcaattc), Transgene primers-used to identify whether the Donor integration into the genome (F-ctgtagggcgcagtagtccag, R-gctagaactagtggatctcgagcc), 5’ junction primers-used to identify whether the Donor inserted into the *Rosa26* locus (Out-F-ctgcccgagcggaaacgccactgac, SA-R-cctggactactgcgccctacaga), 3’ junction primers-used to identify whether the Donor inserted into the *Rosa26* locus (3’ Insert-F-ggctcgagatccactagttctagc, Out-R-ccattctcagtggctcaacaacac), Cre primers-used to determine whether the floxed cassette is removed by the Cre (pCAG-F-gcaacgtgctggttattgtg, SK-R-gccgctctagaactagtggatcc). The amplified fragments of *Ltbr* and *Peli1* were 1396 and 1405 bp, respectively.

**(6)** Ark313-LTβR-overexpression (OE) production and tail vein infusion: Ark313 is a synthetic AAV-6 that exhibits high transduction efficiency in human T cells(9). HEK283T cells were co-transfected with 11 μg AAV-6 Packaging System (VPK-406, Cell Biolabs, CA, USA), 8 μg AAV-6 Rep-Cap Plasmid (VPK-426, Cell Biolabs), and 6 μg Ark313(9)-LTβR-OE plasmid using the AAV-MAX System (A51217, Gibco) for 72 h. Transfected cells were collected in Viral Production Medium (A4817901, Gibco), lysed by three cycles of rapid freeze/thaw, and then incubated with 25 IU/ml Benzonase (E1014, Merck) for 1 h at 37°C. The high-pressure injection technique via tail vein was performed. Briefly, PDOX model was injected with 1 ml of saline containing Ark313-LTβR-OE (5 μg) within 5-10 s via the tail vein.

**(7)** Small molecule compound treatment: The small molecule compound injection protocol involved administering Alkannin (10 mg/kg, HY-119874, MedChemExpress) and WZB117 (10 mg/kg, HY-19331, MedChemExpress) into the tail vein 20 days before the mice were sacrificed, and then every three days for a total of five doses.

**Cell transfection**

For electrotransfection: T cells were suspended in Opti-MEM™ I Reduced Serum Medium (31985062, Gibco) at a concentration of 10^7^ cells/ml. Plasmid DNAs were added into the suspension to achieve a final concentration of 10 mg/ml. The pulses were generated by using a NxT Electroporation System (NEON18SK, Thermo Scientific, CA, USA). After electrotransfection, samples were incubated at 37℃ for 10 min to promote endocytosis.

For gene knockout, CRISPR constructs and the packaging plasmid pMD2.G (12259, Addgene)/psPAX (12260, Addgene) were co-transfected into HEK293T cells with Lipofectamine™ CRISPRMAX (CMAX00008, Invitrogen, CA, USA), then packaged into lentivirus. After 48 h, the medium was collected to infect T cells. CRISPR target sequence: *Otud1* (Left TALEN Sequence-TCCATGGCCATATCCCTG, Right TALEN Sequence-AAGAGCATGCATTTTGTT), *Peli1* (TTTATCTCGAGCCCAGACGG), *Prdm1* (GGGTACTTCTGTTCAAGCCG), *Rorc* (AGTAGGCCACATTACACTGC), *Smurf1* (ACTCGATAACCATCAGTGTG), *Traf3* (AAAAGACTTGCGGGATCACG), and *Usp18* (ACGGCACGTTGTATTTCTGC).

**Chromatin Immunoprecipitation-qPCR (ChIP-qPCR)**

We conducted the ChIP assay using SimpleChIP® Plus Sonication Chromatin IP Kit (56383, Cell Signaling Technology, MA, USA). Cells were cross-linked with 1% formaldehyde (28908, Thermo Scientific) for 10 min and stopped with 125 mM glycine at RT. After two DPBS washes, cells were collected in DPBS with 1% PMSF (36978, Thermo Scientific) and centrifuged at high speed for 5 min. DNA was then resuspended in dilution buffer, 4 μg of anti-PRDM1 antibody (MA1-16874, Invitrogen) was added, and the mixture was incubated at 4°C for 2 h. Magnetic beads were incubated at 4 °C for 1 h. The DNA was sequentially washed for 10 min with buffer I, buffer II, buffer III, and Tris-EDTA buffer (twice). The beads were then shaken vigorously in the elution buffer for 15 min at RT. The supernatant was collected and incubated overnight at 65°C. DNA was purified, and the target fragment was amplified via PCR. The upstream and downstream primers used to detect DNA fragments were Sequence_1 (F-CCAAGCCATCAGTTCCAGTCTTG, R-GGCTATCTTCTGAGCACCTCCC), Sequence_2 (F-GTGCTCAGAAGATAGCCGAA, R-AAACAATTCCCACACTCCTTTGC), Sequence_3 (F-AGGAGTGTGGGAATTGTTTACT, R-ACCTCTTTGCAAGACTGGCT), Sequence_4 (F-AACAAGCACTCTGGCACTGA, R-TAATGCAGCCAGCATCACCA), Sequence_5 (F-AGGGCACTCAGCACAAACAT, R-CAGAAGGCAAGAGGCATGGA), Sequence_6 (F-TCCATGCCTCTTGCCTTCTG, R-CAGAGGCTTCCTTCTGCTCC), Sequence_7 (F-GAGCAGAAGGAAGCCTCTGG, R-AGGATCGCTGGGTTGAACAG), Sequence_8 (F-CAGCGATCCTCCAACGTCTC, R-GTCAAATCACAAAGGCATCCCG), and Sequence_9 (F- GGGATGCCTTTGTGATTTGACTTA, R-ACAGGGCTCATGAGAAACCAC), respectively.

**Cytometry by time-of-flight (CyTOF)**

**(1)** Antibody labeling: Antibodies were conjugated to isotopically enriched lanthanide metals using the Maxpar X8 Antibody Labeling Kit (NC1648790, Standard BioTools, CA, USA). The labeled antibodies were stored in DPBS supplemented with 1% glycerol (1295731, Merck), 0.05% BSA (AM2616, Invitrogen), and 0.05% sodium azide (S2002, Merck) at 4℃. Antibodies used in this study: anti-BATF (ab237038, Abcam, UK), anti-BCL6 (PA5-95591, Invitrogen), anti-CD3E (14-0032-82, Invitrogen), anti-CD4 (14-0041-82, Invitrogen), anti-CD8A (14-0808-82, Invitrogen), anti-CD25 (14-0251-86, Invitrogen), anti-CD27 (14-0271-82, Invitrogen), anti-CD45 (14-0451-82, Invitrogen), anti-CD127 (14-1271-82, Invitrogen), anti-CD196 (PA5-94947, Invitrogen), anti-CD278 (14-9949-82, Invitrogen), anti-CXCR5 (MA5-41258, Invitrogen), anti-FOXP3 (PA1-46126, Invitrogen), anti-GATA3 (A700-121, Invitrogen), anti-IFNG (MM700, Invitrogen), anti-IL3 (ab249402, Abcam), anti-IL4 (14-7042-85, Invitrogen), anti-IL5 (14-7052-81, Invitrogen), anti-IL10 (ARC9102, Invitrogen), anti-IL12 (MA5-52960, Invitrogen), anti-IL13 (ab242663, Abcam), anti-IL17A (PA5-46947, Invitrogen), anti-IL21 (ab282737, Abcam), anti-IL22 (PA1-21356, Invitrogen), anti-IRF4 (ab315395, Abcam), anti-MKI67 (MA5-14520, Invitrogen), anti-RORC (MA5-16227, Invitrogen), anti-STAT1 (MA5-41111, Invitrogen), anti-STAT3 (PA5-18562, Invitrogen), anti-STAT6 (ab215995, Abcam), anti-TBX21 (PA5-109245, Invitrogen), anti-TCRβ (14-5961-82, Invitrogen), anti-TCRγδ (14-5711-82, Invitrogen), anti-TGFB (MA1-21595, Invitrogen), and anti-TNFA (PA1-40281, Invitrogen) antibodies.

**(2)** Cell staining: Collect single cell suspension, add 1 μl of Cisplatin (201064, Standard BioTools) with a final concentration of 5 μM to distinguish live cells from dead cells, incubate at 37℃ for 5 min, and then add five times the volume of Cell Staining Buffer (201068, Standard BioTools) to terminate the labeling reaction. Centrifuge at 300 × g for 5 min, discard the supernatant, and resuspend the cells in Cell Staining Buffer. To detect cytokine expression, stimulate the cells with Cell Stimulation Cocktail (plus protein transport inhibitors) (00-4975-93, eBioscience, CA, USA) for 6 h. After stimulation, the cells were centrifuged at 300 × g for 5 min and diluted to 1 ml with Cell Staining Buffer to terminate the stimulation. Nonspecific signals were blocked by adding 5 μl Fc Receptor Binding Inhibitor Polyclonal Antibody (14-9161-73, eBioscience), followed by staining with pre-mixed surface antibodies at 4℃ for 30 min. After washing the cells twice with Cell Staining Buffer, the cells were fixed in 200 μl of Fix and Perm Buffer (201067, Standard BioTools) containing 250 nM Intercalator-Ir (201192A, Standard BioTools) at 4℃ overnight. The cells were washed twice with Cell Staining Buffer and stained with premixed intracellular antibodies at 4℃ for 30 min. The cells were washed twice with Nuclear Antigen Staining Buffer (201063, Standard BioTools), resuspended in Cell Acquisition Solution Plus for CyTOF XT (201244, Standard BioTools), and then mixed with 20% EQ Four Element Calibration Beads (201078, Standard BioTools). Data were acquired using a CyTOF XT (Standard BioTools) and saved as fcs files.

**(3)** Preprocessing of mass cytometry data: Raw data were normalized using the MATLAB version of the Normalizer tool(10). Cells were assigned by manually gating on Event length and DNA (^191^Ir and ^193^Ir) channels, followed by the dead cell discrimination analyzing ^195^Pt expression using a FlowJo (BD Biosciences, NJ, USA). Doublets were excluded using Gaussian discrimination channels. Next, data were concatenated and de-barcoded using Boolean gating. The normalized data containing living cells from every individual sample were manually exported from FlowJo and imported into RStudio (RStudio, MA, USA) using the “flowCore”(11) and “flowWorkspaceData”(12). Before automated high-dimensional data analysis, the mass cytometry data were transformed with a cofactor in the range of 5 and 60 using an inverse hyperbolic sine function(13). Live and single cells were exported and imported into RStudio. Before automated high-dimensional data analysis, data were transformed using an inverse hyperbolic sine function with a cofactor in the range of between 300 and 600. Additionally, all data were normalized between 0 and 1 to match the 99-999^th^ percentile of the combined samples in each batch.

**(4)** Automated subset identification: To identify T cell subsets accurately, we first performed step 1 of FlowSOM clustering on the preprocessed and combined mass cytometry dataset to generate a starting point of 100 nodes(14). The respective k-value was manually chosen (in the range of between 20 and 30); identified subsets were annotated and merged based on a similarity of antigen expression to uphold the biological relevance of the dataset. Manually annotated subsets were used to calculate the relative frequencies of T cell subsets. Heatmaps showed the median expression levels of all markers for each merged subset and were plotted using the “pheatmap”. From mass cytometry datasets, we pre-selected major subsets and performed additional FlowSOM(14) analysis to identify smaller cell subsets. We calculated the median antigen expression among selected cell types of the mass cytometry batch using the “dplyr”. For data visualization, we applied dimensionality reduction analysis. For a complex overview of the immune compartment, we used t-SNE(15).

**Flow cytometry analysis or sorting**

To validate surface marker expression, the cells were directly stained with the indicated fluorochrome-conjugated antibodies for 30 min in Cell Staining Buffer (00-4222-26, eBioscience) at 4°C and analyzed by flow cytometry. For intracellular staining, the cells were permeabilized, fixed and stained by using Intracellular Fixation & Permeabilization Buffer Set (88-8824-00, eBioscience). For intracellular cytokine detection, the cells were stimulated with Leukocyte Activation Cocktail (550583, BD biosciences) at 37°C for 6 h. The cells were then washed in Cell Staining Buffer three times, resuspended in Cell Staining Buffer and analyzed by flow cytometry. Samples were acquired and recorded in a FACSCelesta™ Flow Cytometer (BD Biosciences) and data were analyzed with a FlowJo software. During cell sorting, the concentration was maintained at 1 × 10^7^ cells/ml, with a pressure of 60 psi, a 70 μm nozzle, and a maximum rate of 20,000 events/s. Primary antibodies used in this study: anti-human-CD3E-APC-eFluor780 (47-0037-42, Invitrogen), anti-human-CD4-FITC (11-0041-82, Invitrogen), anti-human-CD45-eFluor506 (69-0459-42, Invitrogen), anti-human-FOXP3-APC (17-4776-42, Invitrogen), anti-human-IFNG-PE (12-7311-82, Invitrogen), anti-human-IL17A-PerCP-Cy5.5 (45-7179-42, Invitrogen), anti-mouse-CD3E-BV786 (417-0031-82, Invitrogen), anti-mouse-CD3E-eFluor450 (48-0031-82, Invitrogen), anti-mouse-CD4-eFluor506 (69-0042-82, Invitrogen), anti-mouse-CD4-FITC (11-0041-82, Invitrogen), anti-mouse-CD45-BV510 (567800, BD Biosciences), anti-mouse-FOXP3-APC (17-5773-82, Invitrogen), anti-mouse-IFNG-PE (12-7311-82, Invitrogen), anti-mouse-IL17A-APC (17-7177-81, Invitrogen), anti-mouse-IL17A-PerCP-Cy5.5 (45-7177-82, Invitrogen), anti-mouse-LTβR (14-5671-82, Invitrogen), anti-mouse-PELI1 (PA5-34465, Invitrogen), anti-mouse-TRAF3 (ab217033, Abcam), and anti-RORC (MA5-16227, Invitrogen) antibodies. Secondary antibodies used in this study: Goat anti-Rat IgG (H+L) Secondary Antibody-FITC (A18866, Invitrogen), Goat anti-Rat IgG (H+L) Secondary Antibody-PE (PA1-29628, Invitrogen), Rat anti-Mouse IgG1 Secondary Antibody-eFluor 450 (48-4015-82, Invitrogen), and Rat anti-Mouse IgG1 Secondary Antibody-Super Bright 780 (78-4015-82, Invitrogen). Viability dye used in this study: Fixable Viability Stain 575V (565694, BD Biosciences).

**Immunoblotting and immunoprecipitation**

The cells were washed with DPBS, lysed using Novex Tricine SDS Sample Buffer (LC1676, Invitrogen) with 1% PMSF (36978, Thermo Scientific), boiled for 20 min, and analyzed by sodium dodecyl sulfate–polyacrylamide gel electrophoresis. For immunoprecipitation, lysates were incubated with primary antibodies for 1 h at 4°C, then with Protein A/G Magnetic Beads (88802, Thermo Scientific) for 2 h at 4°C. The complexes were washed and subjected to electrophoresis. Primary antibodies used in this study: anti-Flag Tag (MA1-91878, Invitrogen), anti-FOXP3 (PA1-46126, Invitrogen), anti-GFP (MA5-15256, Invitrogen), anti-HA Tag (26183, Invitrogen), anti-Histone H3 (PA5-16183, Invitrogen), anti-LTβR (14-5671-82, Invitrogen), anti-Myc Tag (PA1-981, Invitrogen), anti-OTUD1 (ab122481, Abcam), anti-PELI1 (PA5-34465, Invitrogen), anti-PRDM1 (MA1-16874, Invitrogen), anti-SMURF1 (PA5-11943, Invitrogen), anti-TRAF3 (ab36988, Abcam), anti-RORC (MA5-16227, Invitrogen), anti-STAT3 (PA5-18562, Invitrogen), anti-Ubiquitin (ab134953, Abcam), anti-USP18 (PA5-110555, Invitrogen), and anti-β-actin (MA1-140, Invitrogen) antibodies. Secondary antibodies used in this study: Goat anti-Mouse IgG (H+L) Secondary Antibody-HRP (31430, Invitrogen) and Goat anti-Rabbit IgG (H+L) Secondary Antibody-HRP (31460, Invitrogen).

**Isolation of tumor tissue**

The primary HCC tissue was diced into 1-3 mm pieces and digested with Tissue Dissociation Solution (HY-K6012, MedChemExpress) at 37°C for 30 min. EDTA (10 mM, HY-Y0682, MedChemExpress) was added to stop the reaction. The sample was homogenized using a 23 G needle, strained through a 70 mm filter, and centrifuged at 400 × g for 8 min. Then, it was centrifuged at 900 × g for 30 min in 30% Percoll (40501ES60, Yeasen) to isolate the middle white cell layer. The cells were washed twice with DPBS for subsequent analysis.

**Mass spectrometry**

Protein bound to PELI1 or TRAF3 was isolated with anti-PELI1 (12053-1-AP, Proteintech, China) or anti-TRAF3 (ab36988, Abcam) antibodies and sent to 10K Genomics (Shanghai, China) for analysis.

**Metabolic activity assessment**

Metabolic activity was evaluated by quantifying pathways with ssGSEA and comparing cell metabolic profiles using scMetabolism(16). Metabolite abundance was assessed with scFEA from single-cell RNA-sequencing data(17).

**Molecular docking analysis of DNA with protein**

3dRNA(18) was used to construct the 3D structure of DNA. Five models were constructed for each structure. The energy was minimized using the minimized module of Discovery Studio software, and the structure with the lowest energy was selected as the optimal structure. The protein structure was constructed using I-TASSER(19) and subsequently visualized using PyMol, and Mgtools was performed to add/remove hydrogens, calculate charges, and merge nonpolar hydrogens. The ligands were docked to the receptors using AutoDock Vina(20), and the higher scoring conformations were selected and visualized using PyMol and LigPlus(21).

**Molecular docking analysis of protein with protein**

HDOCK(9) was used as a molecular docking program to analyze the interaction between proteins. PyMol was used to separate the original ligand and protein structure, dehydrate to remove organic matter, and then the Prepare module of Discovery Studio was used to prepare the protein, such as hydrogenation and protonation. LigPlus(21) was performed to analyze the forces between two proteins in two dimensions. The protein interaction interface was analyzed using the Analysis Interface module of Discovery Studio, and PyMol was applied to draw the interacting amino acid residues between two proteins.

**RT-qPCR**

Total RNA was extracted using the FastPure Cell/Tissue Total RNA Isolation Kit V2 (RC112-01, Vazyme, China), then reverse transcribed with HiScript II Q Select RT SuperMix (R233-01, Vazyme). RT-qPCR was conducted on Applied Biosystems systems (Thermo Scientific) with ChamQ SYBR qPCR Master Mix (Q311-02, Vazyme). The PCR cycle was 95°C for 30s, then 40 cycles of 95°C for 10s, 63°C for 10s, and 72°C for 30s. Gene expression was analyzed using the 2^−ΔΔCT^ method. Primers for mRNA: mouse-*Foxp3* (F-CCCATCCCCAGGAGTCTTG, R-ACCATGACTAGGGGCACTGTA), mouse-H3 (F-CTGATCCGCAAGCTGCCGTTC, R-GTTGGTGTCCTCAAACAGACCC), mouse-*Ltbr* (F-CCCCTTATCGCATAGAAAACCAG, R-TGCATACCGCAAAGACAAACT), mouse-*Peli1* (F-GCCCCAGTAAAATATGGCGAA, R-CCCCATTTGCCTTAGGTCTTT), mouse-*Prdm1* (F-TTCTCTTGGAAAAACGTGTGGG, R-GGAGCCGGAGCTAGACTTG), mouse-*Rorc* (F- GACCCACACCTCACAAATTGA, R-AGTAGGCCACATTACACTGCT), mouse-*Stat3* (F-CAATACCATTGACCTGCCGAT, R-GAGCGACTCAAACTGCCCT), mouse-*Smurf1* (F-AGCATCAAGATCCGTCTGACA, R-CCAGAGCCGTCCACAACAAT), mouse-*Traf3* (F-CAGCCTAACCCACCCCTAAAG, R-TCTTCCACCGTCTTCACAAAC), and mouse-β-actin (F-GGCTGTATTCCCCTCCATCG, R-CCAGTTGGTAACAATGCCATGT).

**Statistical analysis**

We used SPSS 19.0 (SPSS, IL, USA) and R 4.4 software for statistical analysis, and GraphPad Prism 9.0 (GraphPad, CA, USA) and R 4.4 software to generate visual images. All data were expressed as mean ± SD and reported *P* values less than 0.05 were considered statistically significant. Normality analyses were conducted using Shapiro-Wilk test and D’Agostino and Pearson tests. When comparing two samples, the independent sample *t* test was used if the data were normally distributed, and the variances were homogeneous. Otherwise, Wilcoxon rank sum test was used. Kaplan-Meier curves with 95% confidence intervals were plotted and the Log-rank test was used to compare survival curves.

**Supplementary Figures**


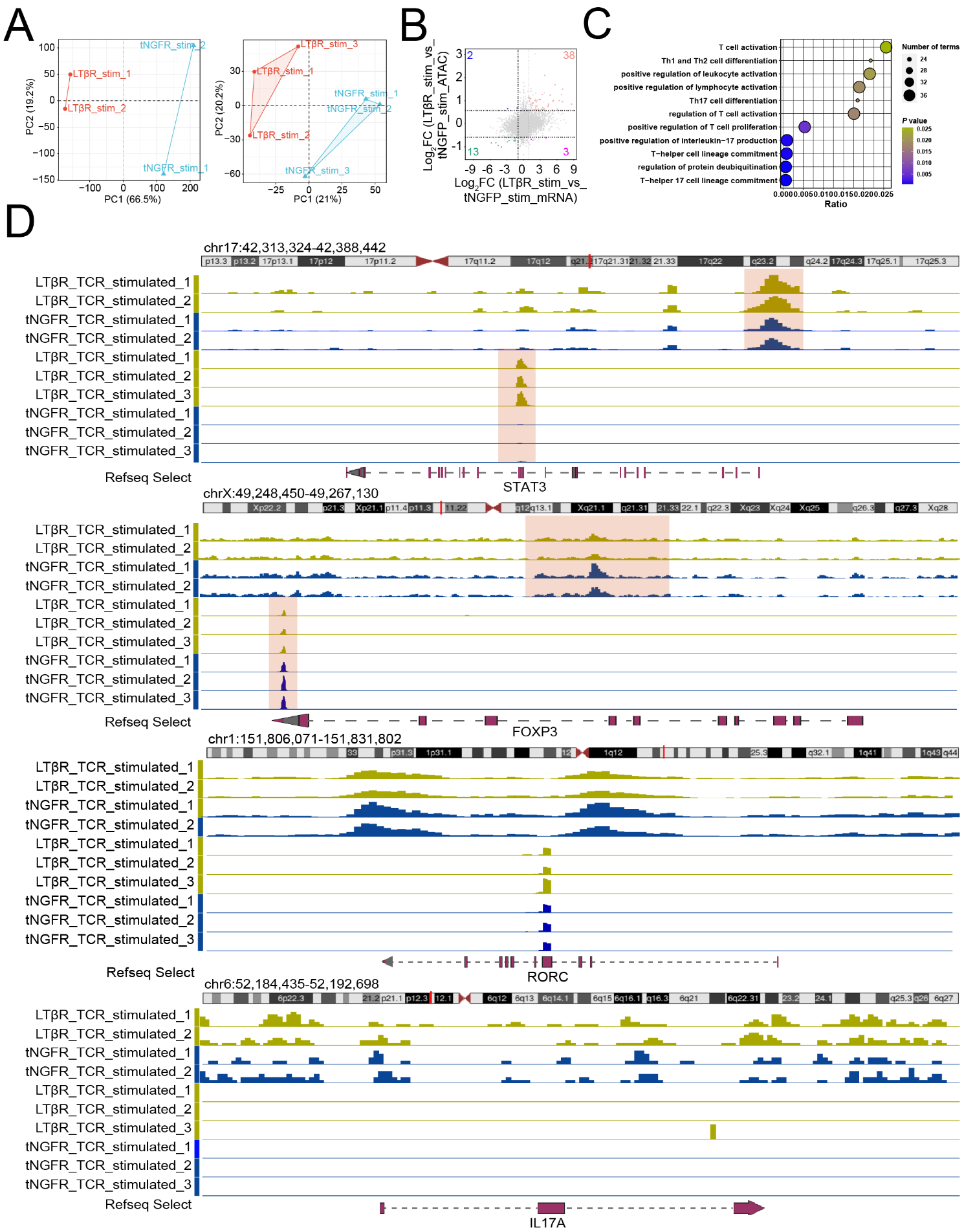


**Fig. S1. Impact of LTβR on chromatin accessibility and gene expression in CD4^+^ T cells.** **A** Principal Component Analysis was performed to compare ATAC-seq data similarity between LTβR-overexpressing and tNGFR-overexpressing TCR-stimulated CD4^+^ T cells (left) (*n* = 2), as well as to analyze transcriptomic data similarity (right) (*n* = 3). **B** The four-quadrant diagram illustrated the distribution of genes with significant alterations in both chromatin accessibility (*n* = 2) and mRNA expression (*n* = 3) in TCR-stimulated CD4^+^ T cells. **C** KEGG pathway enrichment analysis identified pathways associated with genes demonstrating increased chromatin accessibility and transcript expression in TCR-stimulated CD4^+^ T cells. **D** The snapshots emphasized the chromatin accessibility (*n* = 2) and predominant transcriptional expression (*n* = 3) of STAT3, FOXP3, RORC, and IL17A in TCR-stimulated CD4^+^ T cells. ATAC-seq, transposase-accessible chromatin by sequencing; tNGFR, truncated nerve growth factor receptor.


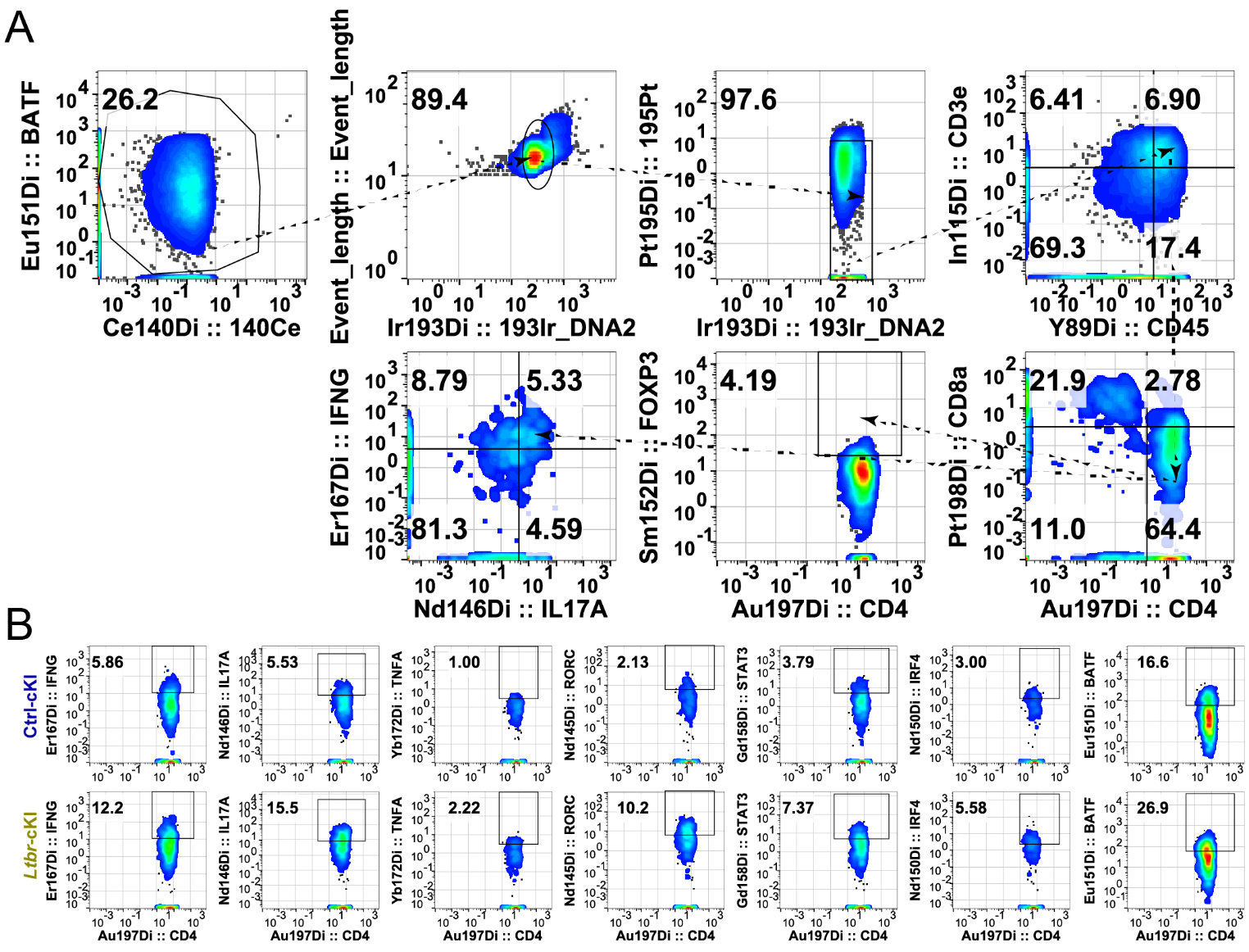


**Fig. S2 LTβR boosted the Th17/Treg cell ratio thus suppressing hepatocellular carcinoma growth.** **A** Mass cytometry gating logic highlighted marker expression in primary carcinoma-infiltrating CD4^+^ T cells. **B** A representative plot illustrated the impact of conditional knock-in of *Ltbr* in CD4^+^ T cells on the expression of T cell markers (*n* = 6).


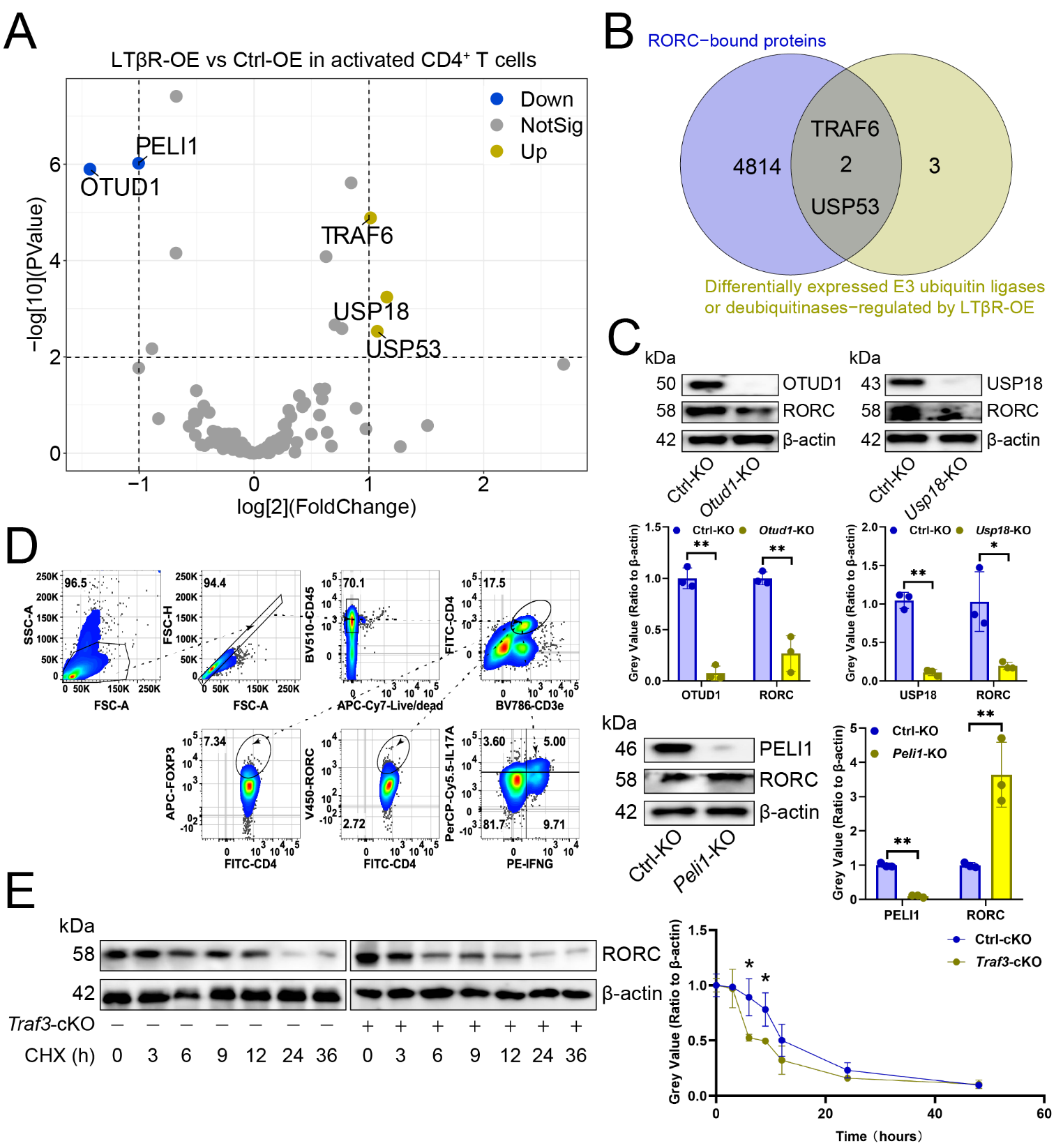


**Fig. S3 LTβR stabilized TRAF3 protein by inhibiting PELI1 expression.** **A** A volcano plot illustrated LTβR’s impact on the transcription of E3 ubiquitin ligases or deubiquitinases in CD4^+^ T cells (*n* = 3). **B** A Venn diagram showed the overlap between RORC-binding proteins identified by mass spectrometry and E3 ubiquitin ligases or deubiquitinases regulated by LTβR overexpression in CD4^+^ T cells. **C** Effects of *Otud1*, *Usp18*, or *Peli1* knockout on RORC protein expression in CD4^+^ T cells (*n* = 3). **D** Flow cytometry gating logic revealed Th17 cell infiltration in hepatocellular carcinoma. **E** CD4^+^ T cells with *Traf3* knockout were subjected to Cycloheximide treatment to evaluate the influence of TRAF3 on the stability of RORC protein (*n* = 3). **A**, **C**, **E** represented mean ± SD analyzed by unpaired *t* test. **P* <0.05, ***P* <0.01.


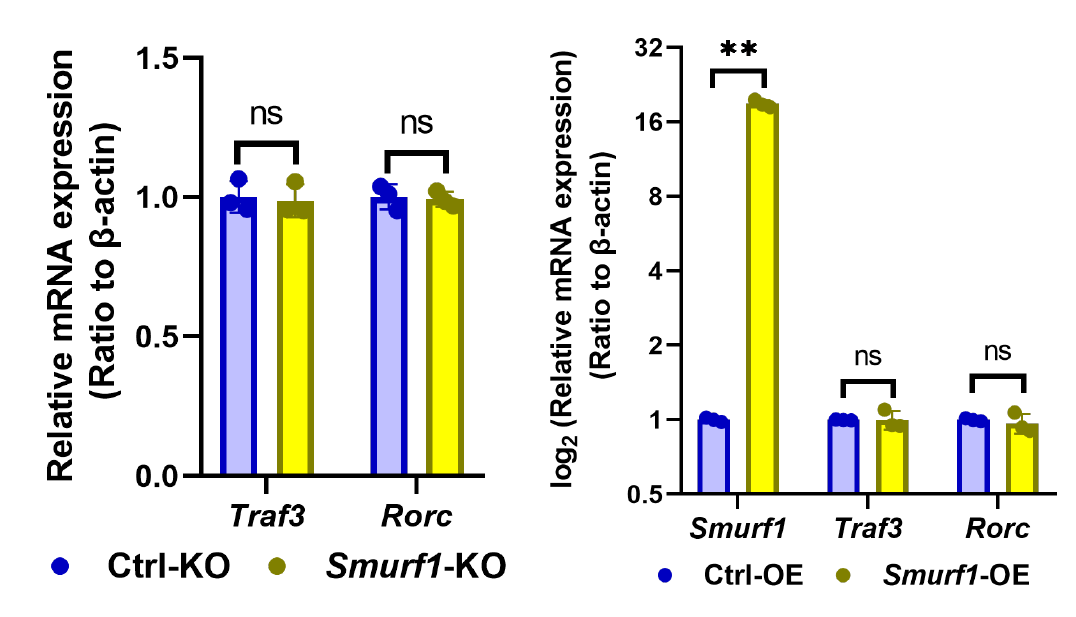


**Fig. S4. TRAF3 competed with RORC for SMURF1 binding.** RT-qPCR showed SMURF1’s impact on *Traf3* or *Rorc* mRNA expression in Th17 cells (*n* = 3). Data represented mean ± SD analyzed by unpaired *t* test. **P* <0.05, ***P* <0.01.


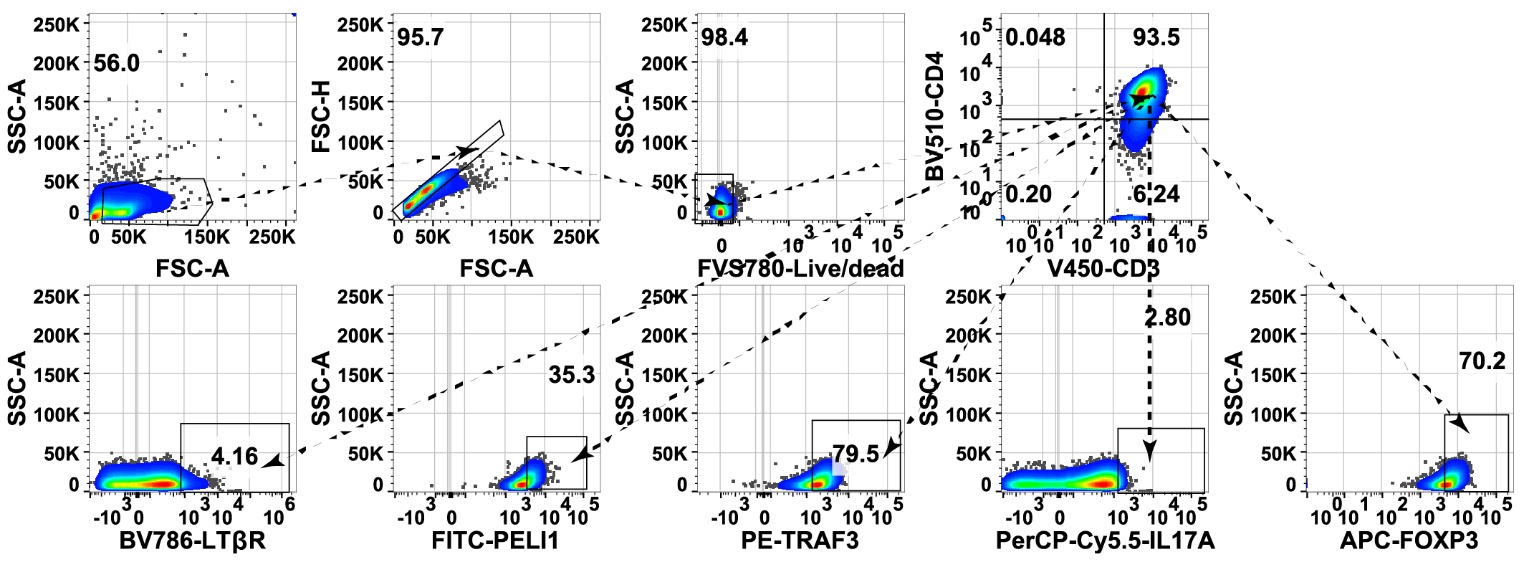


Fig. S5. Flow cytometry gating logic assessed the impact of N-glycosylation inhibitors (Tunicamycin, PNGase F, and Swainsonine) and glucose deprivation on marker expression in primary carcinoma-infiltrating CD4^+^ T cells.


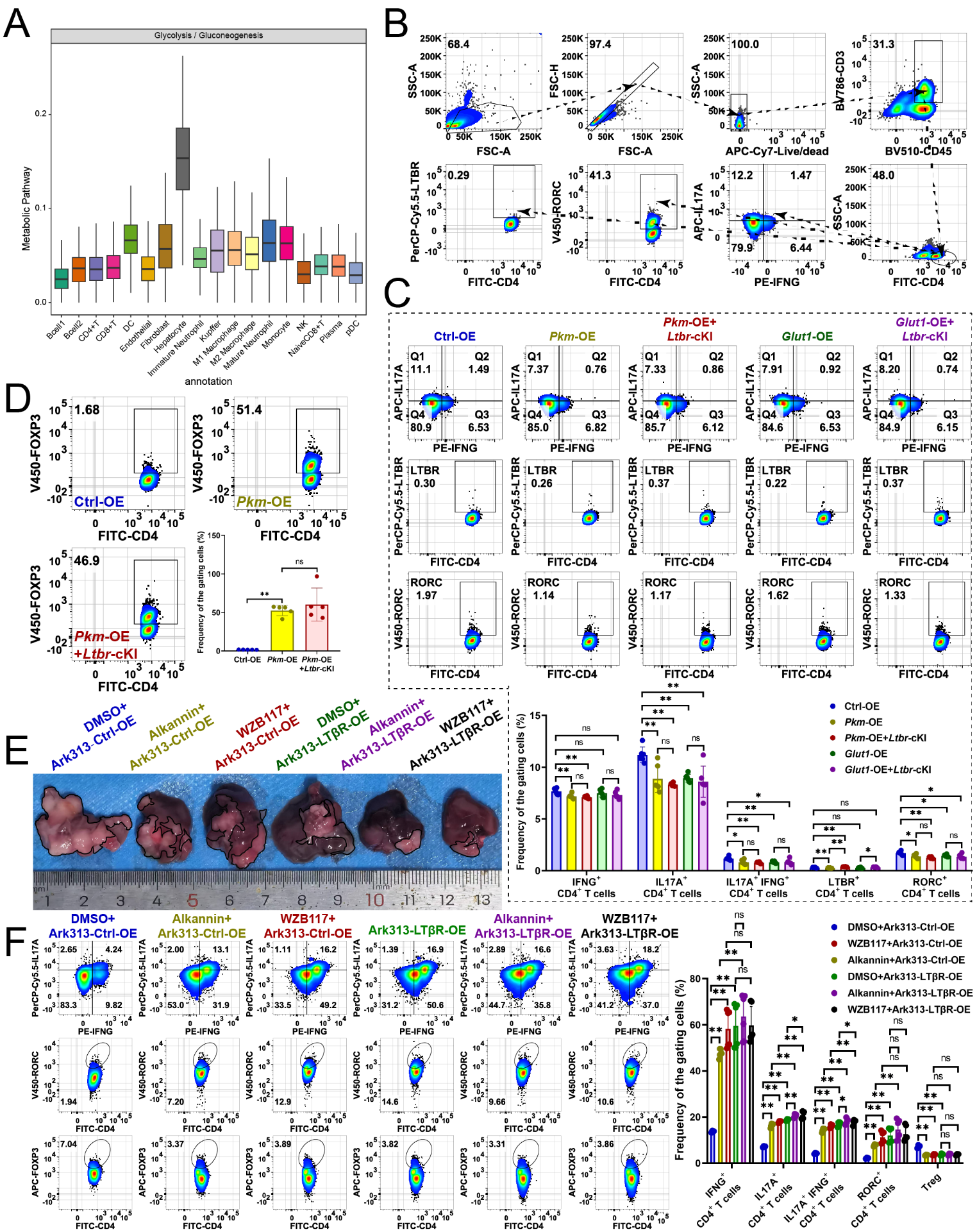


**Fig. S6. LTβR improved the immunotherapy efficacy of glycolysis inhibitors.** **A** ScMetabolism analysis of glycolysis/gluconeogenesis pathway scores of various cells in HCC (*n* = 3). **B-D** Flow cytometry revealed how HCC cells which overexpressed *Pkm* or *Glut1* with *Ltbr*-cKI in CD4^+^ T cells affected Th17/Treg cell infiltration and RORC expression. (B) Gating logic. (C, D) Representative plots and quantitative analysis of Th17 cells (D) or Treg cells (E) infiltration (*n* = 5). **E** Influence of Food and Drug Administration-approved glycolysis inhibitors with LTβR-overexpressing T cells on patient-derived orthotopic xenograft growth (*n* = 5). **F** Flow cytometry analysis of glycolysis inhibitors and LTβR-overexpressing T cells on Th17/Treg cell infiltration and RORC expression (*n* = 5). **C**, **D**, **F** represented mean ± SD analyzed by unpaired *t* test. **P* <0.05, ***P* <0.01. HCC, hepatocellular carcinoma.


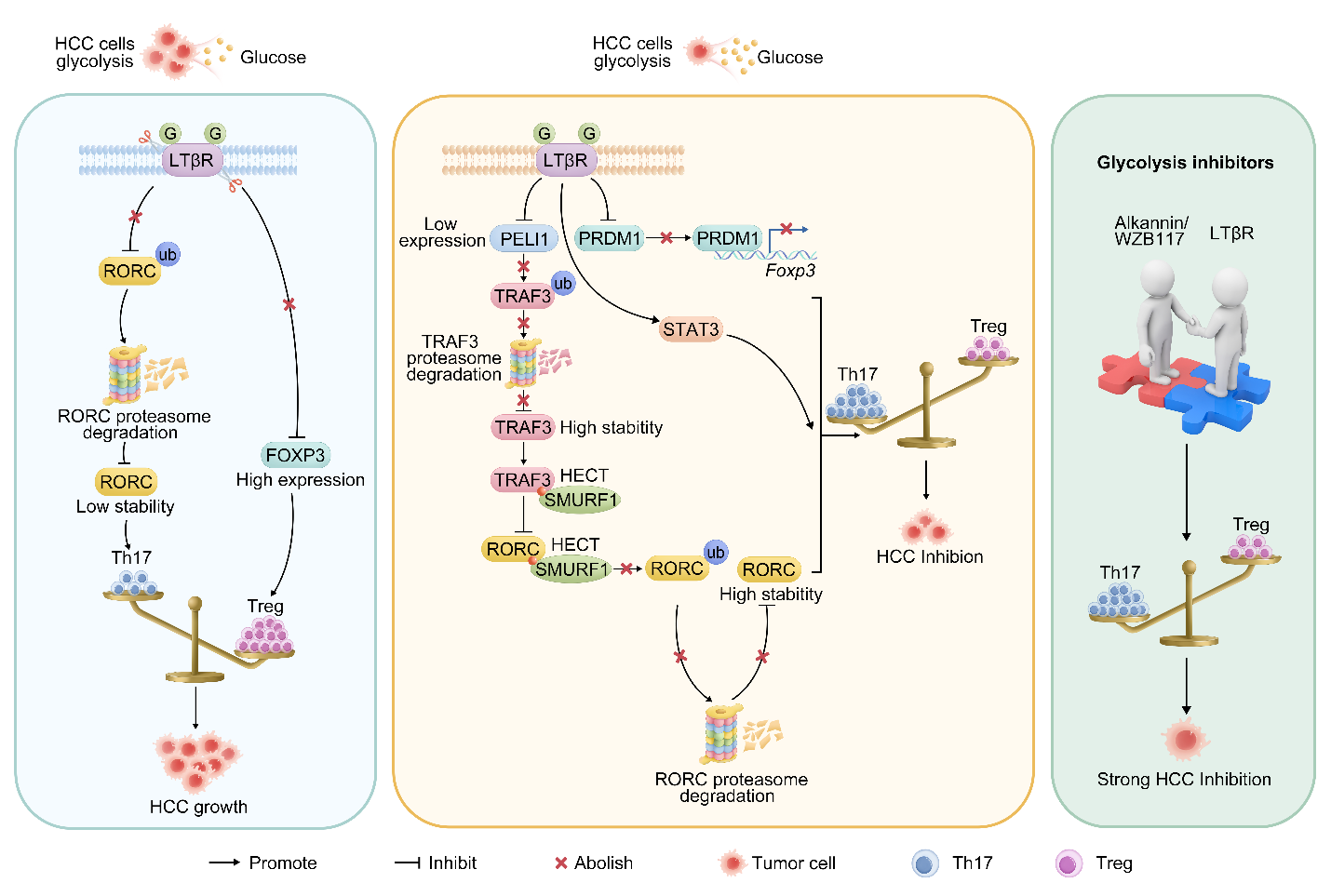


**Fig. S7. Mechanism diagram.**

**Supplementary Table**

**Table S1. Sequence of potential PRDM1 binding to FOXP3 promoter.**

| Sequence ID | 5’-3’ |
| --- | --- |
| Sequence_1 | AGATGAGGAAAGTCAGTCTCTTTTTTG |
| Sequence_2 | AAAAAGTGCAAATGAGGGAAAGAGC |
| Sequence_3 | CTACTTTCTCTTCCTC |
| Sequence_4 | GCCTTTCTTCC |
| Sequence_5 | TTTTTTCTCCATGAATTGCTTTCCATGCCT |
| Sequence_6 | GAAAGAAAGGC |
| Sequence_7 | TGGAGACAGAG |
| Sequence_8 | TGCTGTCTCTA |
| Sequence_9 | TGGTTTCTCAT |

**Supplementary References**

1. Langmead B, Salzberg SL. Fast gapped-read alignment with Bowtie 2. Nat Methods. 2012;9(4):357-9.

2. Schneider VA, Graves-Lindsay T, Howe K, Bouk N, Chen HC, Kitts PA, et al. Evaluation of GRCh38 and de novo haploid genome assemblies demonstrates the enduring quality of the reference assembly. Genome Res. 2017;27(5):849-64.

3. Li H, Handsaker B, Wysoker A, Fennell T, Ruan J, Homer N, et al. The Sequence Alignment/Map format and SAMtools. Bioinformatics. 2009;25(16):2078-9.

4. Zhang Y, Liu T, Meyer CA, Eeckhoute J, Johnson DS, Bernstein BE, et al. Model-based analysis of ChIP-Seq (MACS). Genome Biol. 2008;9(9):R137.

5. Quinlan AR, Hall IM. BEDTools: a flexible suite of utilities for comparing genomic features. Bioinformatics. 2010;26(6):841-2.

6. Love MI, Huber W, Anders S. Moderated estimation of fold change and dispersion for RNA-seq data with DESeq2. Genome Biol. 2014;15(12):550.

7. Ramírez F, Dündar F, Diehl S, Grüning BA, Manke T. deepTools: a flexible platform for exploring deep-sequencing data. Nucleic Acids Res. 2014;42(Web Server issue):W187-91.

8. Lopez-Delisle L, Rabbani L, Wolff J, Bhardwaj V, Backofen R, Grüning B, et al. pyGenomeTracks: reproducible plots for multivariate genomic datasets. Bioinformatics. 2021;37(3):422-3.

9. Nyberg WA, Ark J, To A, Clouden S, Reeder G, Muldoon JJ, et al. An evolved AAV variant enables efficient genetic engineering of murine T cells. Cell. 2023;186(2):446-60.e19.

10. Finck R, Simonds EF, Jager A, Krishnaswamy S, Sachs K, Fantl W, et al. Normalization of mass cytometry data with bead standards. Cytometry A. 2013;83(5):483-94.

11. Hahne F, LeMeur N, Brinkman RR, Ellis B, Haaland P, Sarkar D, et al. flowCore: a Bioconductor package for high throughput flow cytometry. BMC Bioinformatics. 2009;10:106.

12. Finak G, Jiang W, Pardo J, Asare A, Gottardo R. QUAliFiER: an automated pipeline for quality assessment of gated flow cytometry data. BMC Bioinformatics. 2012;13:252.

13. Bendall SC, Simonds EF, Qiu P, Amir el AD, Krutzik PO, Finck R, et al. Single-cell mass cytometry of differential immune and drug responses across a human hematopoietic continuum. Science. 2011;332(6030):687-96.

14. Van Gassen S, Callebaut B, Van Helden MJ, Lambrecht BN, Demeester P, Dhaene T, et al. FlowSOM: Using self-organizing maps for visualization and interpretation of cytometry data. Cytometry A. 2015;87(7):636-45.

15. Cieslak MC, Castelfranco AM, Roncalli V, Lenz PH, Hartline DK. t-Distributed Stochastic Neighbor Embedding (t-SNE): A tool for eco-physiological transcriptomic analysis. Mar Genomics. 2020;51:100723.

16. Wu Y, Yang S, Ma J, Chen Z, Song G, Rao D, et al. Spatiotemporal Immune Landscape of Colorectal Cancer Liver Metastasis at Single-Cell Level. Cancer Discov. 2022;12(1):134-53.

17. Alghamdi N, Chang W, Dang P, Lu X, Wan C, Gampala S, et al. A graph neural network model to estimate cell-wise metabolic flux using single-cell RNA-seq data. Genome Res. 2021;31(10):1867-84.

18. Wang J, Wang J, Huang Y, Xiao Y. 3dRNA v2.0: An Updated Web Server for RNA 3D Structure Prediction. Int J Mol Sci. 2019;20(17).

19. Zhou X, Zheng W, Li Y, Pearce R, Zhang C, Bell EW, et al. I-TASSER-MTD: a deep-learning-based platform for multi-domain protein structure and function prediction. Nat Protoc. 2022;17(10):2326-53.

20. Eberhardt J, Santos-Martins D, Tillack AF, Forli S. AutoDock Vina 1.2.0: New Docking Methods, Expanded Force Field, and Python Bindings. J Chem Inf Model. 2021;61(8):3891-8.

21. Laskowski RA, Swindells MB. LigPlot+: multiple ligand-protein interaction diagrams for drug discovery. J Chem Inf Model. 2011;51(10):2778-86.
