# Supplementary material for: N-glycosylated LTβR increases the Th17/Treg cell ratio in liver cancer by blocking RORC ubiquitination and FOXP3 transcription
Source: Cell Death Dis. 2025 May 28;16(1):421. doi: 10.1038/s41419-025-07738-2 (PMC12120105; doi:10.1038/s41419-025-07738-2)

Figure 1N

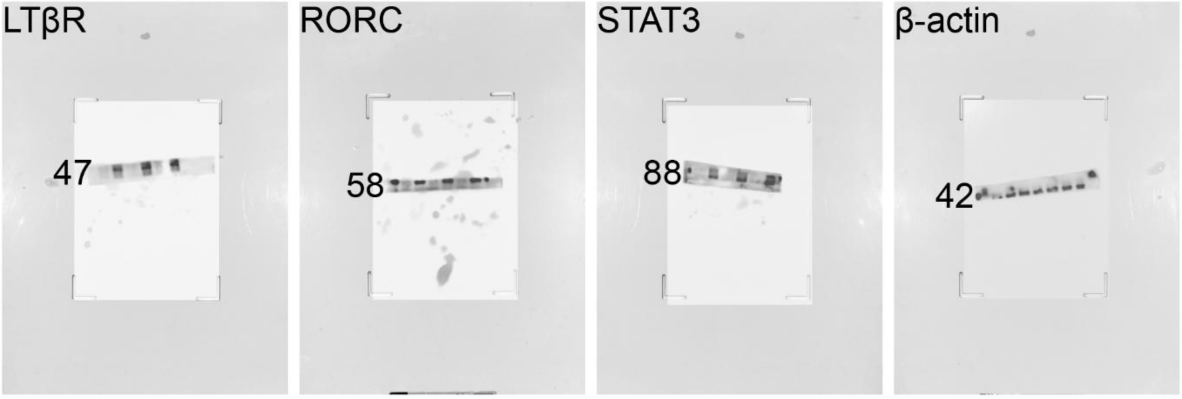

Figure 2C

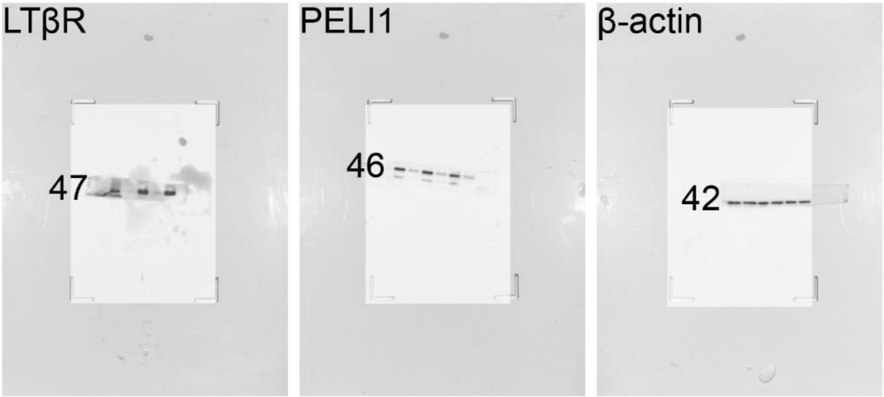

Figure 2R

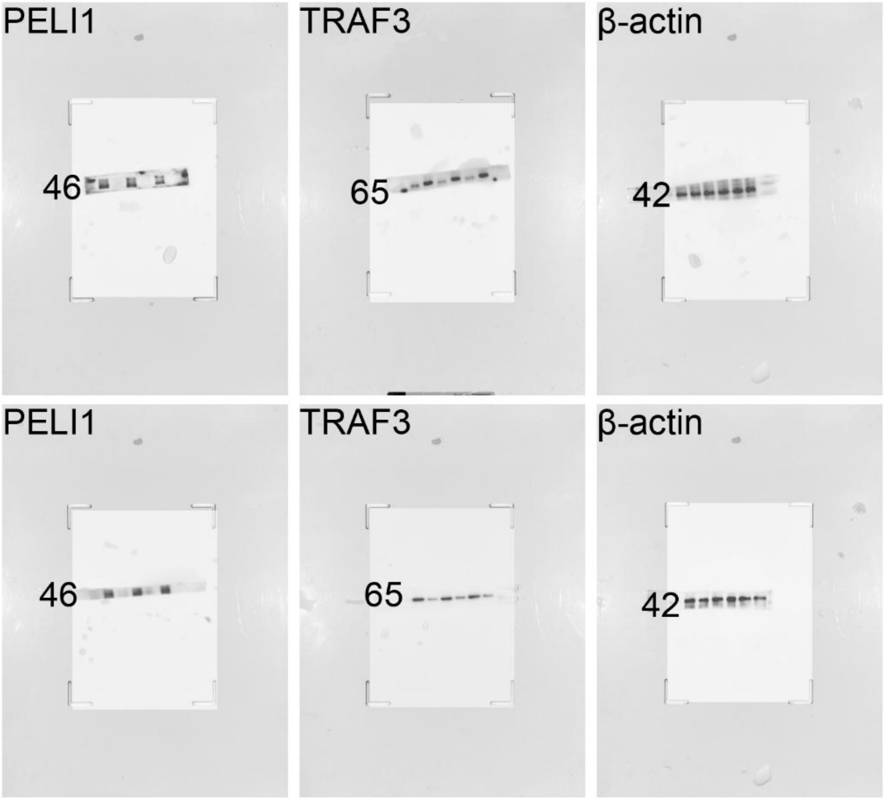

Figure 2S

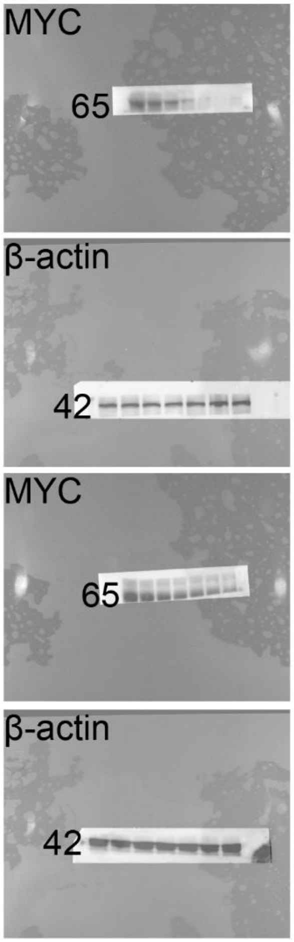

Figure 4B

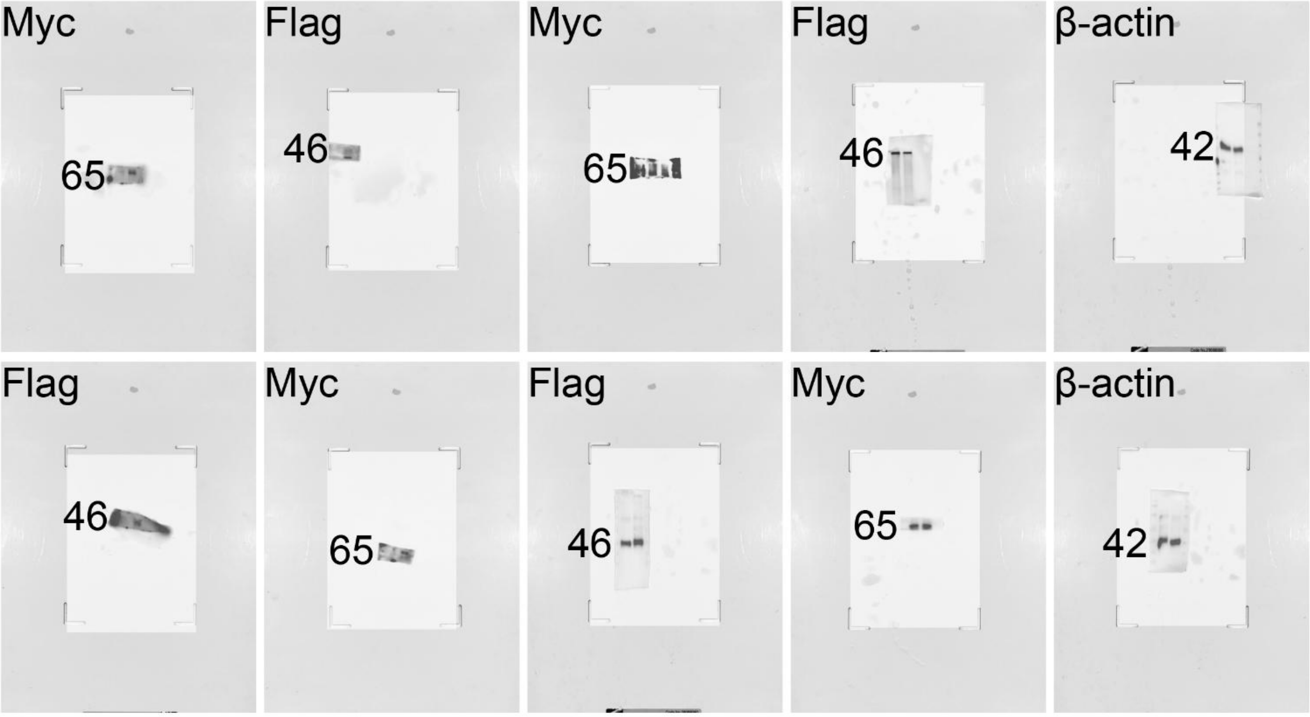

Figure 4E

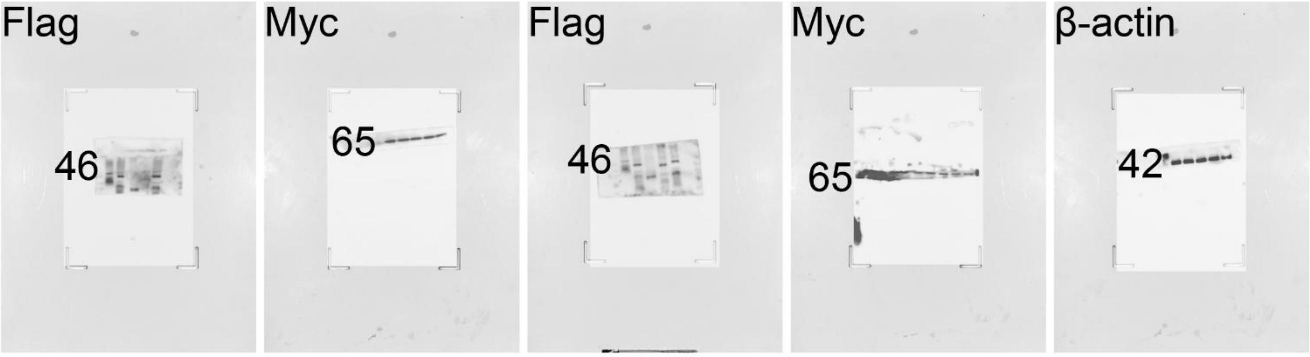

Figure 4F

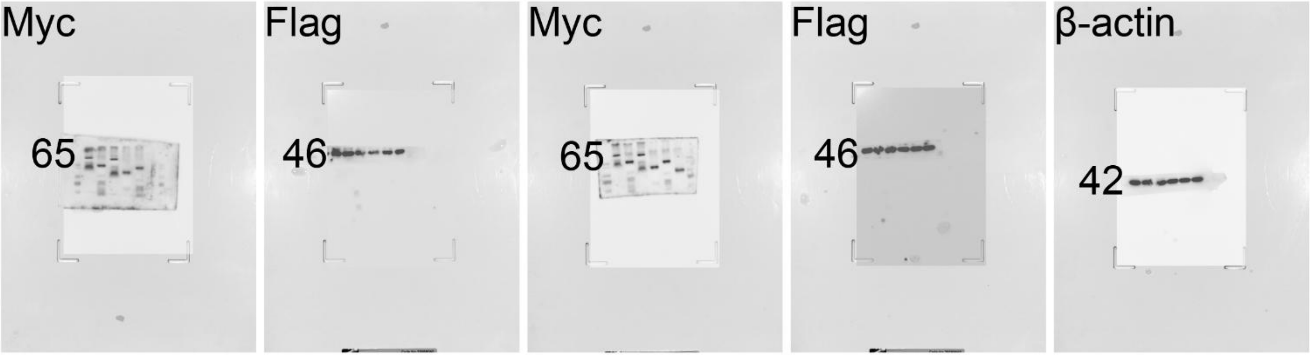

Figure 4G

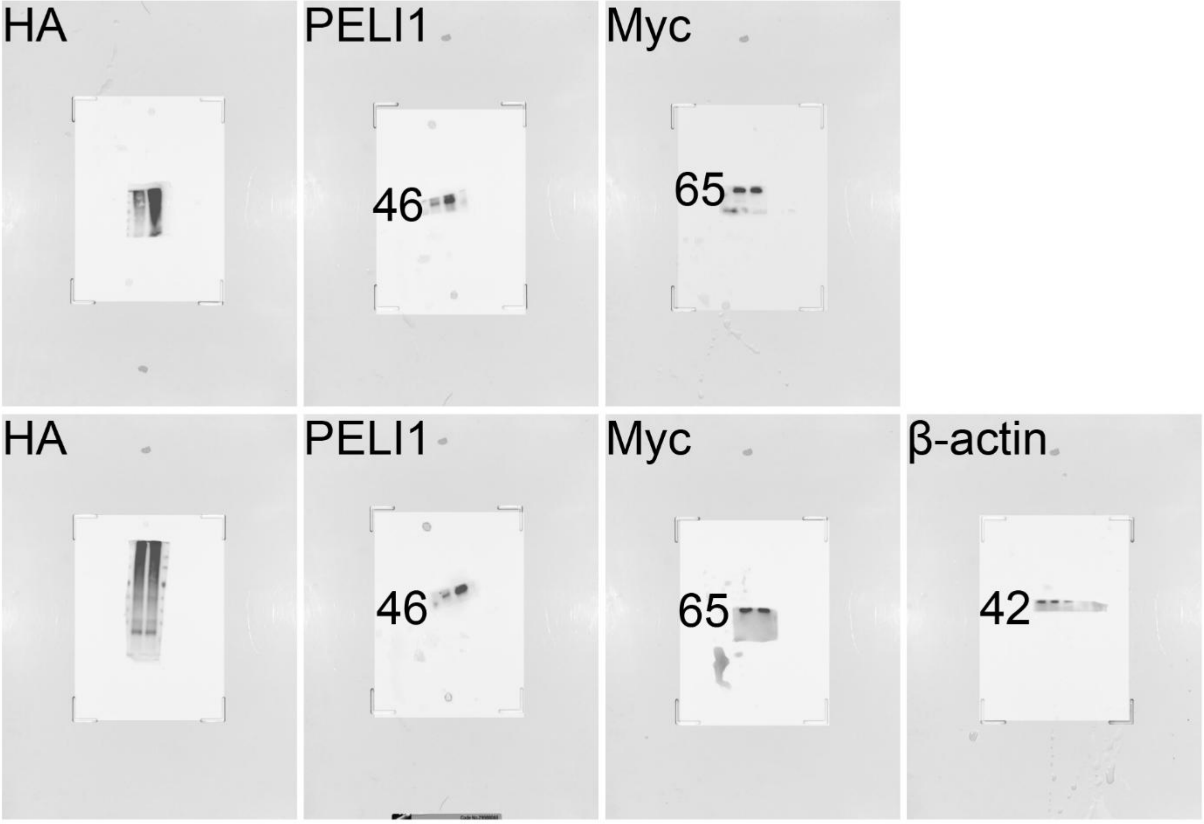

Figure 4H

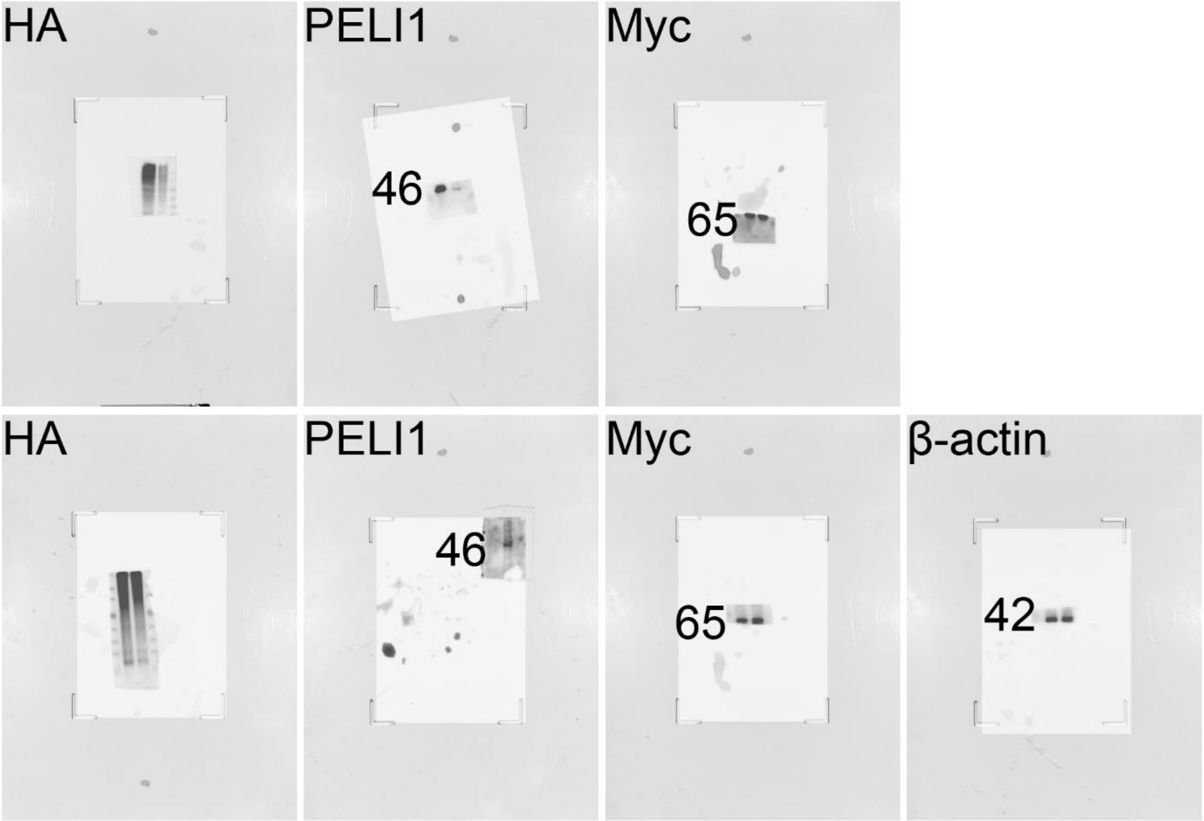

Figure 4I

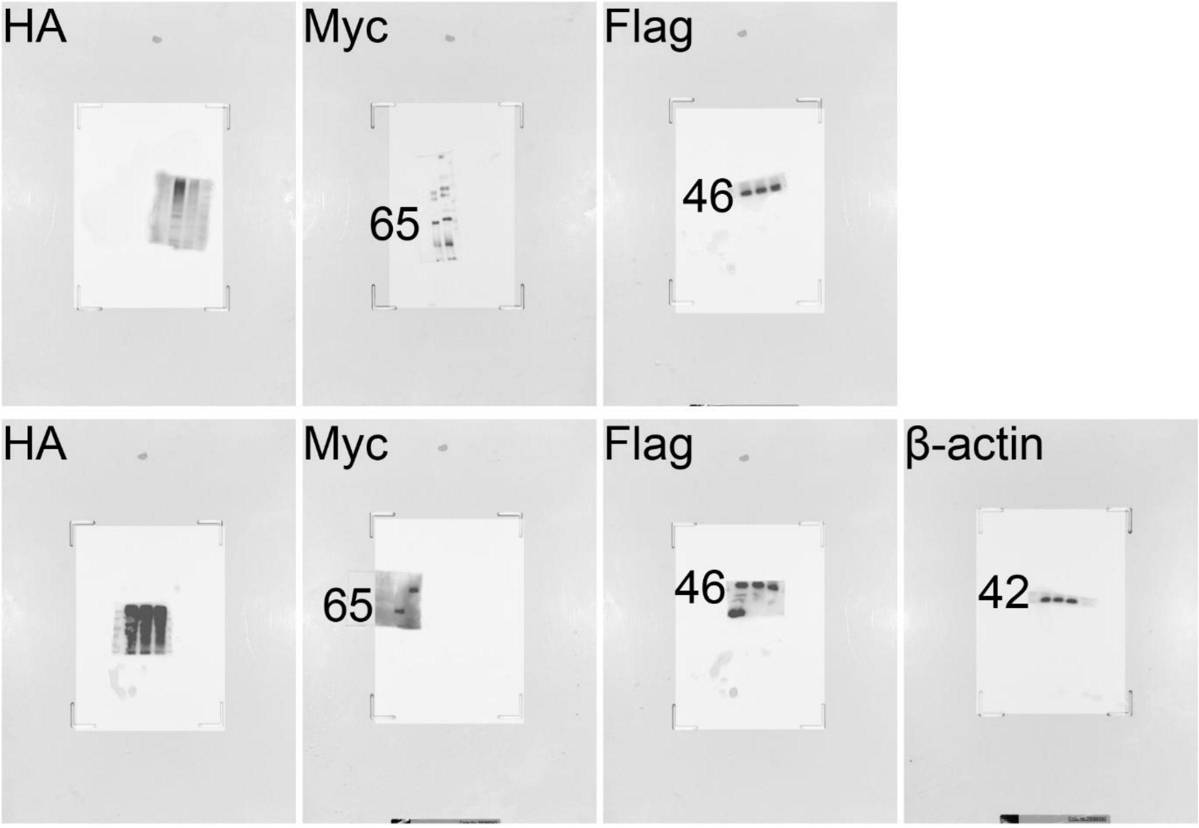

Figure 4J

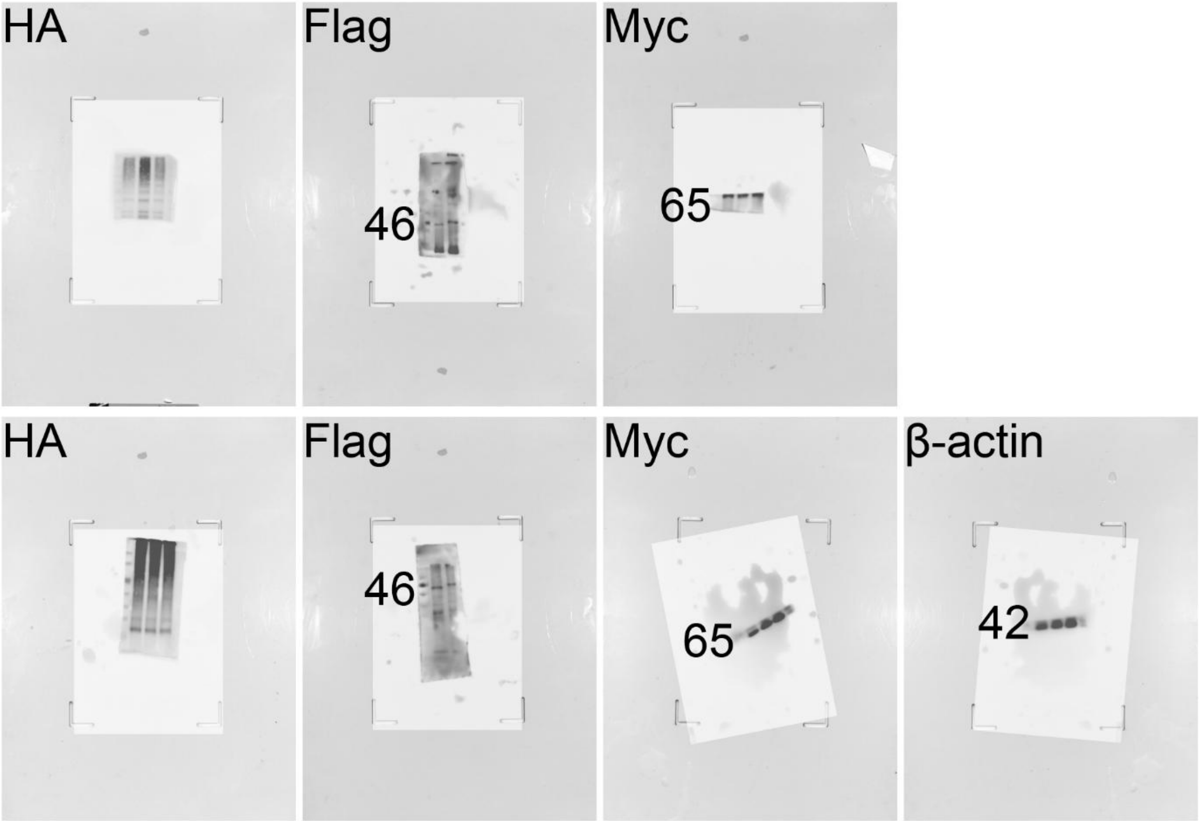

Figure 4K

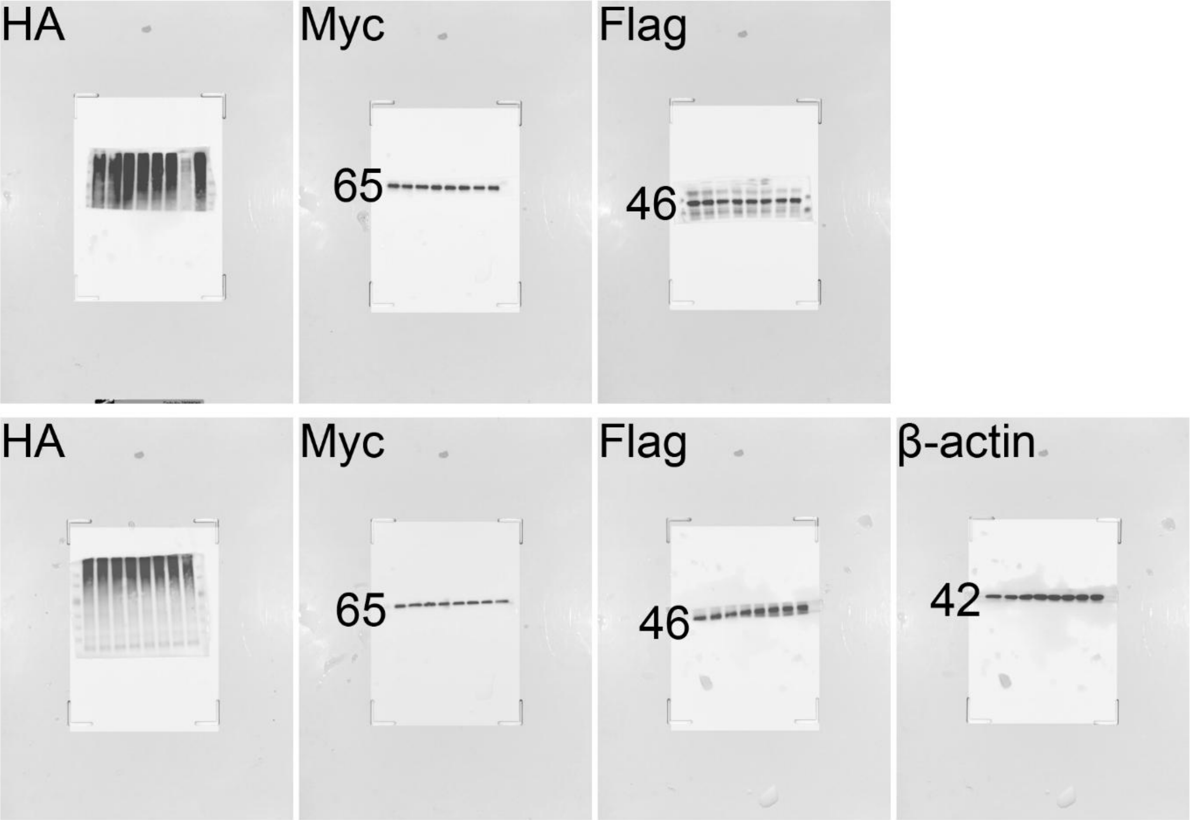

Figure 4L

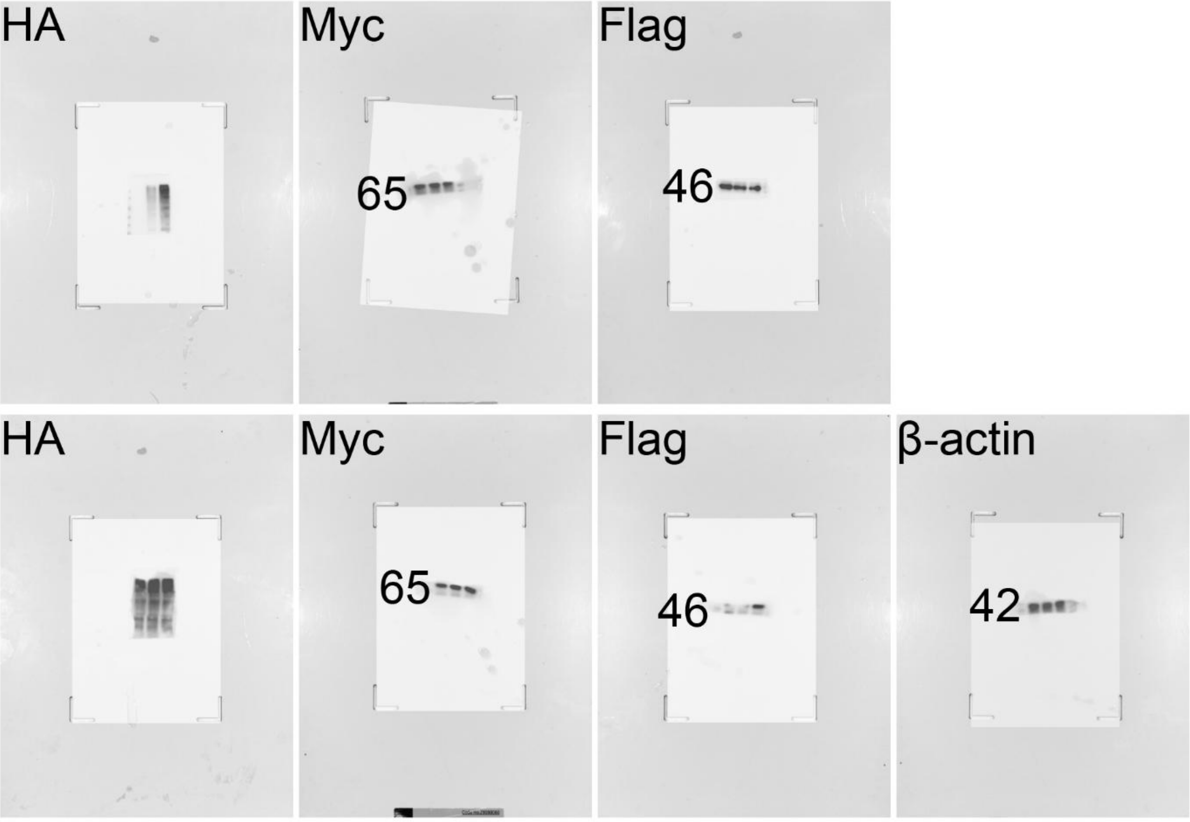

Figure 4M

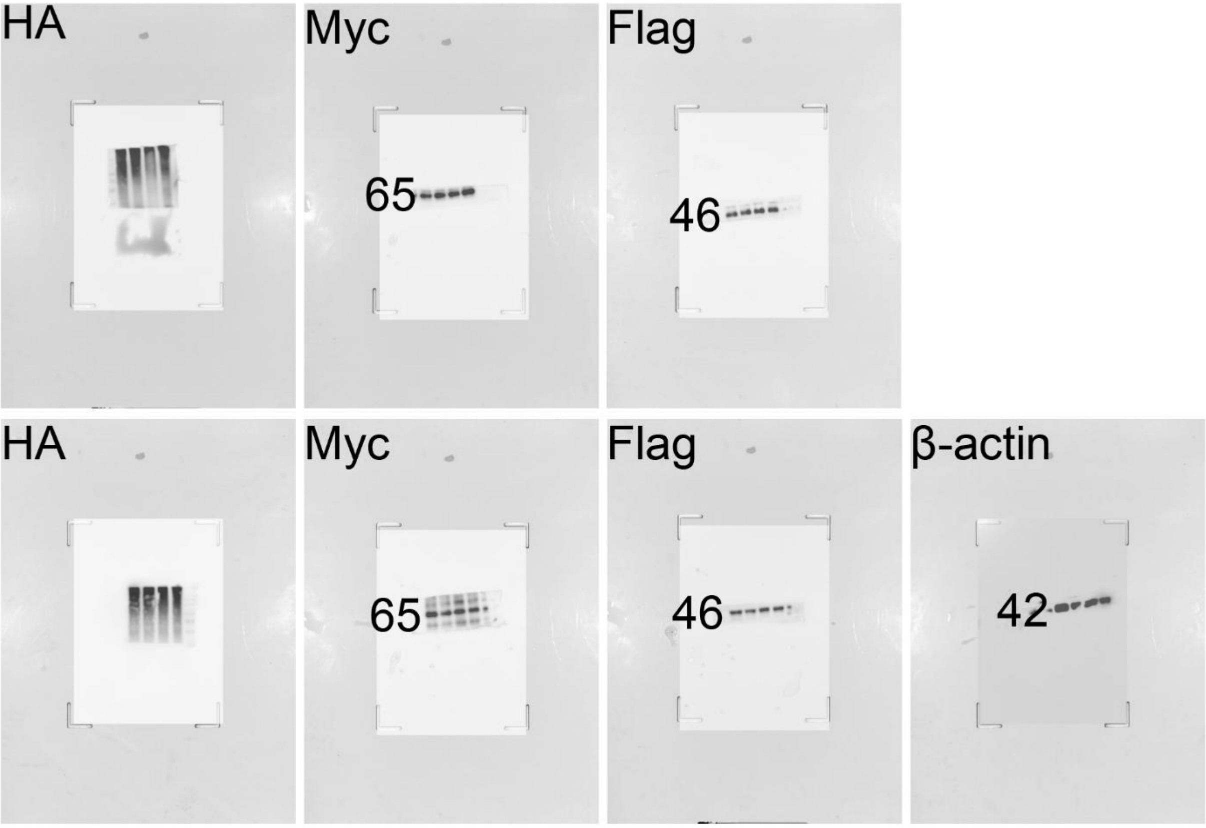

Figure 5B

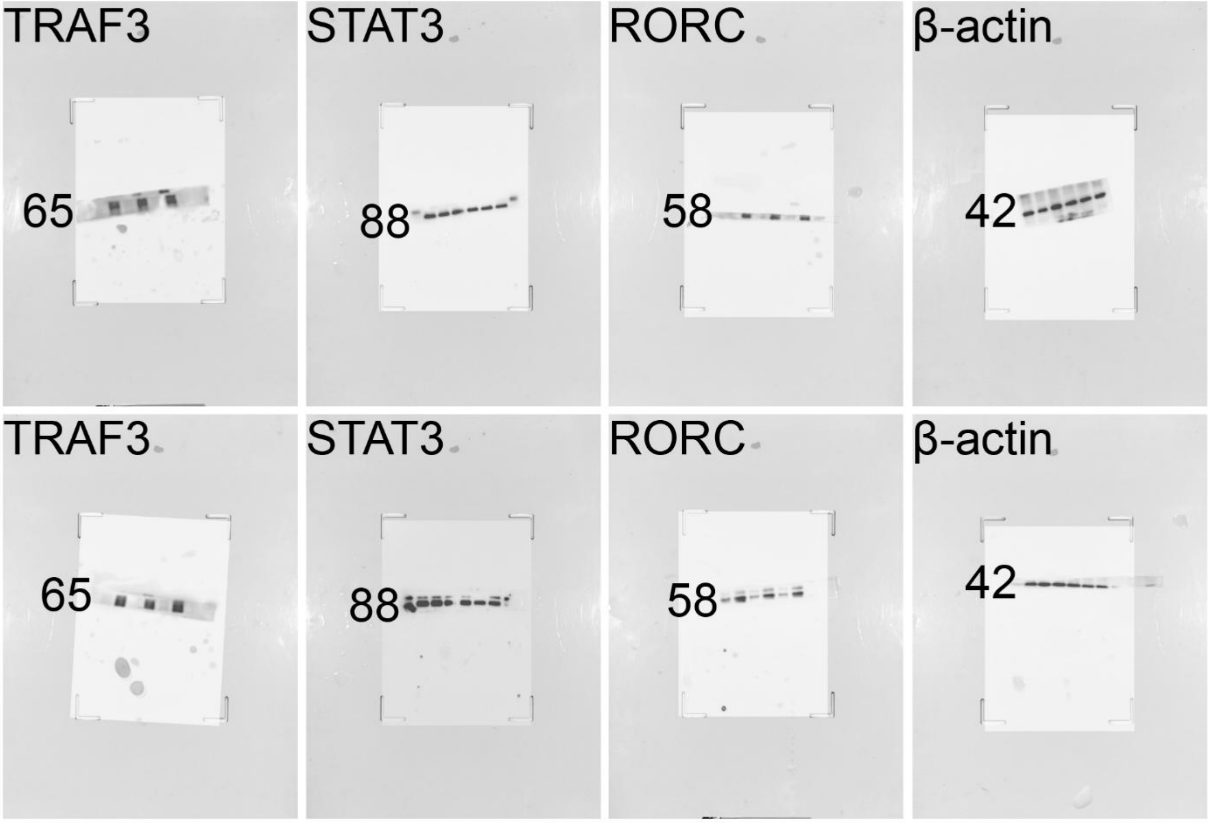

Figure 5E

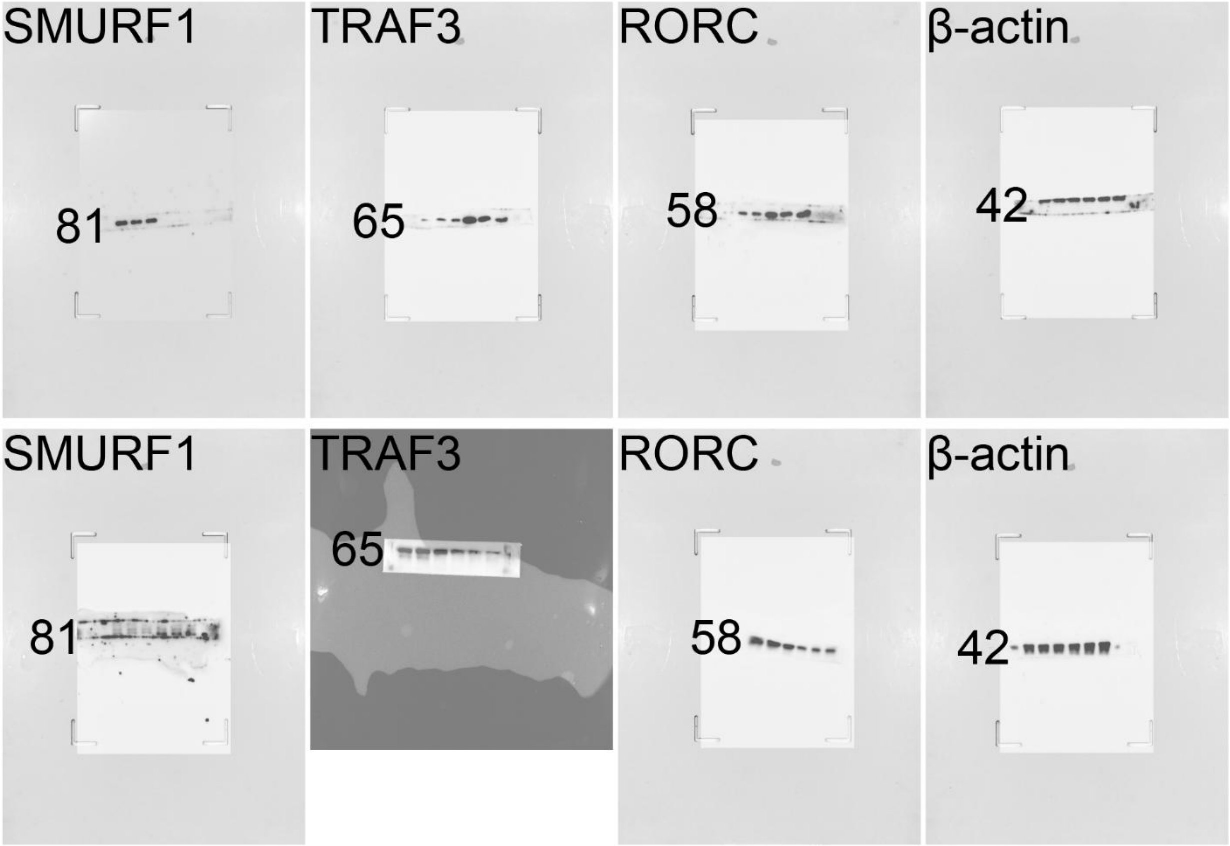

Figure 5F

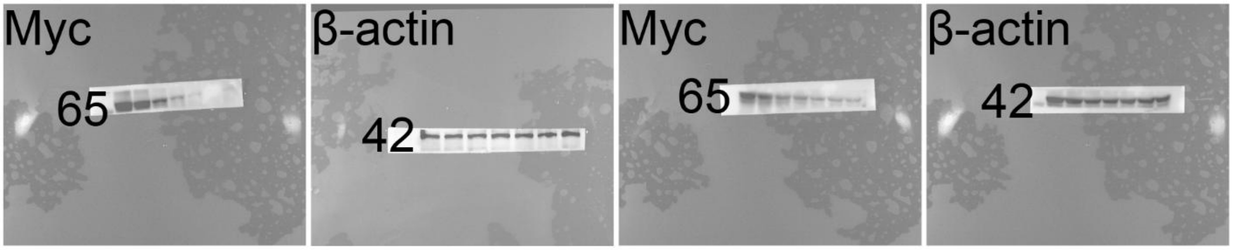

Figure 5G

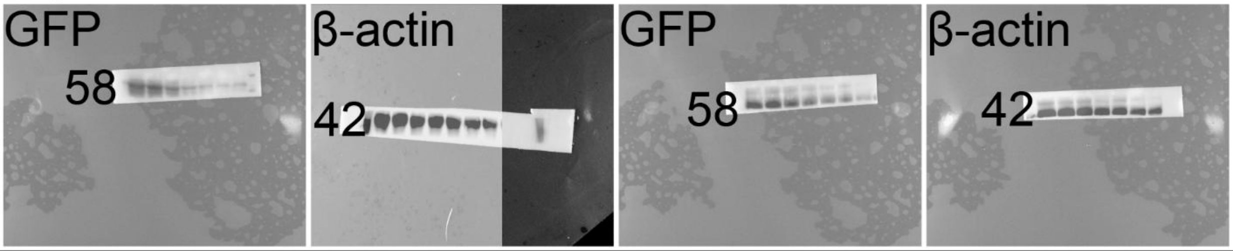

Figure 5J

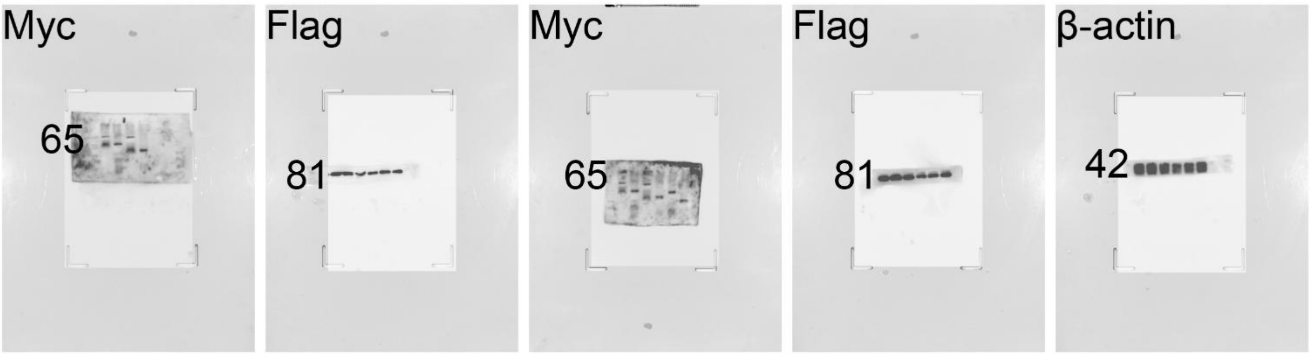

Figure 5K

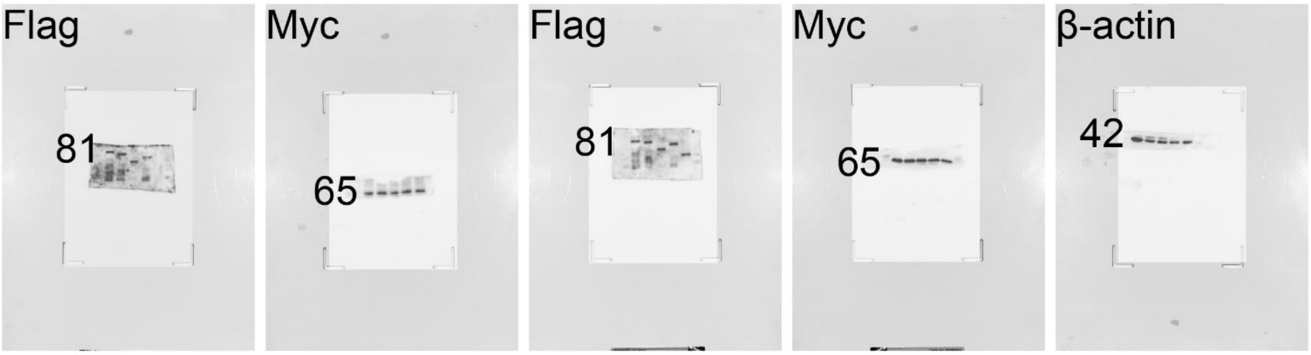

Figure 5L

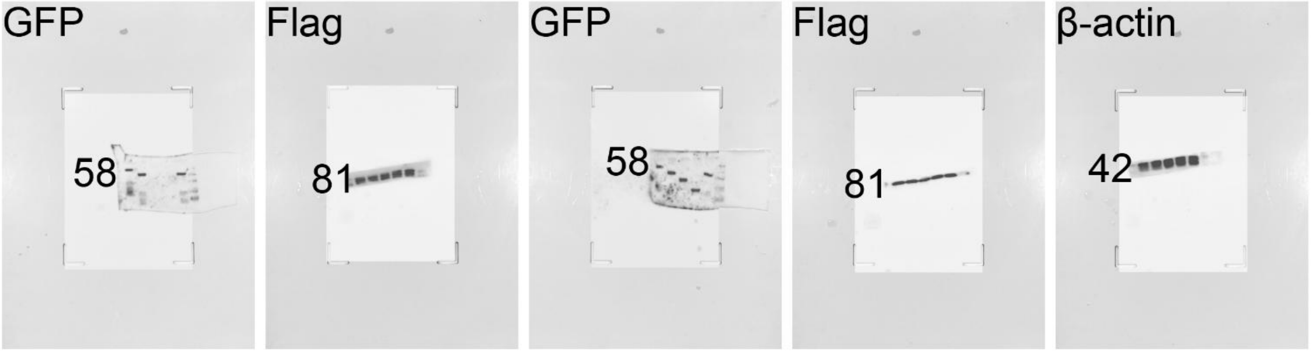

Figure 5M

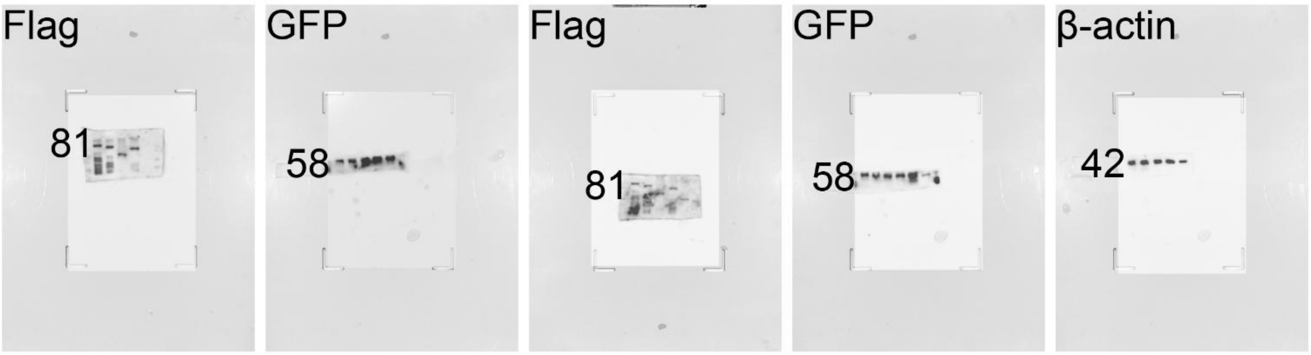

Figure 5N

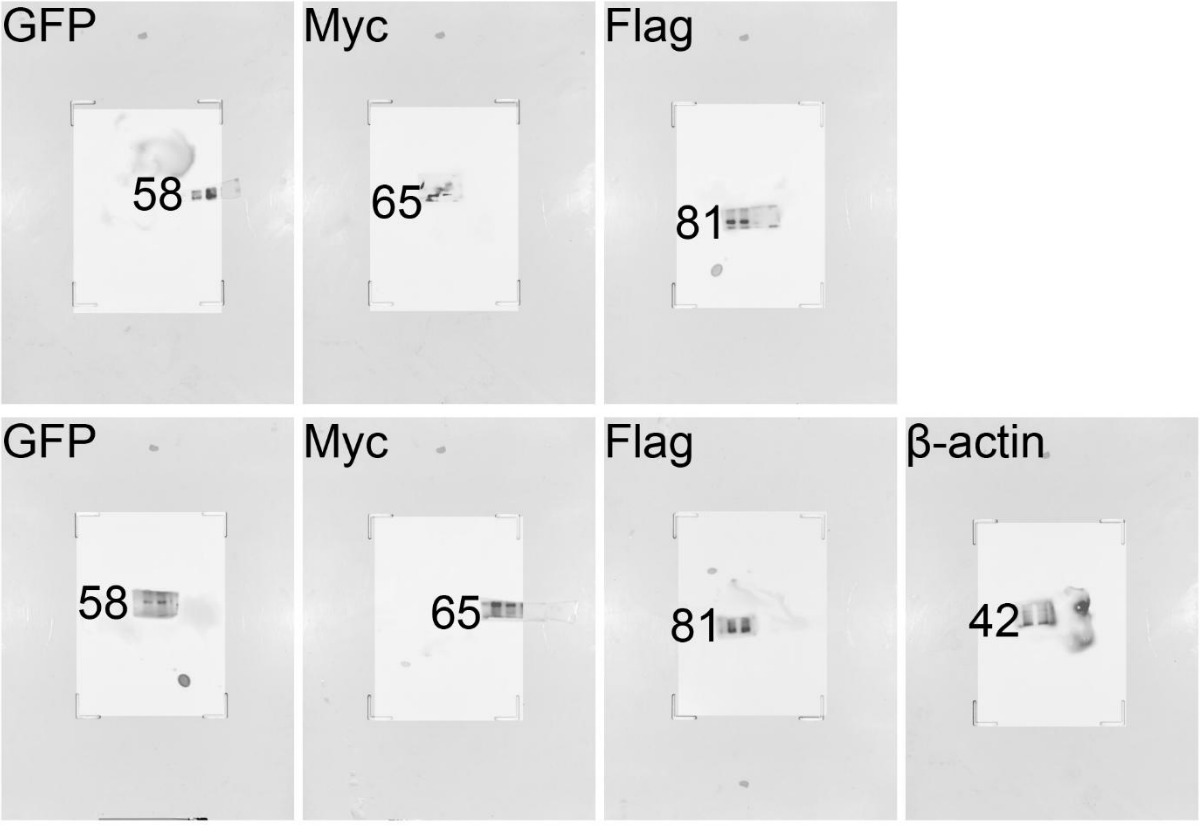

Figure 5N

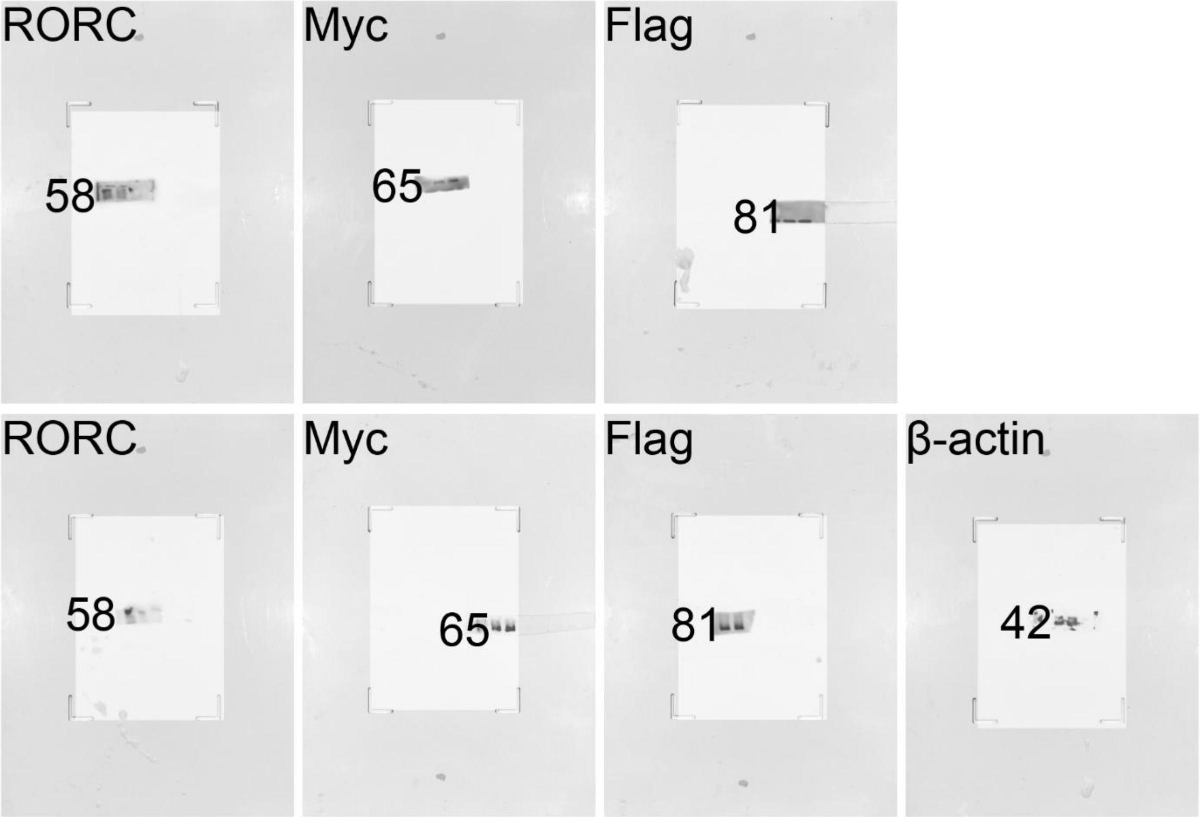

Figure 5O

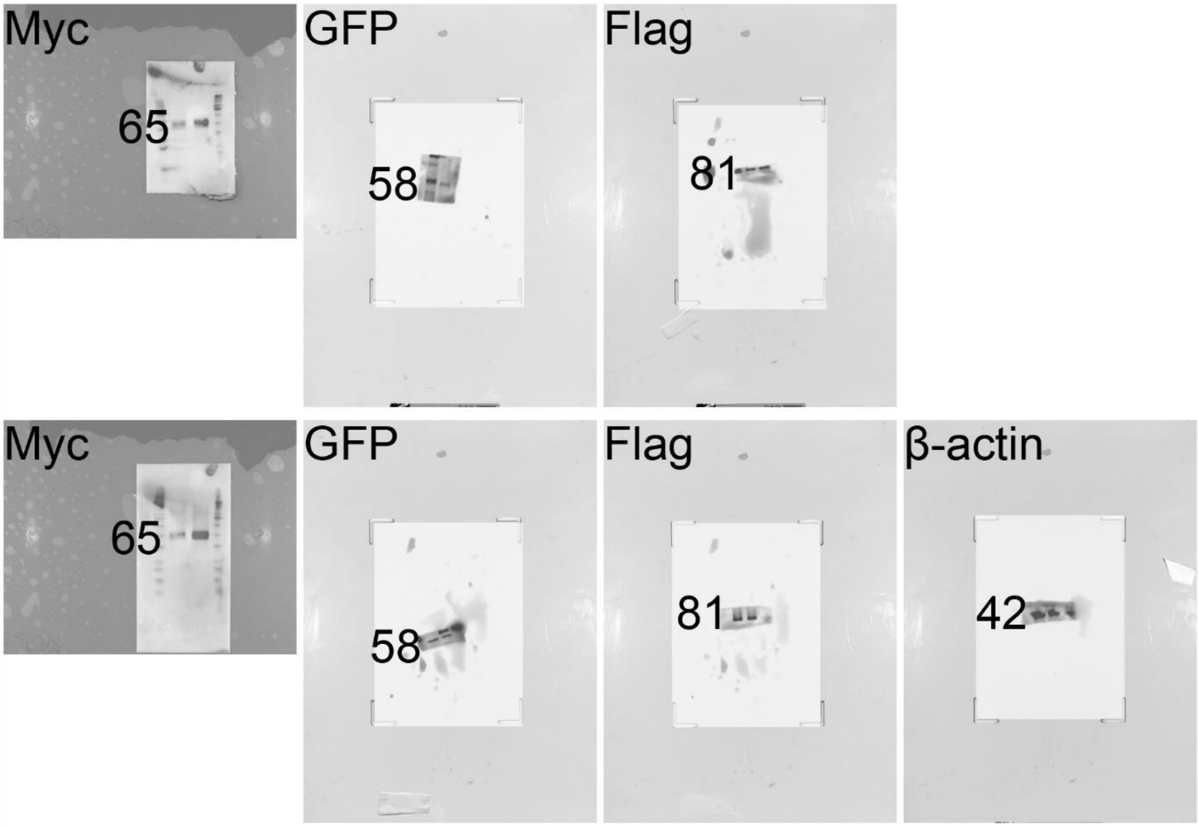

Figure 5O

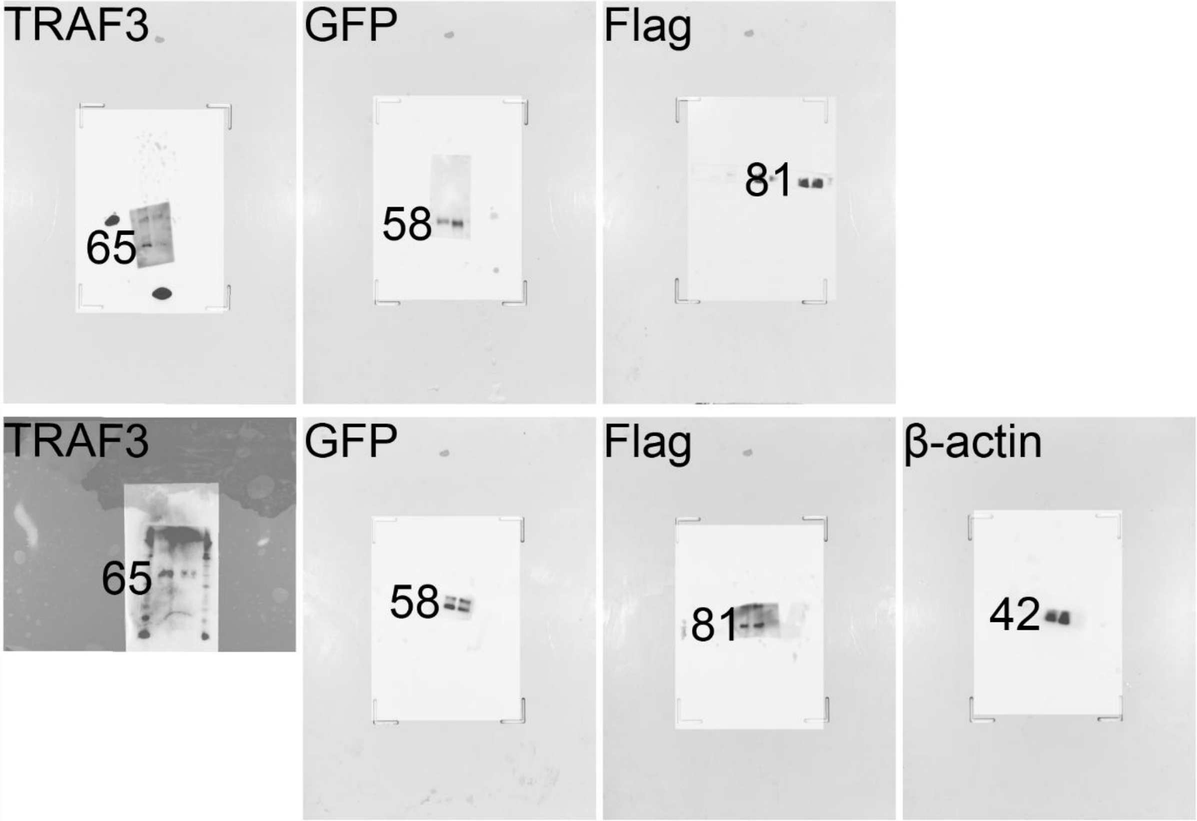

Figure 6A

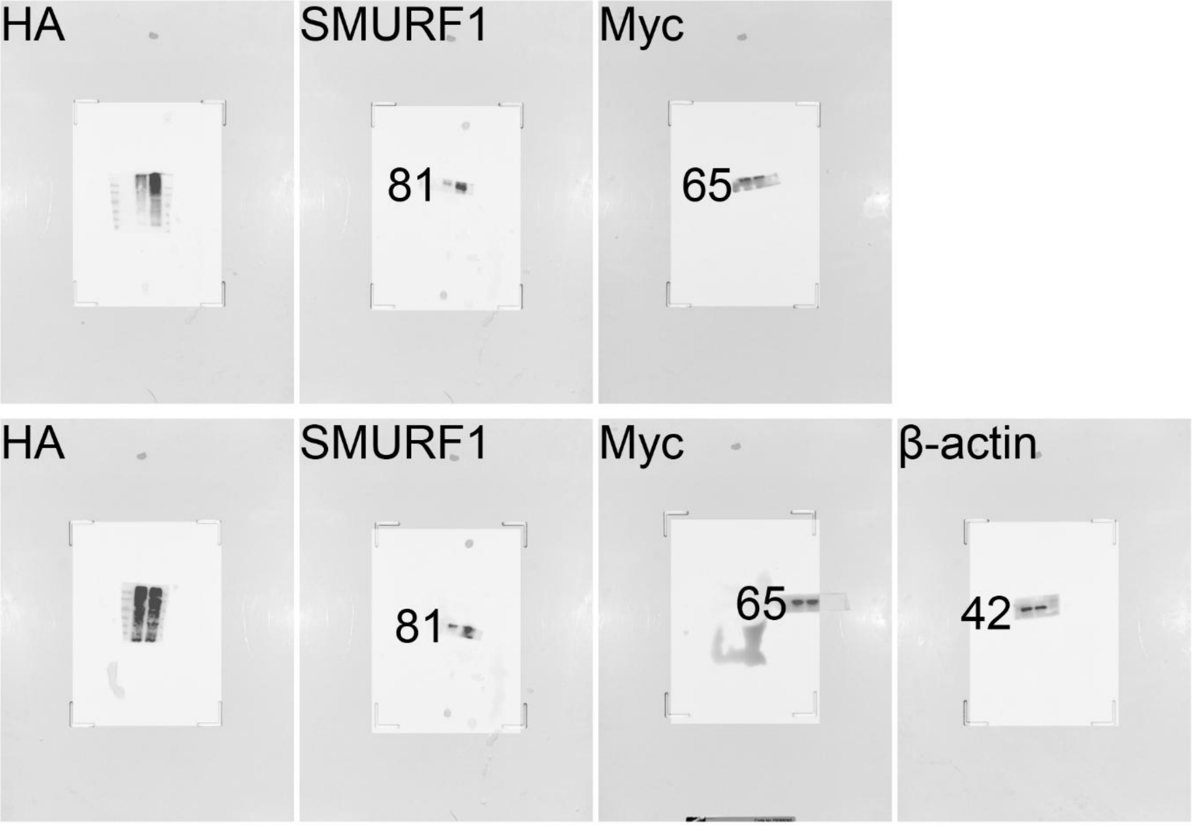

Figure 6B

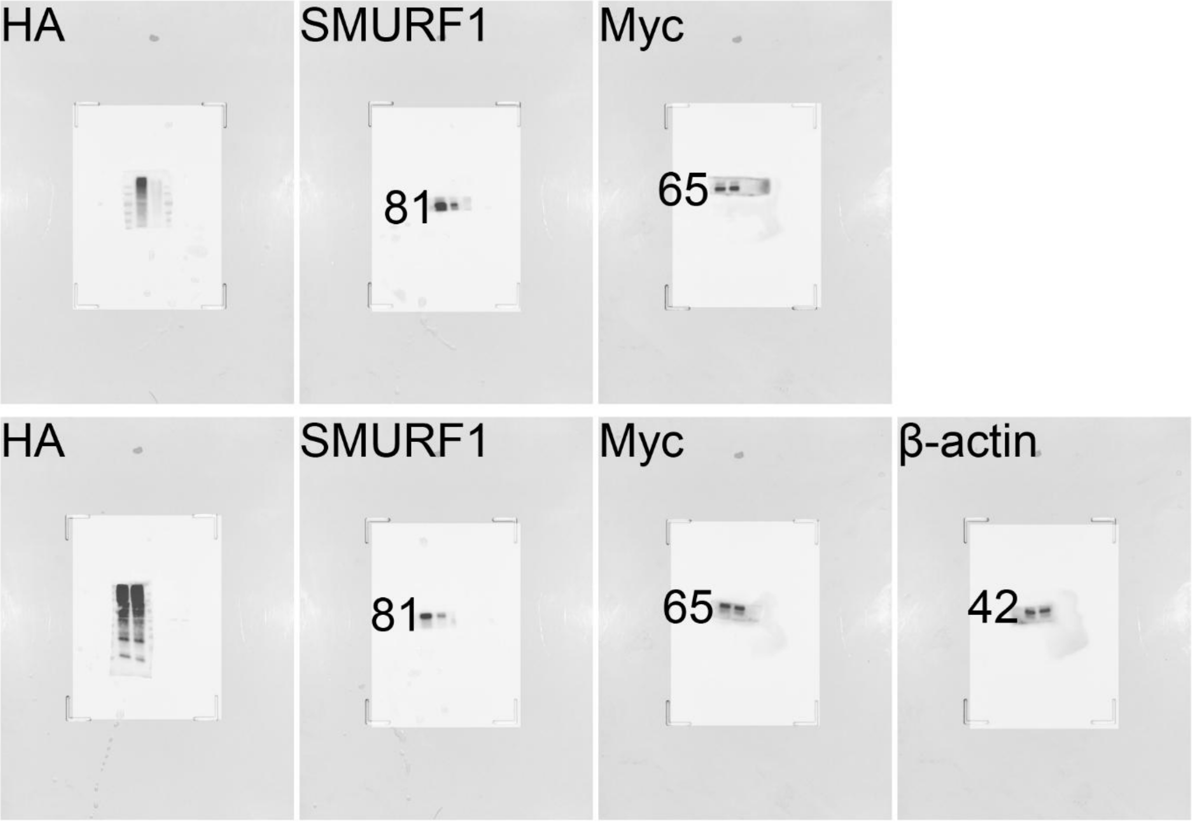

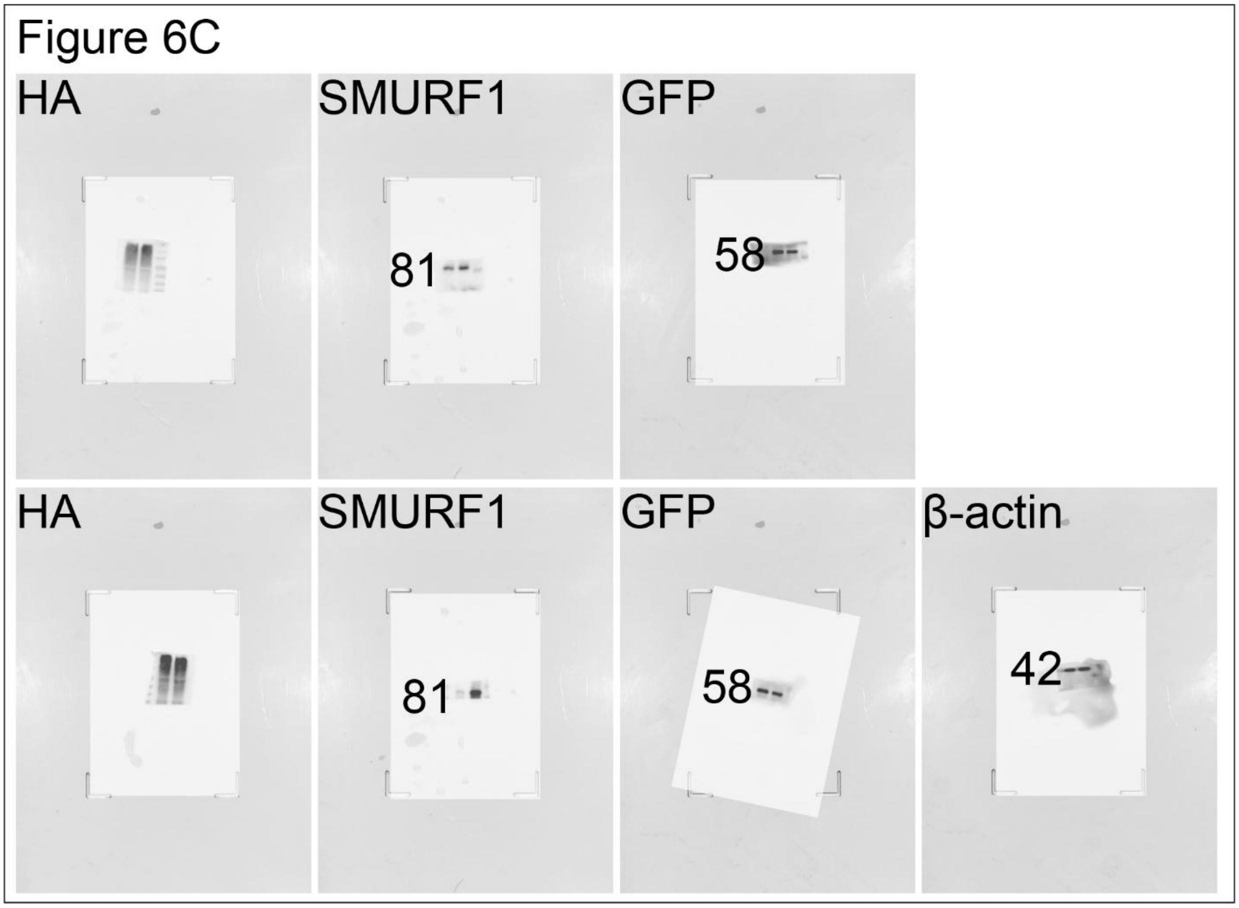
Figure 6C
HA
SMURF1
81
GFP
58
HA
SMURF1
81
GFP
58
β-actin
42

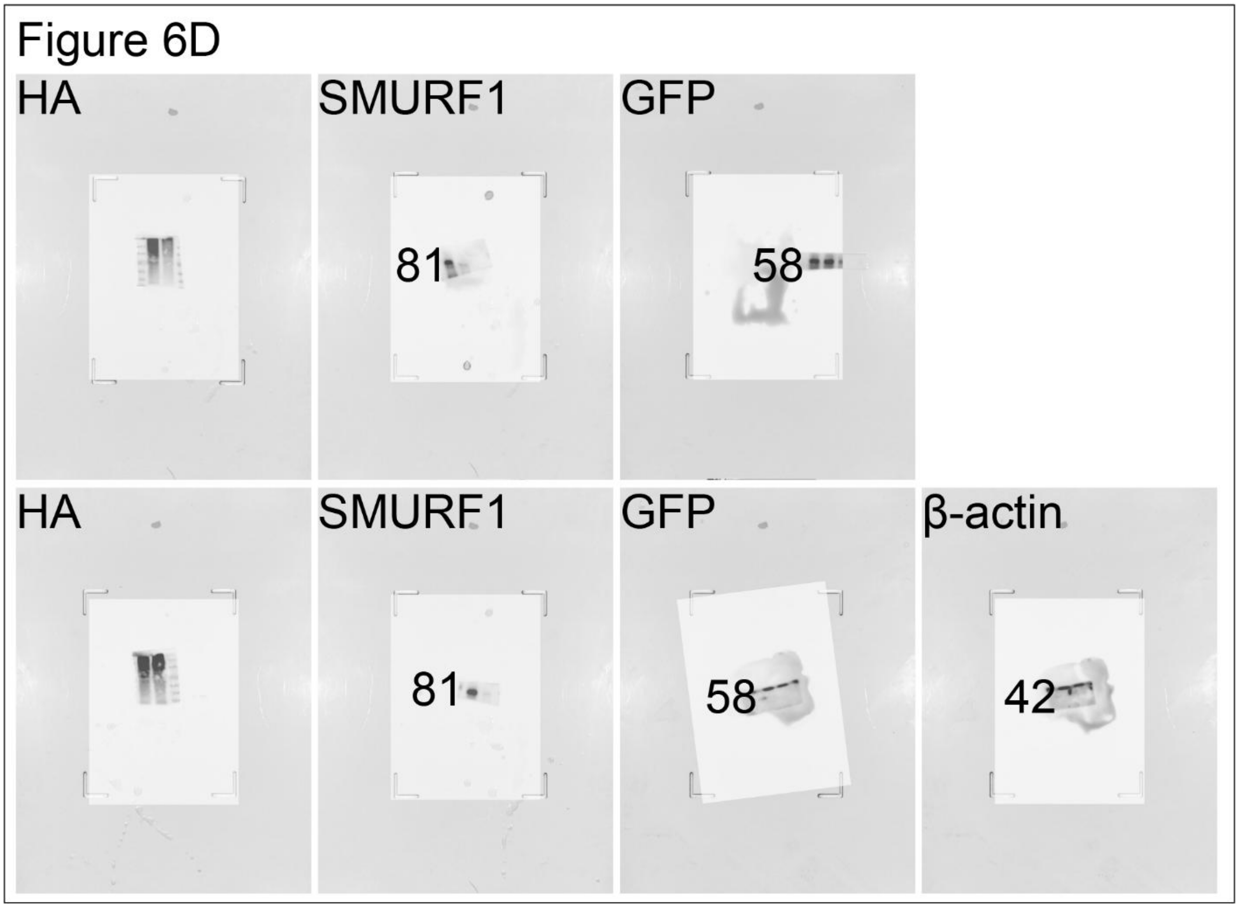
Figure 6D
HA
SMURF1
81
GFP
58
HA
SMURF1
81
GFP
58
β-actin
42

Figure 6E

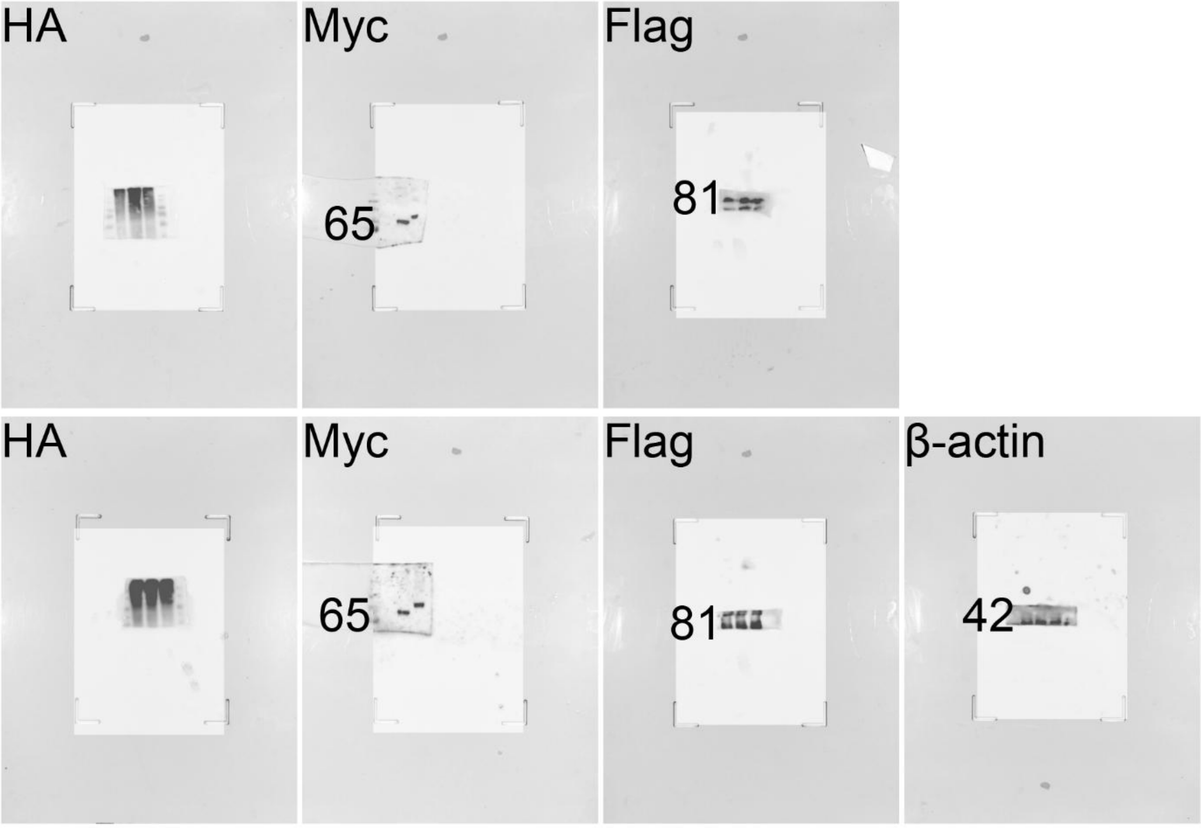

Figure 6F

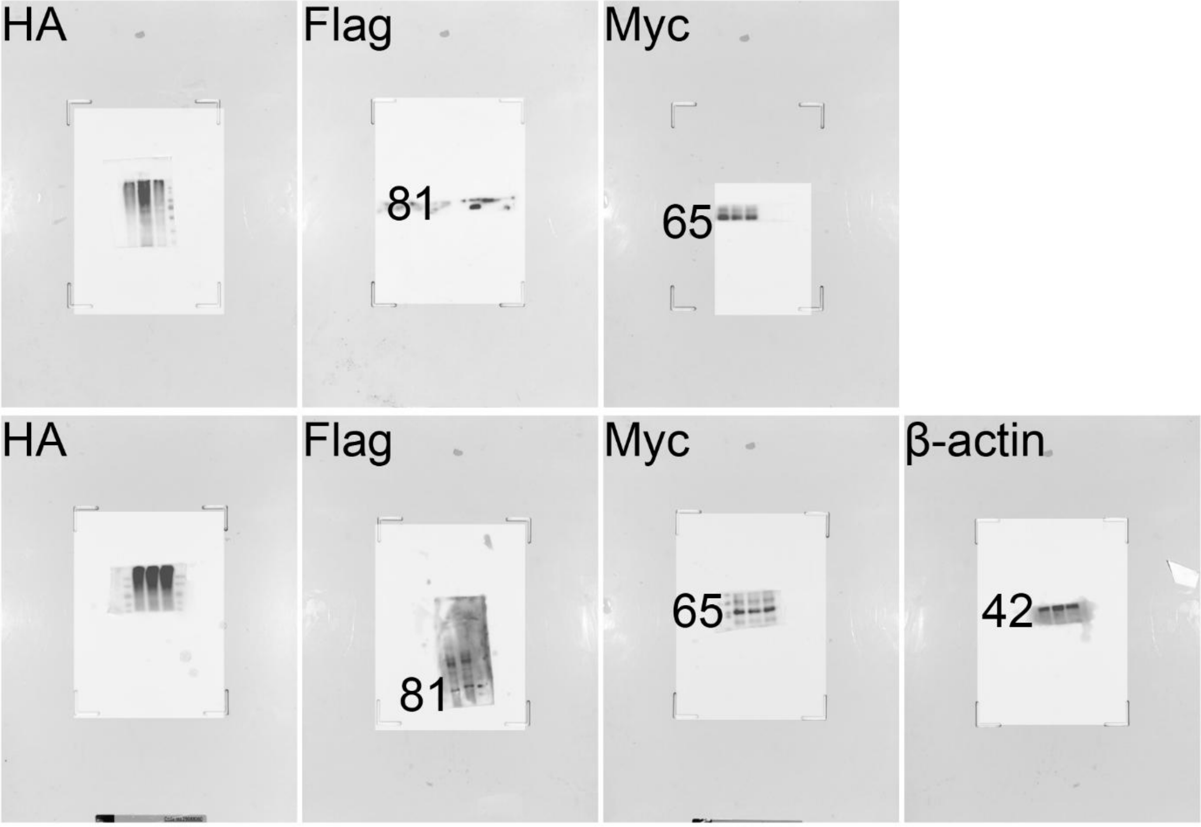

Figure 6G

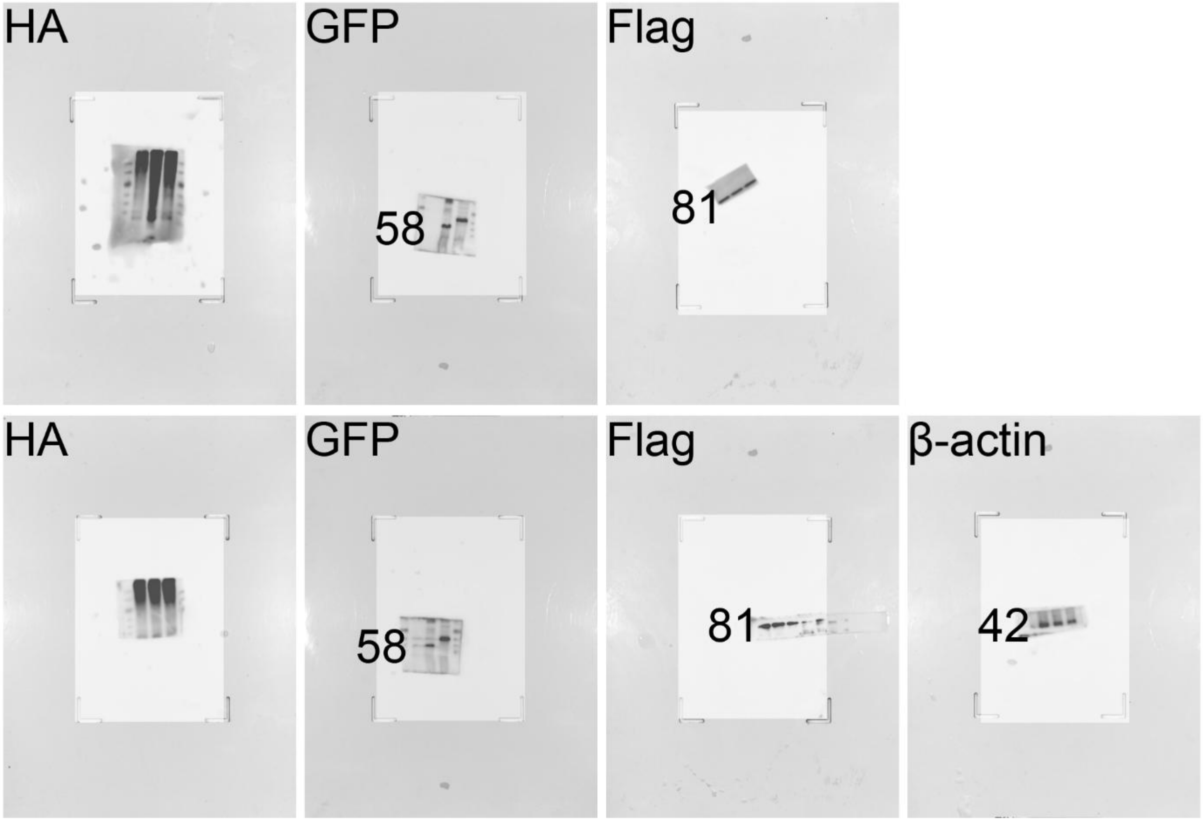

Figure 6H

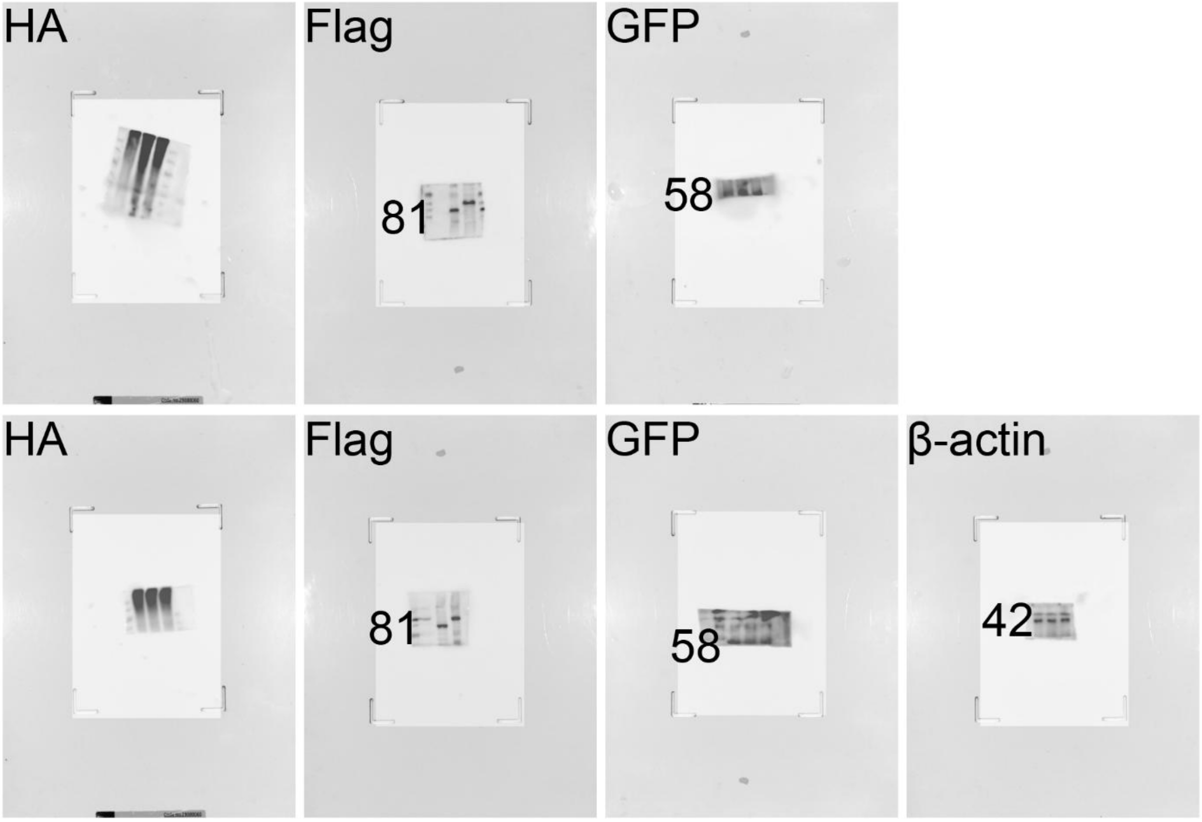

Figure 6I

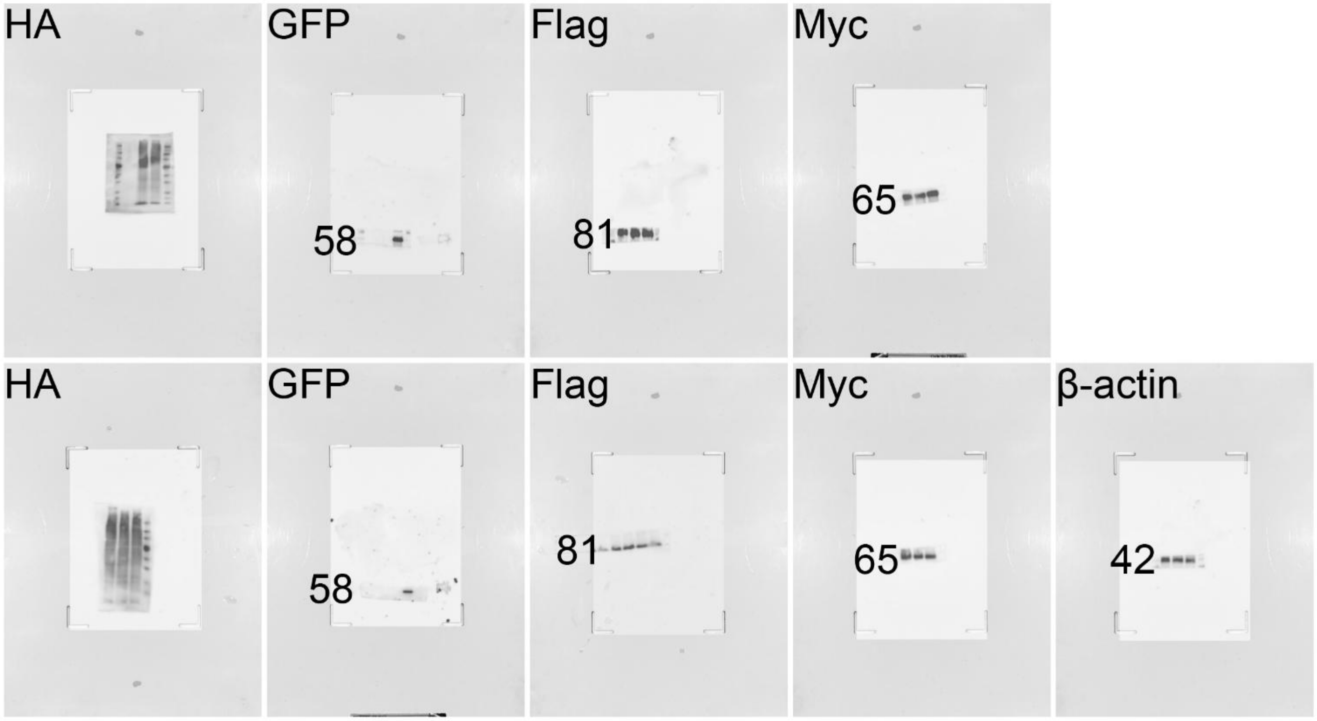

Figure 6I

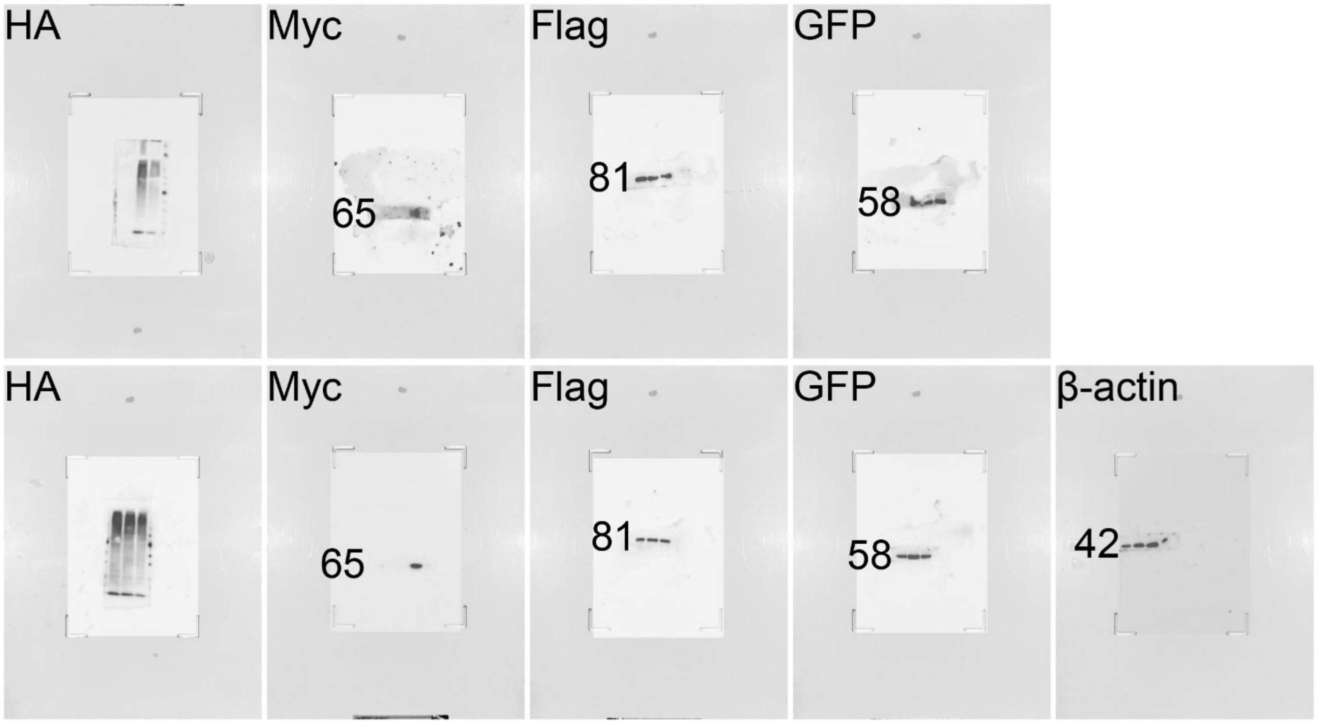

Figure 6J

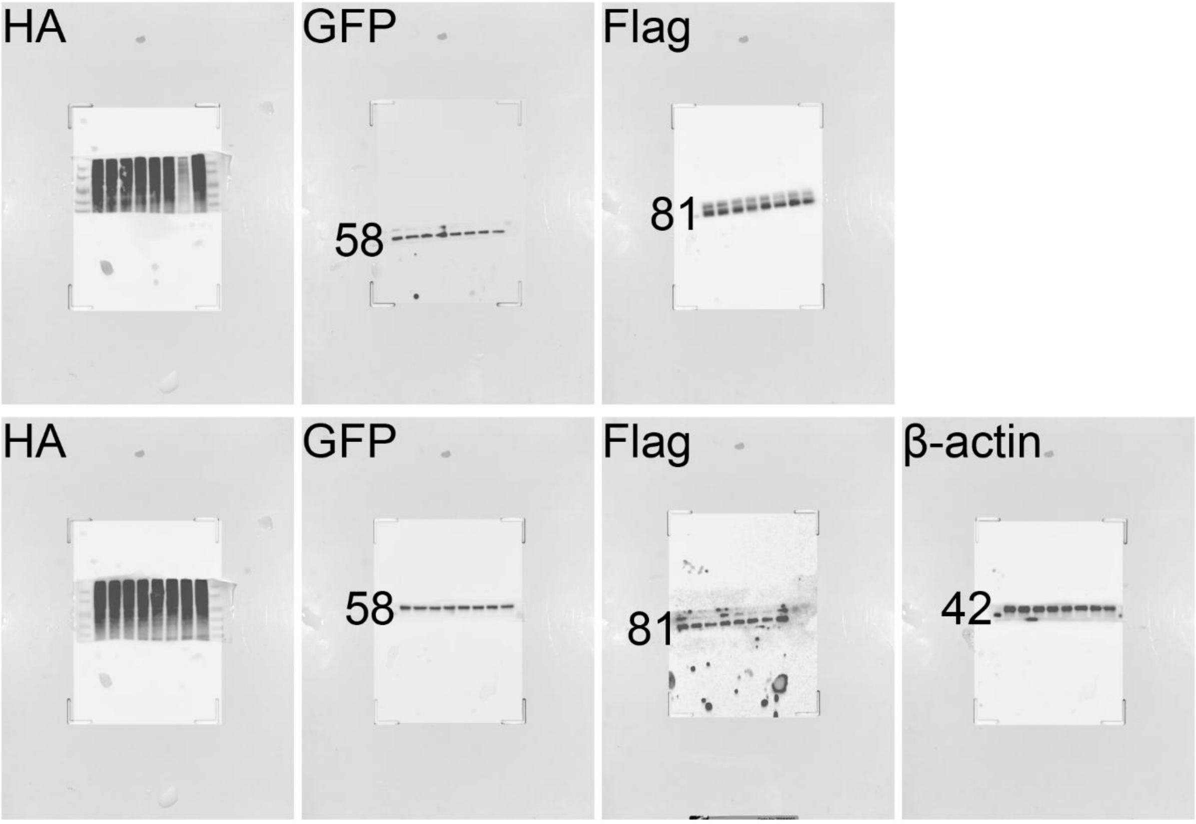

Figure 6K

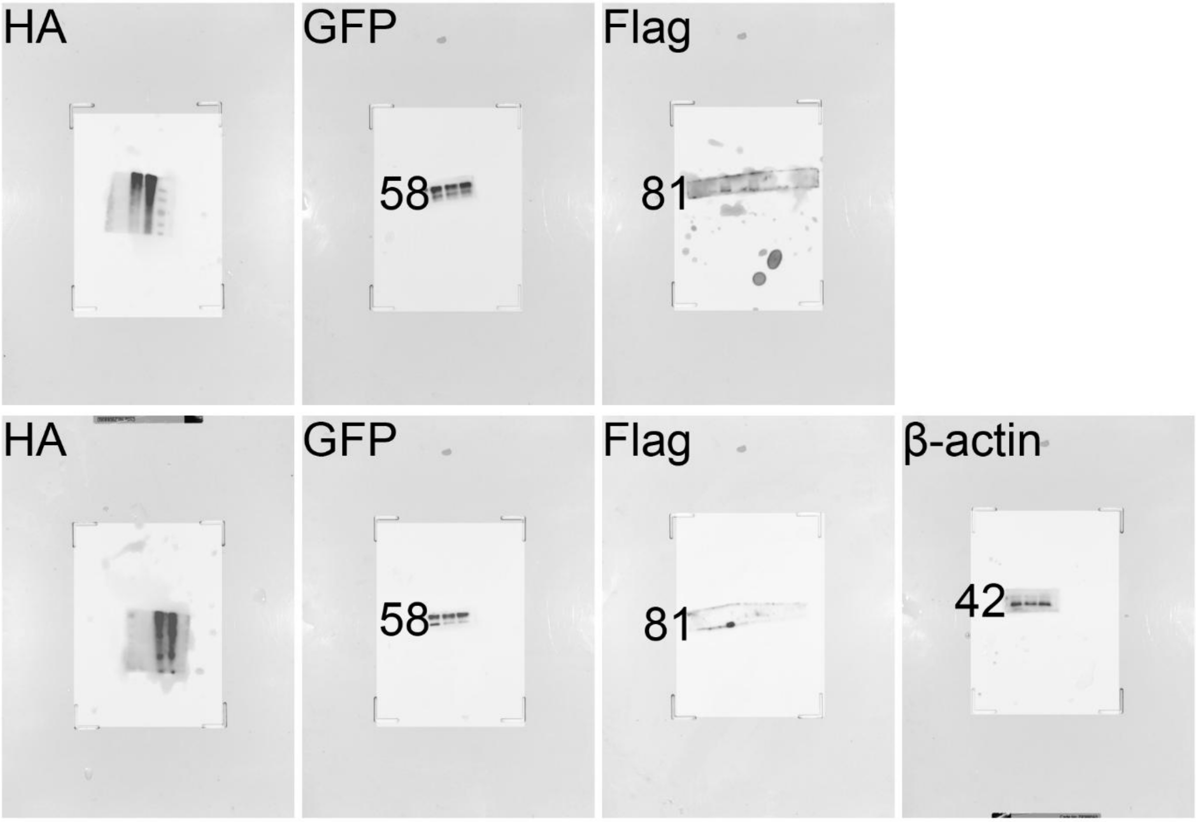

Figure 6L

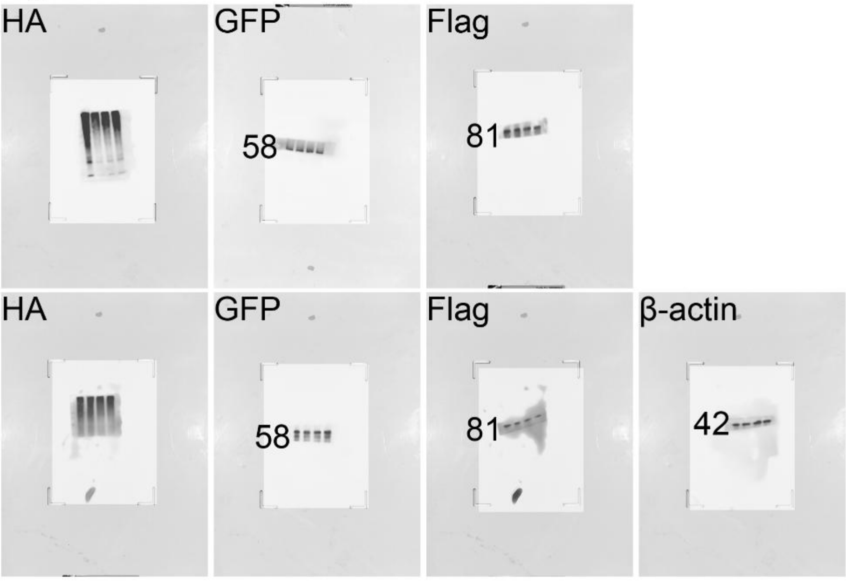

Figure 7B

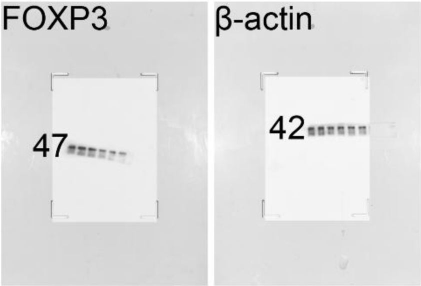

Figure 7F

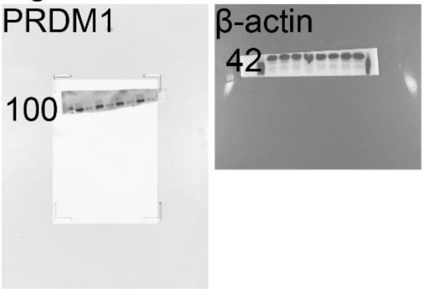

Figure 7H

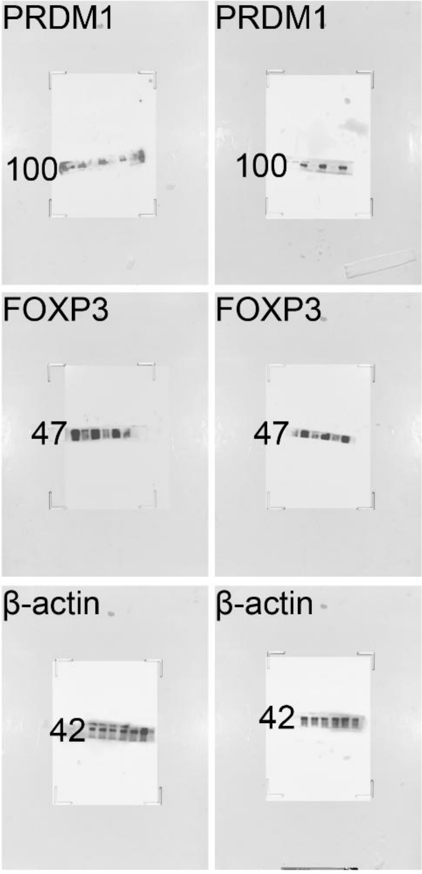

Figure 7Q

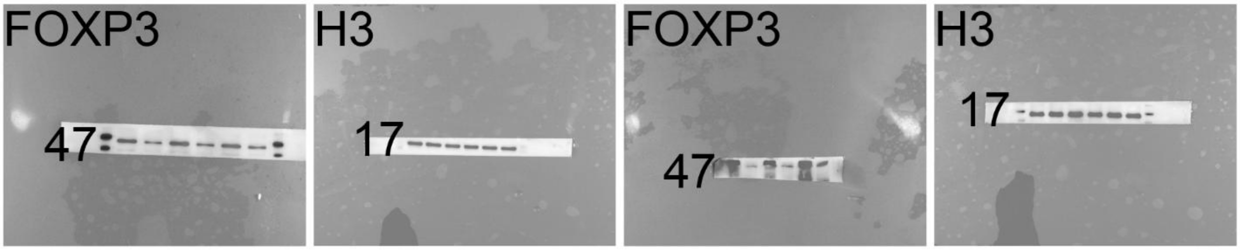

Figure 7Q

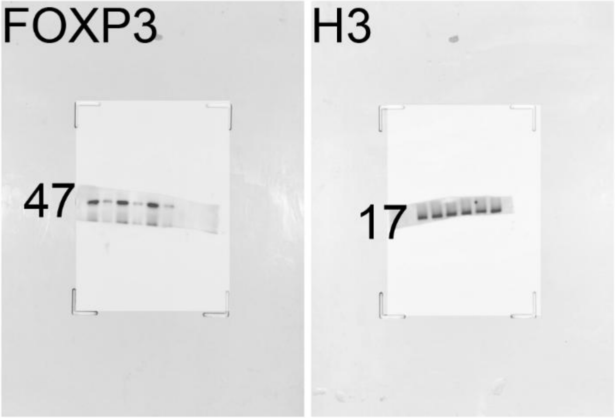

Figure 8D

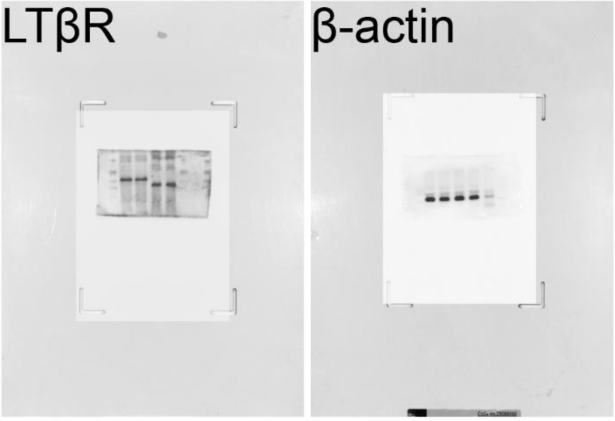

Figure 8B

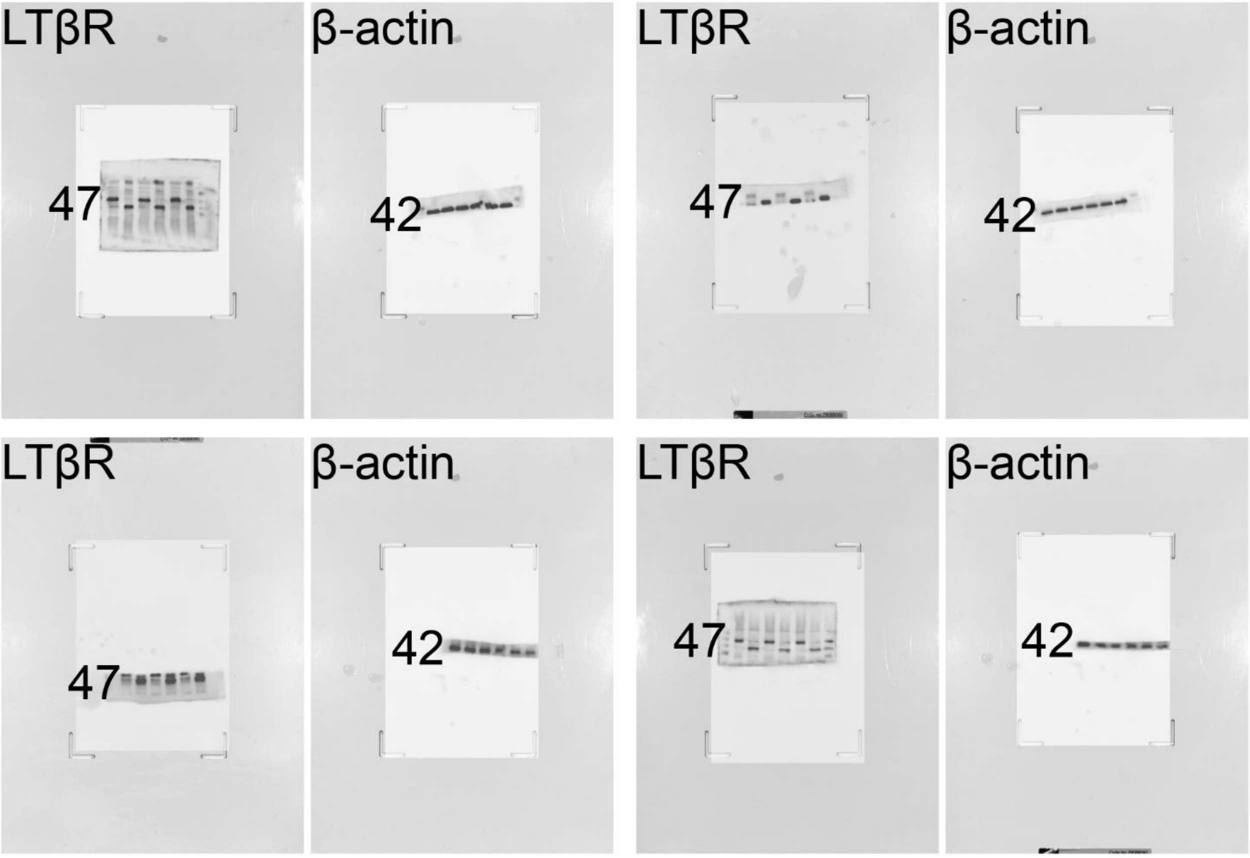

Figure 8G

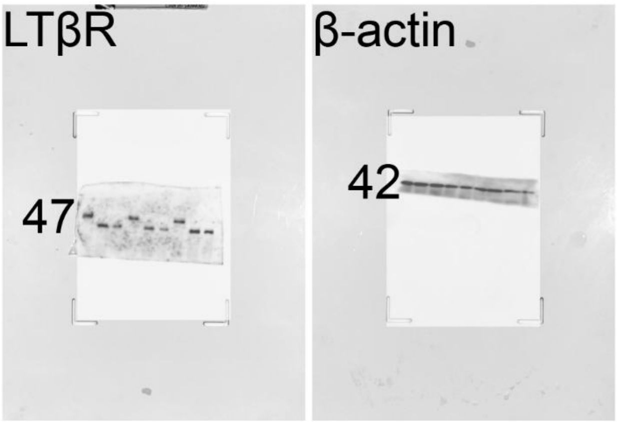

Figure 8H

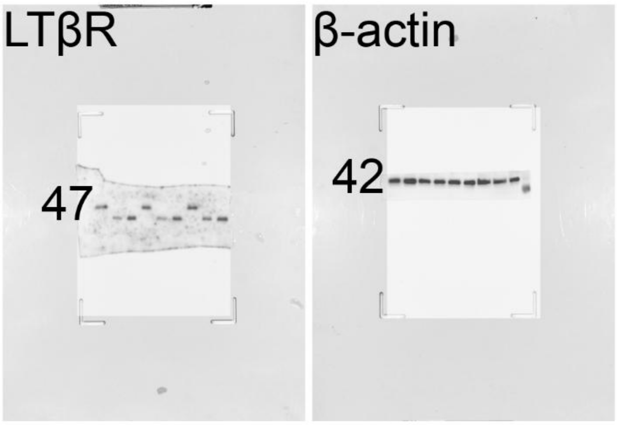

Figure 8I

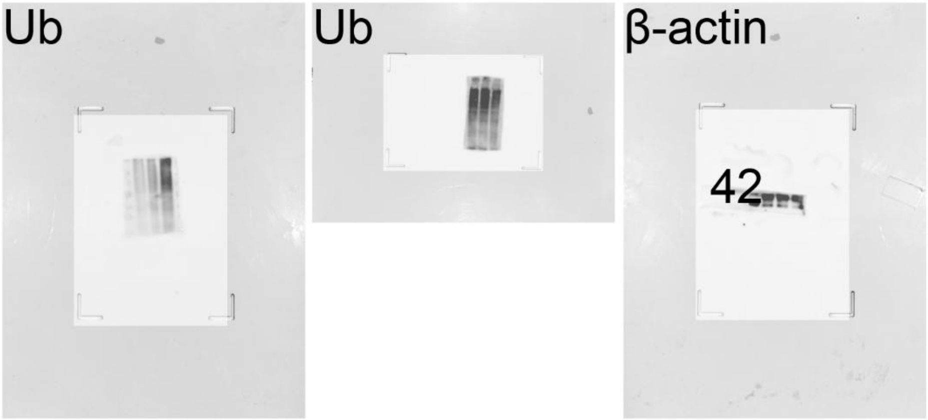

Figure S3C

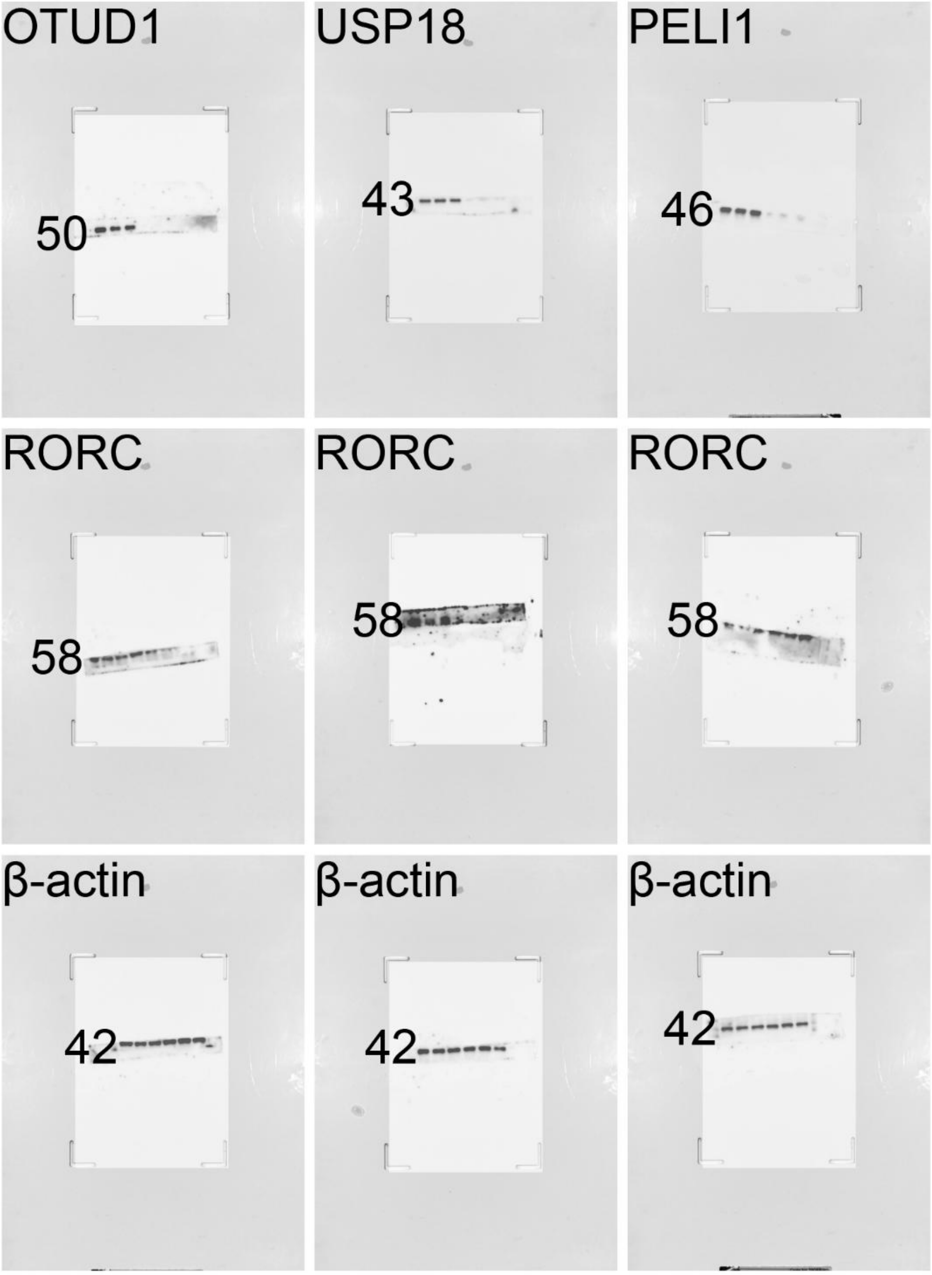

Figure S3E

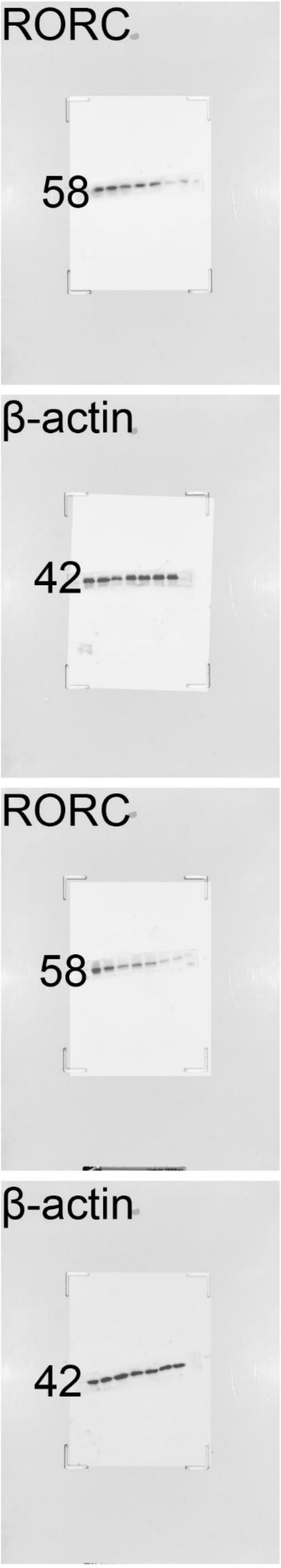

Supplement: Supplementary file 2 — Original western blots [file 41419_2025_7738_MOESM2_ESM.pdf]
